# Microginins from a *Microcystis* sp. Bloom Material Collected from the Kishon Reservoir, Israel

**DOI:** 10.3390/md16030078

**Published:** 2018-03-02

**Authors:** Anat Lodin-Friedman, Shmuel Carmeli

**Affiliations:** Raymond and Beverly Sackler School of Chemistry and Faculty of Exact Sciences, Tel Aviv University, Ramat-Aviv, Tel Aviv 69978, Israel; anatlodin@gmail.com

**Keywords:** microginins, cyanobacteria, *Microcystis*, aminopeptidase M inhibitors

## Abstract

During blooms, cyanobacteria produce diverse modified peptides. Among these are the microginins, which inhibit zinc-containing metalloproteases. Ten microginins, microginins KR767 (**1**), KR801(**2**), KR835 (**3**), KR785 (**4**), KR604 (**5**), KR638 (**6**), KR781 (**7**), KR815 (**8**), FR3 (**9**), and FR4 (**10**), were isolated from the extract of a bloom material of *Microcystis* sp. (IL-405) collected from the Kishon Reservoir, Israel in the fall of 2009. The structures of the pure compounds were elucidated using 1D and 2D NMR techniques and high-resolution mass spectrometry. The absolute configuration of the chiral centers of the amino acids were determined by Marfey’s and advance Marfey’s methods and by comparison of ^1^H and ^13^C NMR chemical shifts of the Ahda derivatives with those of known microginins. These microginins differ in sequence and absolute configuration of the chiral centers of the Ahda moieties and by *N*-methylation of the Ahda amine group and extent of chlorination of the Ahda terminal methyl group. The compounds were evaluated for inhibition of the zinc metalloprotease, aminopeptidase M, and exhibited low- to sub-nanomolar half maximal inhibitory concentration (IC_50_) values.

## 1. Introduction

Water-bloom forming cyanobacteria are prolific producers of diverse groups of highly active natural products [1]. Among the most frequently isolated are microcystins [2], micropeptins [3], aeruginosins [4], anabaenopeptins [5], microviridins [6], and microginins [7]. The microginins are linear peptides, characterized by N-terminal β-amino-α-hydroxy-decanoic or octanoic acid (in one case β-amino-decanoic) [8], which contain three to five additional amino acids or *N*-methylated amino acids [9]. The microginins inhibit zinc-containing metalloproteases, and their selectivity is determined by the sequence of the amino acids that are conjugated to the N-terminal β-amino-α-hydroxy-acid [10]. Thirty-one different microginins had been isolated and fully characterized by the end of 2016 (Table S1), and the use of sensitive mass spectroscopy (MS) methods, such as matrix-assisted laser desorption/ionization (MALDI) MS/MS and electrospray ionization (ESI) MS/MS, for the characterization of secondary metabolites of cyanobacterial bloom materials has enabled identified at least fifteen additional sequences of peptides belonging to the microginin group of metabolites [11,12]. These most recently identified microginins have been partially characterized with respect to their activities, the absolute configurations of the chiral centers of the amino acids, and the identities of *N*-methylated amino acids and aliphatic amino acids (i.e., *N*Me-valine versus leucine and isoleucine). The relative and absolute configurations of β-amino-α-hydroxy-decanoic (Ahda) or β-amino-α-hydroxy-octanoic acid (Ahoc) in the microginin are of great importance for biological activity [10], and three of the eight possible isomers are known to be produced by cyanobacteria. Compounds with similar planar structures but different relative and absolute configurations may not be distinguished by MS. Additional interesting aspects of the microginins are mono- or di-chlorination of the Ahda/Ahoa chain ends and *N*-methylation of the amino group of these acid residues. The reason that cyanobacteria produce so many derivatives of homologous peptides is unclear.

**Figure 1:**
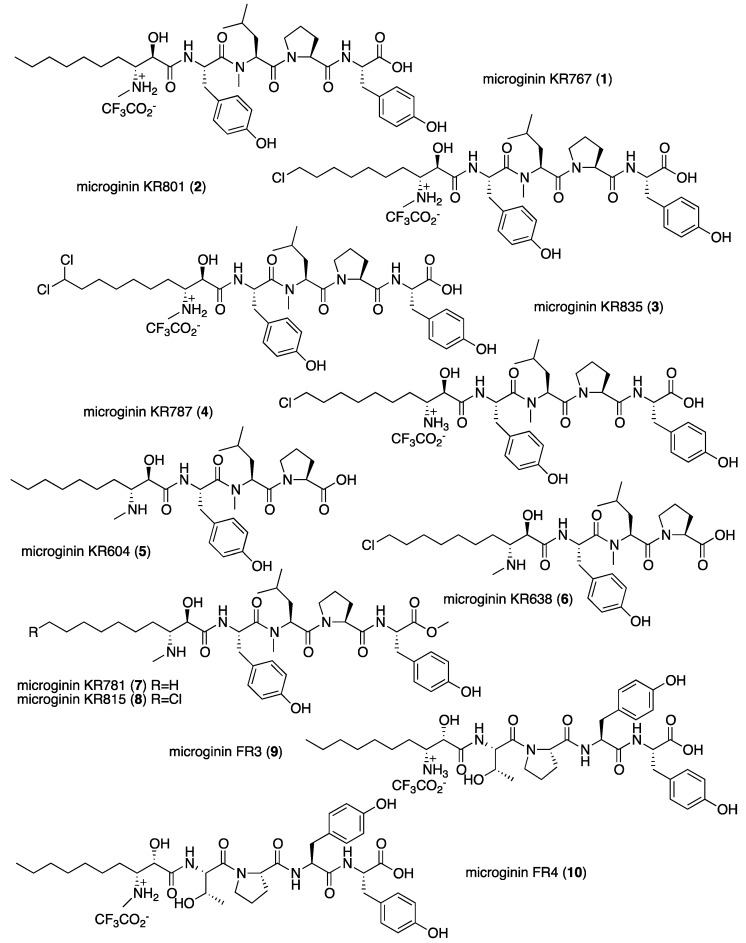
Microginins isolated from *Microcystis* sp. (Tel Aviv University (TAU) collection number: IL-405).

As part of our ongoing research on the chemistry and chemical ecology of cyanobacteria blooms in water bodies [13], a biomass of a bloom material of *Microcystis* sp. (Tel Aviv University (TAU) collection number: IL-405) was collected in November 2009 from the Kishon Reservoir, Israel. The extract of this bloom material afforded ten microginins. Six are novel: microginins KR767 (**1**), KR801 (**2**), KR835 (**3**), KR785 (**4**), KR604 (**5**) and KR638 (**6**). Two microginins—FR3 (**9**) and FR4 (**10**) [11]—had been previously characterized only by MS/MS and are fully characterized here for the first time. Microginins KR781 (**7**) and KR815 (**8**) are most probably isolation artifacts of microginins KR767 (**1**) and KR801 (**2**), respectively (Figure 1). The isolation, structure elucidations, and biological activities are discussed below.

## 2. Results and Discussion

Microginins **1**–**10** were isolated from aqueous-methanol extract of freeze-dried *Microcystis* sp. bloom material (collection number IL-405) by successive separations on a reversed-phase open column, size-exclusion on a Sephadex LH-20 column, and reversed-phase HPLC.

**Figure 2:**
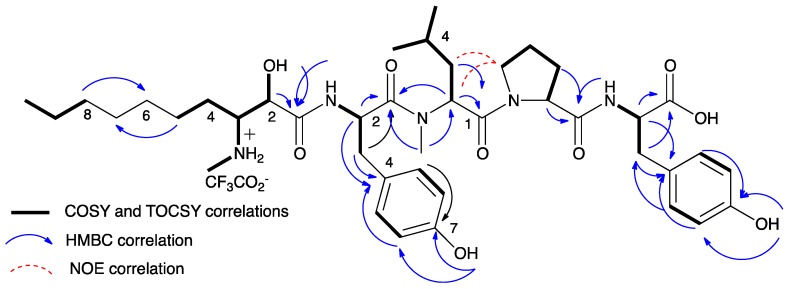
NMR correlations that supported the elucidation of the structure of microginin KR767 (**1**).

Microginin KR767 (**1**) presented a high-resolution (HR) ESI MS molecular ion adduct at *m*/*z* 768.4551, corresponding to the molecular formula C_41_H_61_N_5_O_9_, with 14 degrees of unsaturation. The ^1^H NMR spectrum (Table 1) revealed the presence of two singlet phenol protons (*δ*_H_ 9.25 and 9.20), five broad exchangeable protons (*δ*_H_ 8.49, 8.35, 8.10, 7.92, and 6.47), four doublet aromatic signals of two para-substituted phenol rings (*δ*_H_ 7.01, 7.00, 6.64, and 6.62, 2H each), eight protons next to heteroatoms (*δ*_H_ 5.23, 4.86, 4.31, 4.30, 4.27, 3.40, 3.27, and 3.18), two *N*Me signals (*δ*_H_ 2.89 brs and 2.56 brt), overlapping protons in the aliphatic region and three methyl signals (*δ*_H_ 0.84 t, 0.85 d, and 0.81 d). The ^13^C NMR spectrum (Table 2) of **1** revealed, among other peaks, signals due to five carbonyls (*δ*_C_ 173.1, 171.4, 171.0, 170.1, and 168.5), an oxymethine carbon (*δ*_C_ 68.5), five methine carbons (*δ*_C_ 60.3, 59.4, 54.0, 51.9, and 50.6), and one methylene carbon (*δ*_C_ 46.7) attached to amines, suggesting that **1** is a pentapetide secondary metabolite. The presence of the broad triplet *N*Me signal (*δ*_H_ 2.56, *J* = 4.7 Hz), which was coupled with two broad exchangeable protons at *δ*_H_ 8.49 and 8.35, and the presence of a shielded carboxyl-carbon at *δ*_C_ 173.1, suggest that **1** is a linear short peptide that occurs as a zwitterion of its N- and C-termini amino acids. The structures of the four proteinogenic amino acids, two Tyr, Pro, and *N*MeLeu, were established by a combination of COSY, HSQC, and HMBC 2D NMR experiments (Figure 2 and Table S2). The structure elucidation of the fifth amino acid, Ahda, was more challenging. The Ahda proton spin system, 2-OH, H-2, H-3 (3-NH_2_-Me), H_2_-4, H_2_-5, and H_2_-8, H_2_-9 H_3_-10, was established through COSY correlations. However, the connection of methylene-5 to methylene-6 through methylene-8 could not be determined by the COSY or HMBC correlations, although TOCSY correlations suggest that the latter two spin systems are connected. Counting the numbers of all of the atoms engaged in the proven proteinogenic amino acids and the latter two spin systems summed to C_39_H_57_N_5_O_9_, suggesting that methylene-5 is connected to methylene-8 through two additional methylenes. This was in agreement with the number of methylene carbons in ^13^C NMR and HSQC spectra. The connection of the five subunits to the planar structure of **1** was achieved through HMBC correlations observed between Ahda-CO and ^1^Tyr-NH, ^1^Tyr-CO and *N*MeLeu-H-2, and -*N*Me and both Pro-CO and ^2^Tyr-NH, and by NOE correlations between *N*MeLeu-H-2, H-3, and H-3′ and Pro-H-5 and H-5′ (Figure 2 and Table S2). The absolute configurations of the proteinogenic amino acids, *N*MeLeu, Pro, and Tyr were established by Marfey’s method [14] to be of the l configuration.

The absolute configuration of C-3 of *N*MeAhda was established as *R*, by applying the advanced Marfey’s method [15]. An attempt to establish the absolute configuration of C-2 in the unit by Mosher’s method [16] failed. H-2 in **1** appears as a broad singlet overlapping with Pro-H-2. Its splitting with 2-OH (*J* = 6.1 Hz), suggests that the *J*_2–3_ is small, and similar to the one measured for **4** (2.5 Hz), thus suggesting that the absolute configuration is either 2*S*,3*S*- or 2*R*,3*R* [10]. The data summarized in Table 3 for known compounds with proven absolute configurations at C-2 and C-3 of the Ahda derivatives, suggest that the proton and carbon chemical shifts are indicative of the relative configuration of these chiral centers and distinguish the 2*S*,3*S*- or 2*R*,3*R*-isomers from the 2*R*,3*S*- or 2*S*,3*R*-isomers. The chemical shifts of the latter carbons and protons of 2*S*,3*S*-Ahda and 2*R*,3*R*-Ahda of microginin 51-A and microginin 91-C [10], respectively, are almost identical (and the differences are due to dependent amino acids occupying the position next to the Ahda) and different from those of 2*S*,3*R*-Ahda in microginin [7]. The chemical shifts of H-2, C-2, H-3, and C-3 of **1** best matched those of microginin 51-B of characterized microginins, suggesting that the configuration of its chiral centers is either 2*S*,3*S*- or 2*R*,3*R*. Since the absolute configuration of C-3 was established as *R*, the absolute configuration of *N*MeAhda was assigned as 2*R*,3*R*. Based on these arguments, the structure of microginin KR767 was established to be **1** (Figure 1).

Microginin KR801 (**2**) had a complex HR ESI MS molecular ion at *m*/*z* 802.4166/804.4132 (3:1, [M + H]^+^) corresponding to the molecular formula C_41_H_60_ClN_5_O_9_ with 14 degrees of unsaturation. The ^1^H NMR spectrum (Table 1 and Table S2) of **2** was similar to that of **1**. However, in the spectrum of **2** a triplet methylene, a triplet of a triplet methylene, and a multiplet methylene, resonating at 3.60, 1.68, and 1.33 ppm, respectively, replaced resonances at 0.84, 1.24, and 1.20 ppm, corresponding to the terminal triplet methyl group of Ahda and two adjacent methylenes, respectively, observed in the spectrum of **1**. In the ^13^C NMR spectrum of **2** (Table 2 and Table S3), signals at 45.5, 32.2, and 26.3 ppm replaced the chemical shifts of the Ahda n-propyl chain end of **1** at 14.1, 22.2, and 31.4 ppm. These differences in the NMR data, together with the MS data, suggest that the chain terminating methyl of Ahda in **1** was substituted by a chloro-methylene in **2**. The structure of the Cl-Ahda moiety and of the rest of **2** were established unequivocally by COSY, HSQC, and HMBC correlations (Table S3). The absolute configurations of the chiral centers of **2** were established by Marfey’s method [14], advanced Marfey’s method [15], and comparison with the chemical shifts of Ahda derivatives (Table 3). Based on the data presented, structure **2** (Figure 1) was assigned to microginin KR801.

Microginin KR835 (**3**) displayed a complex HR ESI MS molecular ion at *m*/*z* 836.3765/838.3778/840.1901 (9:6:1, [M + H]^+^) corresponding to the molecular formula C_41_H_59_Cl_2_N_5_O_9_ with 14 degrees of unsaturation. The ^1^H NMR spectrum (Table 1 and Table S3) of **3** differed from those of **1** and **2** only in the signals of the *N*MeAhda moiety; the spectrum of **3** was characterized by a triplet methine at 6.29 ppm and two distinctive methylenes at 2.13 (dt) and 1.43 (tt) ppm. The ^13^C NMR spectrum (Table 2 and Table S3) presented significant changes in the chemical shifts of two carbons relative to those of **1** and **2** with resonances observed at 75.0 (CH) and 43.0 (CH_2_) ppm in the spectrum of **3**. These changes in the NMR spectra of **3**, relative to those of **1** and **2**, and the molecular formula of **3**, C_41_H_59_Cl_2_N_5_O_9_, were indicative of substitution of the *N*MeAhda chain end with two chlorine atoms. The structures of the Cl_2_-Ahda, two Tyr, *N*MeLeu, and Pro moieties and their assembly into the planar structure of **3** were established unequivocally by COSY, HSQC, HMBC, and ROESY correlations (Table S4). The absolute configurations of the chiral centers of **3** were established in the same manner as for **1**. On the basis of these arguments, structure **3** (Figure 1) was assigned for microginin KR835.

Microginin KR787 (**4**) was isolated as an amorphous white solid that had an HR ESI MS molecular ion at *m*/*z* 788.3954/790.3958 (3:1 [M + H]^+^) corresponding to the molecular formula C_40_H_58_ClN_5_O_9_ with 14 degrees of unsaturation. The ^1^H and ^13^C NMR spectra of **4** (Table 1 and Table 2) resembled those of **2** except for the absence of the NH_2_^+^Me group (*δ*_H_ 2.56 t, *δ*_C_ 30.8 CH_3_), and the replacement of two diasterotopic ammonium protons (*δ*_H_ 8.36 brs, 8.49 brs) in **2** by a signal resonating at *δ*_H_ 7.83 (brs) corresponded to three protons. The difference of 14 mass units between **2** and **4** and the differences in the NMR spectra suggest that the Cl-*N*MeAhda in **2** is replaced in **4** by a Cl-Ahda moiety. The structures of the five amino acids that compose **4**—Cl-Ahda, two Tyr, *N*MeLeu, and Pro and their assembly into the linear structure of **4** were established by analyses of the COSY, HSQC, HMBC, and ROESY spectra (Table S5). The absolute configuration of C-3 of Cl-Ahda was established as *R* by applying the advanced Marfey’s method [15]. The chemical shifts of C-2 and C-3 of 2*S*,3*S*-Ahda (70.4 and 52.9 ppm, respectively, in microginin 51-A [10] and 70.6 and 53.0 ppm, respectively, in microginin 299-C [8]) and of 2*R*,3*R*-Ahda (71.0 and 52.8, respectively, in microginin 91-C [10]) were similar, but were somewhat different from those of 2*S*,3*R*-Ahda (69.5 and 52.9, respectively, in microginin [7]) (Table 3). The chemical shifts of H-2 and H-3 in **4** matched those of microginin 51-A [10], which contains a Tyr next to the Adha moiety, but were different from those of microginin 91-C and microginin. Furthermore, the multiplicity and *J*-values of H-2 in **4** (*δ*_H_ 4.14 dd, *J* = 5.4, 2.5 Hz) matched those of microginin 299-C (*δ*_H_ 4.20 dd, *J* = 5.6, 3.0 Hz) [8] and differed from those of microginin FR3 (**9**) (*δ*_H_ 4.10 t, *J* = 4.6 Hz). Thus, the absolute configuration of the Cl–Ahda chiral centers were established as 2*R*,3*R*, and microginin KR787 (**4**) has the structure shown in Figure 1.

Microginin KR604 (**5**), an amorphous white solid, exhibited an HR ESI MS molecular ion at *m*/*z* 603.3764 [M − H]^−^ corresponding to the molecular formula C_32_H_52_N_4_O_7_ with nine degrees of unsaturation. The difference in the molecular formulas between **1** and **5**, C_9_H_9_NO_2_, suggests that **5** lacks a terminal tyrosine unit. The ^1^H and ^13^C NMR spectra of **5** (Table 1 and Table 2) were comparable to those of **1**, with the exceptions of the regions of the signals of the terminal tyrosine unit of **1**. Analyses of the COSY, HSQC, HMBC, and ROESY spectra of **5** (Table S6) established the structure of the subunits and their sequence: *N*MeAdha-Tyr-*N*MeLeu-Pro. The absolute configuration of the chiral centers of the four subunits was determined as described for **1**, establishing the structure of microginin KR604 as **5** (Figure 1).

Microginin KR638 (**6**) had a complex molecular adduct ion [M + H]^+^ at *m*/*z* 639.3521/641.3527 (3:1) in the HR ESI mass spectrum corresponding to the molecular formula C_32_H_51_ClN_4_O_7_ with nine degrees of unsaturation. Comparing the ^1^H and ^13^C NMR spectra (Table 1 and Table 2) and molecular formula of **6** with those of **1**, **2**, and **5** suggested that **6** is the Cl-*N*MeAhda derivative of **5**. The sequence of **6**, Cl*-N*MeAdha-Tyr-*N*MeLeu-Pro, and the structures of the four substructures that compose **6** were established by analyses of COSY, HSQC, HMBC, and ROESY (Table S7). The absolute configuration of the chiral centers of microginin KR638 were determined by the same procedures described above, establishing structure **6** (Figure 1).

Microginins KR781 (**7**) and KR815 (**8**) exhibited HR ESI MS molecular ions at *m*/*z* 780.4553 [M − H]^−^ and 816.4318/818.4380 (3:1) [M + H]^+^, respectively, corresponding to the molecular formulas of C_42_H_63_N_5_O_9_ and C_42_H_62_N_5_O_9_, respectively. Their ^1^H and ^13^C NMR spectra (Table 1, Table 2, Table S8 and Table S9) resembled those of **1** and **2** except for extra methoxy group signals (*δ*_H_ 3.53 s, *δ*_C_ 51.9 CH_3_ for **7** and *δ*_H_ 3.53 s, *δ*_C_ 51.8 CH_3_ for **8**). Full NMR assignments and determination of the absolute configurations of the chiral centers established structures **7** and **8** for microginins KR787 and KR815, respectively (Figure 1). Microginins KR787 (**7**) and KR815 (**8**) are methyl ester derivatives of microginins KR767 (**1**) and KR801 (**2**), respectively.

**Figure 3:**
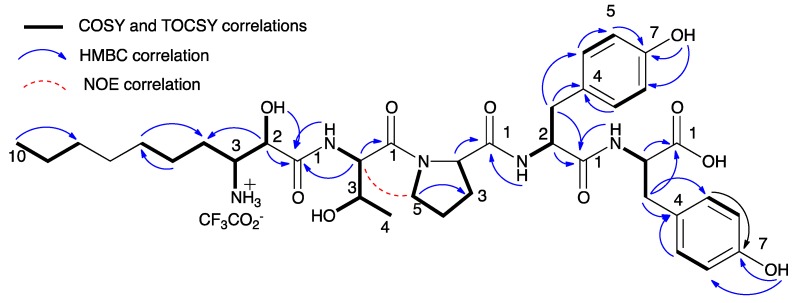
NMR correlations that supported the elucidation of the structure of microginin FR3 (**9**).

Microginin FR3 (**9**) was isolated as an amorphous white solid. It presented an HR ESI MS molecular adduct ion [M + H]^+^ at *m*/*z* 728.3874 corresponding to the molecular formula C_37_H_53_N_5_O_10_ with 14 degrees of unsaturation. The NMR spectra of **9** (Table 1, Table 2 and Table S10) indicated the presence of an aliphatic chain and two *para*-substituted phenol rings, suggesting that **9** belongs to the microginin type of metabolites. Comparison of the NMR spectra of **9** and **1** suggested that these two compounds have different amino acid compositions (Table 1). In the ^1^H NMR spectrum of **9**, there was no indication of aliphatic methyls or *N*-methyls, which were found in **1**. Analysis of the NMR data (^1^H, ^13^C, HSQC, HMBC, COSY, TOCSY, and ROESY) of **9** established the structures of five subunits and their sequence: Ahda-Thr-Pro-^1^Tyr-^2^Tyr (Table S10 and Figure 3). The sequence of **9** is identical to the one established by MS/MS for microginin FR3 collected from bloom materials in Germany [11]. The absolute configurations of the proteinogenic amino acids Thr, Pro, and Tyr were established by Marfey’s method [14] to be of l configurations. The absolute configuration of C-3 of Ahda was established as *R* by applying the advanced Marfey’s method [15]. As for **1**–**8**, the absolute configuration of the Ahda-2-chiral center was proven by comparison of the chemical shifts of H-2, C-2, H-3, and C-3 of **9** with those of microginins for which the absolute configuration has been established unequivocally (Table 3). The chemical shifts of carbons and protons of Ahda in **9** matched those of 2*S*,3*R*-Ahda in a previously reported microginin [7]. Interestingly, the absolute configurations of C-2 and C-3 in **9** were identical to those of microginin FR1, which were established by X-ray diffraction analysis [18]. Based on this evidence, the structure of **9** was established (Figure 1) and shown to be that of microginin FR3.

Microginin FR4 (**10**), an amorphous white solid, had an HR ESI MS molecular cluster ion at *m*/*z* 742.4031, corresponding to the molecular formula C_38_H_55_N_5_O_10_ with 14 degrees of unsaturation. Comparison of the ^1^H and ^13^C NMR spectra (Table 1 and Table 2) of **10** with those of **9** revealed signals of an additional *N*Me (*δ*_H_ 2.50 brs, *δ*_C_ 31.3 CH_3_) and differences in the chemical shifts of the ammonium signals (*δ*_H_ 8.36 brs and 8.17 brs) and a methine (*δ*_H_ 3.30 m, *δ*_C_ 60.1 CH). The differences between **9** and **10** in the NMR spectra and in molecular formulas suggest that the Adha ammonium group in **9** is substituted by a methyl ammonium group in **10**. Full analysis of the NMR data (^1^H, ^13^C, HSQC, HMBC, COSY, TOCSY, and ROESY) of **10** established the structures of the five subunits and the sequence, *N*MeAdha-Thr-Pro-^1^Tyr-^2^Tyr (Table S11). The absolute configurations of the proteinogenic amino acids, Thr, Pro, and Tyr, and of C-3 of *N*Me-Ahda were established by the same procedures described for **9**. In **10**, the proteinogenic amino acids are in the l configuration and the *N*MeAhda is 3*R*. Unfortunately, the chemical shifts of *N*MeAhda C-2, C-3, H-2, and H-3 did not match those of the Ahda moieties of microginins with proven absolute configurations (Table 3). The structural similarities of **9** and **10** and the similar splitting of the Ahda H-2 (*δ*_H_ 4.10 t, *J* = 4.6 Hz in **9**; *δ*_H_ 4.18 t, *J* = 4.4 Hz in **10**), which differed from those of **2** and **4** (*δ*_H_ 4.30 brd, *J* = 5.6 Hz in **2**; *δ*_H_ 4.14 dd, *J* = 5.4, 2.5 Hz in **4**), suggest that Ahda moieties of **9** and **10** share the same absolute configuration. Furthermore, comparison of the changes in the chemical shifts of C-2, C-3, H-2, and H-3 in pairs of compounds with Ahda/*N*MeAhda and defined absolute configurations of 2*S*,3*S* (microginin 51-A and microginin 51-B [10], ∆*δ*_C-2_ + 2.3, ∆*δ*_C-3_ − 6.9, ∆*δ*_H-2_ − 0.14, ∆*δ*_H-3_ + 0.02 ppm) and 2*R*,3*R* (**2** and **4**, ∆*δ*_C-2_ + 2.1, ∆*δ*_C-3_ − 7.1, ∆*δ*_H-2_ − 0.15, ∆*δ*_H-3_ 0 ppm) with those of **9** and **10** (∆*δ*_C-2_ + 1.4, ∆*δ*_C-3_ − 6.9, ∆*δ*_H-2_ − 0.08, ∆*δ*_H-3_ − 0.08 ppm) suggest that the absolute configurations of the chiral centers of Ahda moieties in **9** and **10** are different from those of **1**–**8** and from those of microginins 51-A and 51-B. These findings, together with the 3*R* configuration established for the *N*MeAhda, lead us to suggest the 2*S*,3*R* absolute configuration for *N*MeAhda in **10**. Based on these arguments, structure **10** (Figure 1) is proposed to be that of the previously identified microginin, FR4 [11].

The biological activities of newly characterized **1**–**6** and **8**–**10** were examined against the metalloprotease, aminopeptidase M. The results are summarized in Table 4 and are comparable with those published for other microginins [10]. Examination of the results of the inhibition assay allowed us to draw some structure–activity relationships. Comparison of the half maximal inhibitory concentration (IC_50_) values of microginins, KR801 (**2**) and KR815 (**8**), revealed that the terminal carboxylic acid is essential for the inhibitory activity. The truncated peptide microginins, KR604 (**5**) and KR638 (**6),** are an order of magnitude less active than microginins KR767 (**1**), KR801 (**2**), and KR835 (**3**). The *N*MeAhda-containing microginins, KR801 (**2**) and FR4 (**10**) are more effective inhibitors of aminopeptidase M than their Adha-containing counterparts, microginins KR787 (**5**) and FR3 (**9**). Comparison of the inhibitory activities of microginins KR767 (**1**) and FR4 (**10**) suggests that the sequence *N*MeAdha-Tyr-*N*MeLeu-Pro-Tyr is more effective than *N*MeAdha-Thr-Pro-Tyr-Tyr in inhibiting the proteolytic activity of aminopeptidase M.

## 3. Materials and Methods

### 3.1. General Experimental Procedures

Optical rotation values were obtained on a Jasco P-1010 polarimeter (Jasco, Oklahoma City, OK, USA) at the sodium D line (589 nm). UV spectra were recorded on an Agilent 8453 spectrophotometer (Agilent, Santa Clara, CA, USA). IR spectra were recorded on a Bruker Tensor 27 FT-IR instrument (Bruker, Billerica, MA, USA). NMR spectra were recorded on Bruker Avance and Avance III spectrometers (Bruker, Karlsruhe, Germany) at 500.13 MHz for ^1^H and 125.76 MHz for ^13^C. DEPT, COSY-45, gTOCSY, gROESY, gHSQC, gHMQC, and gHMBC spectra were recorded using standard Bruker pulse sequences (Bruker, Karlsruhe, Germany). NMR chemical shifts were referenced to TMS *δ*_H_ and *δ*_C_ = 0 ppm. HR ESI MS were recorded on a Waters MaldiSynapt instrument (Waters, Milford, MA, USA), and LC ESI MS spectra were recorded on a Waters Xevo TQD instrument (Waters, Milford, MA, USA). HPLC separations were performed on a Merck Hitachi HPLC system (L-6200 Intelligent pump and L-4200 UV-VIS detector, Hitachi, Tokyo, Japan), a JASCO P4-2080 plus HPLC system with a multiwavelength detector (Jasco International, Tokyo, Japan), and an Agilent 1100 Series HPLC system (Agilent, Santa Clara, CA, USA).

### 3.2. Biological Material

*Microcystis* sp. (collection No. IL-405) was collected from the Kishon reservoir in November 2009. The sample was identified by microscopic observation as a *Microcystis* sp. A lyophilized voucher sample (IL-405) was deposited in the culture collection of Tel Aviv University (Tel Aviv, Israel).

### 3.3. Isolation Procedure

The freeze-dried cell mass (2295 g) was extracted with 7:3 MeOH:H_2_O (3 × 5 L). The crude extract was evaporated to dryness. Fatty acids and salts were removed from the crude extract with petroleum ether and methanol, respectively, to yield a secondary extract (70 g). Aliquots of the secondary extract were fractionated (5 g in each separation) on a reversed-phase flash column (ODS, YMC-GEL, 120 Å, 4.4 cm × 6.4 cm, YMC Co., Kyoto, Japan) with increasing concentration (10% step-gradient from 0 to 100%) of MeOH in H_2_O. Combined fraction 6 (F6, 1.4 g, 1:1 MeOH:H_2_O) and fraction 7 (F7, 1.4 g, 3:2 MeOH:H_2_O) were further separated on a Sephadex LH-20 size-exclusion column. F6 was eluted with 7:3 MeOH:H_2_O solution to afford eleven fractions, F6a to F6k. Combined fractions F6a and F6b (F6ab, 452 mg) were re-fractionated on the same Sephadex LH-20 column eluted with 1:1 MeOH:H_2_O solution to afford seventeen fractions (F6ab1 to F6ab17). Combined fractions F6ab7 to F6ab9 (43.2 mg) were separated on a reversed phase HPLC column (YMC-Pack C-8, 250 mm × 20 mm, 5 μm, YMC Co., Kyoto, Japan) with an isocratic solvent system of 42% MeCN/58% 0.1% aq. TFA to yield six fractions, F6ab7-9a to F6ab7-9f. Fraction F6ab7-9c (35.6 mg) was separated on the same column eluted using 26% MeCN/74% 0.1% aq. TFA to afford six fractions (F6ab7-9c1 to F6ab7-9c6). Fraction F6ab7-9c1 was found to be pure microginin KR638 (**6**) (*R*_t_ 54.4 min, 2.1 mg, 9.2 × 10^−5^% yield based on the dry weight of the cells). Fraction F6ab7-9c2 (5.7 mg) was purified on the same column eluted with 45% MeCN/55% 0.1% aq. TFA to yield, in the second fraction, pure microginin KR604 (**5**) (*R*_t_ 16.7, 2.2 mg, 9.6 × 10^−5^% yield). Fraction 6c (F6c, 361 mg) was separated on the reversed phase HPLC column (YMC-Pack C-8, 250 mm × 20 mm, 5 μm) eluted with an isocratic solvent system of 40% MeCN/60% 0.1% aq. TFA to yield eleven fractions (F6c1 to F6c11). Fraction F6c5 (57.2 mg) was fractionated on the same HPLC column with 45% MeCN/55% 0.1% aq. TFA to yield four fractions, two of which (fractions 3 and 4) were found to be pure microginin KR815 (**8**) (*R*_t_ 38.3 min, 2.0 mg, 8.7 × 10^−5^% yield) and microginin KR781 (**7**) (*R*_t_ 41.1 min, 1.1 mg, 4.8 × 10^−5^% yield), respectively. Fraction F6d (363 mg) was separated on the same C-8 HPLC column using 33% MeCN/67% 0.1% aq. TFA as eluent to yield twelve fractions, F6d1 to F6d12. Fraction F6d6 (7.9 mg) was further purified on the same column eluted with 25% MeCN/75% 0.1% aq. TFA to yield pure microginin FR3 (**9**) (*R*_t_ 42.6 min, 2.2 mg, 9.6 × 10^−5^% yield). Fraction F6d7 (6.5 mg) was further purified on the same column eluted with 26% MeCN/74% 0.1% aq. TFA to yield, in the second fraction, pure microginin FR4 (**10**) (*R*_t_ 41.9 min, 1.9 mg, 8.3 × 10^−5^% yield). Fraction F6d10 (7.4 mg) was further purified on the same column eluted with 32% MeCN/68% 0.1% aq. TFA to yield pure microginin KR787 (**4**) (*R*_t_ 56.5 min, 2.5 mg, 1.09 × 10^−4^% yield). Fraction 7 was separated on Sephadex LH-20 column eluted with 1:1 MeOH:H_2_O solution to afford ten fractions, F7a to F6j. Combined fractions F7b to F7d (646 mg) were separated again on the Sephadex LH-20 column using 7:3 MeOH:H_2_O as eluent to yield thirteen fractions, F7b-d1 to F7b-d13. Combined fractions F7b-d7 and F7b-d8 (99.3 mg) were separated on the same C-8 HPLC column eluted with 45% MeCN/55% 0.1% aq. TFA to yield seven fractions, F7b-d7-8a to F7b-d7-8g, which yielded three pure compounds: microginin KR801 (**2**) (F7b-d7-8d, *R*_t_ 63.6 min, 11.4 mg, 4.5 × 10^−4^% yield), microginin KR767 (**1**) (F7b-d7-8e, *R*_t_ 64.8 min, 8.4 mg, 3.7 × 10^−4^% yield), and microginin KR835 (**3**) (F7b-d7-8g *R*_t_ 69.3 min, 7.6 mg, 3.3 × 10^−4^% yield).

Microginin KR767 (**1**): amorphous white material; ${\left[\alpha \right]}_{D}^{25}$ −26 (*c* 0.40, MeOH); UV (MeOH) λ_max_ (log ε) 202 (4.49), 224 (4.14), 278 (3.37) nm. For NMR data, see Table S2. HR ESI MS *m*/*z* 768.4551 ([M + H]^+^, calcd. for C_41_H_62_N_5_O_9_ *m*/*z* 768.4548). Retention times of AA Marfey’s derivatives: l-Tyr, 55.8 min; l-*N*Me-Leu, 51.1 min; l-Pro, 39.9 min (d-Pro, 40.7 min). Retention times of (2*R*,3*R*) *N*Me-Ahda derivatives on the UPLC-MS: Ahda-d-DAA, 21.16 min and Ahda-l-DAA, 22.20 min.

Microginin KR801 (**2**): amorphous white material; ${\left[\alpha \right]}_{D}^{25}$ –34 (*c* 0.55, MeOH); UV (MeOH) λ_max_ (log ε) 202 (4.47), 223 (4.18), 278 (3.36) nm. For NMR data, see Table S3. HR ESI MS *m*/*z* 802.4166/804.4132 (3:1, [M + H]^+^, calcd. for C_41_H_61_^35^ClN_5_O_9_ *m*/*z* 802.4158). Retention times of AA Marfey’s derivatives: l-Tyr, 55.7 min; l-*N*Me-Leu, 51.0 min; l-Pro, 39.7 min (d-Pro 40.7 min). Retention times of (2*R*,3*R*) Cl-*N*Me-Ahda derivatives on the UPLC-MS: Ahda-d-DAA, 20.16 min and Ahda-l-DAA, 21.20 min.

Microginin KR835 (**3**): amorphous white material; ${\left[\alpha \right]}_{D}^{25}$ –35 (*c* 0.36, MeOH); UV (MeOH) λ_max_ (log ε) 202 (4.45), 223 (4.13), 278 (3.38) nm. For NMR data, see Table S4. HR ESI MS *m*/*z* 836.3765/838.3778/840.3823 (9:6:1, [M + H]^+^, calcd. for C_41_H_60_^35^Cl_2_N_5_O_9_ *m*/*z* 836.3763). Retention times of AA Marfey’s derivatives: l-Tyr, 55.3 min; l-*N*Me-Leu, 50.8 min; l-Pro, 39.5 min (d-Pro, 40.7 min). Retention times of (2*R*,3*R*) diCl-*N*Me-Ahda derivatives on the UPLC-MS: Ahda-d-DAA, 21.60 min and Ahda-l-DAA, 22.71 min.

Microginin KR787 (**4**): amorphous white material; ${\left[\alpha \right]}_{D}^{25}$ –39 (*c* 0.13, MeOH); UV (MeOH) λ_max_ (log ε) 202 (4.47), 223 (4.15), 278 (3.26) nm. For NMR data, see Table S5. HR ESI MS *m*/*z* 788.3994/790.4020 (3:1 [M + H]^+^, calcd. for C_40_H_59_^35^ClN_5_O_9_ *m*/*z* 788.4001). Retention times of AA Marfey’s derivatives: l-Tyr, 55.5 min; l-*N*Me-Leu, 50.9 min; L-Pro, 39.7 min (d-Pro, 40.7 min). Retention times of (2*R*,3*R*) Cl-*N*Me-Ahda derivatives on the UPLC-MS: Ahda-d-DAA, 18.75 min and Ahda-l-DAA, 21.08 min.

Microginin KR604 (**5**): amorphous white material; ${\left[\alpha \right]}_{D}^{25}$ −41 (*c* 0.11, MeOH); UV (MeOH) λ_max_ (log ε) 201 (4.28), 278 (2.98), 485 (2.12) nm; For NMR data, see Table S6; HR ESI MS *m*/*z* 603.3764 (MH^−^, calcd. for C_32_H_51_N_4_O_7_ *m*/*z* 603.3758). Retention times of AA Marfey’s derivatives: l-Tyr, 55.0 min; l-*N*Me-Leu, 50.8 min; l-Pro, 39.6 min (d-Pro, 40.7 min). Retention times of (2*R*,3*R*) *N*Me-Ahda derivatives on the UPLC-MS: Ahda-d-DAA, 20.85 min and Ahda-l-DAA, 22.19 min.

Microginin KR638 (**6**): amorphous white material; ${\left[\alpha \right]}_{D}^{25}$ −50 (*c* 0.08, MeOH); UV (MeOH) λ_max_ (log ε) 202 (4.41), 278 (3.10) nm. For NMR data, see Table S7. HR ESI MS *m*/*z* 639.3521/641.3527 (3:1, MH^+^, calcd. for C_32_H_52_^35^ClN_4_O_7_ *m*/*z* 639.3525). Retention times of AA Marfey’s derivatives: l-Tyr, 55.4 min; l-*N*Me-Leu, 50.6 min; l-Pro, 39.7 min (d-Pro, 40.7 min). Retention times of (2*R*,3*R*) Cl-*N*Me-Ahda derivatives on the UPLC-MS: Ahda-d-DAA, 20.30 min and Ahda-l-DAA, 21.63 min.

Microginin KR781 (**7**): amorphous white material; ${\left[\alpha \right]}_{D}^{25}$ −78 (*c* 0.06, MeOH); UV (MeOH) λ_max_ (log ε) 202 (4.42), 278 (3.23) nm. For NMR data, see Table S8. HR ESI MS *m*/*z* 780.4553 ([M − H]^−^, calcd. for C_42_H_62_N_5_O_9_ *m*/*z* 780.4548). Retention times of AA Marfey’s derivatives: l-Tyr, 55.5 min; l-*N*Me-Leu, 50.9 min; l-Pro, 39.7 min (d-Pro, 40.7 min). Retention times of (2*R*,3*R*) *N*Me-Ahda derivatives on the UPLC-MS: Ahda-d-DAA, 21.23 min and Ahda-l-DAA, 22.38 min.

Microginin KR815 (**8**): amorphous white material; ${\left[\alpha \right]}_{D}^{25}$ −32 (*c* 0.10, MeOH); UV (MeOH) λ_max_ (log ε) 202 (4.34), 224 (4.00), 278 (3.18) nm. For NMR data, see Table S9. HR ESI MS *m*/*z* 816.4318/818.4380 (3:1 MH^+^, calcd. for C_42_H_63_^35^ClN_5_O_9_ *m*/*z* 816.4314). Retention times of AA Marfey’s derivatives: l-Tyr, 55.5 min; l-*N*Me-Leu, 50.9 min; l-Pro, 39.7 min (d-Pro, 40.7 min).

Microginin FR3 (**9**): amorphous white material; ${\left[\alpha \right]}_{D}^{25}$ −53 (*c* 0.11, MeOH); UV (MeOH) λ_max_ (log ε) 201 (4.57), 224 (4.18), 278 (3.36) nm. For NMR data, see Table S10. HR ESI MS *m*/*z* 728.3874 (MH^+^, calcd. for C_37_H_54_N_5_O_10_ *m*/*z* 728.3871). Retention times of AA Marfey’s derivatives: l-Tyr, 55.5 min; l-Thr, 34.4 min (d-Thr, 37.4 min); l-Pro, 39.7 min (d-Pro, 40.7 min). Retention times of (2*S*,3*R*) Ahda derivatives on the UPLC-MS: Ahda-d-DAA, 19.19 min and Ahda-l-DAA, 22.10 min.

Microginin FR4 (**10**): amorphous white material; ${\left[\alpha \right]}_{D}^{25}$ −66 (*c* 0.10, MeOH); UV (MeOH) λ_max_ (log ε) 201 (4.61), 224 (4.22), 278 (3.42) nm. For NMR data, see Table S11. HR ESI MS *m*/*z* 742.4031 (MH^+^, calcd. for C_38_H_56_N_5_O_10_ *m*/*z* 742.4027. Retention times of AA Marfey’s derivatives: l-Tyr, 55.5 min; l-Thr, 34.4 min (d-Thr, 37.4 min); l-Pro, 39.7 min (d-Pro, 40.7 min). Retention times of (2*S*,3*R*) *N*Me-Ahda derivatives on the UPLC-MS: Ahda-d-DAA, 20.64 min and Ahda-l-DAA, 21.67 min.

### 3.4. Determination of the Absolute Configuration of the Amino Acids by Marfey’s Method

Compounds **1**–**10** (0.5 mg each) were hydrolyzed in 6 N HCl (1 mL). The reaction mixture was maintained in a sealed glass bomb at 104 °C for 18 h. The acid was removed in vacuo, and the residue was suspended in 250 μL of H_2_O. A solution of 1-fluoro-2,4-dinitrophenyl-5-l-alanine amide (FDAA) [14] in acetone (0.03 M, 20 μL per each amino acid in the peptide) and NaHCO_3_ (1 M, 20 μL per each amino acid) were added to the reaction vessel. The reaction mixture was stirred at 40 °C for 2.5 h in the dark. HCl (2 M, 10 μL per each amino acid) was added to the reaction vessel, and the solution was evaporated in vacuo. The FDAA-amino acid derivatives from the hydrolysate were dissolved in 1 mL CH_3_CN and compared with standard FDAA-amino acids by HPLC analysis: LiChroCART RP-18 column (5 μm, 250 mm × 4.6 mm), flow rate 1 mL/min, UV detection at 340 nm, linear gradient elution from 0.1% aq. TFA buffer (pH 3) to 6:4 MeCN:0.1% aq. TFA buffer (pH 3) within 60 min. The absolute configuration of each amino acid was determined by spiking the derivatized hydrolysates with a d,l-mixture of the standard derivatized amino acids. 

The absolute configurations of C-3 of the Ahda derivatives of compounds **1**–**10** were analyzed by the advanced Marfey’s method [15]. A 0.5-mg portion of each compound was hydrolyzed, as described above, divided into two portions and derivatized, one with l-FDAA and the other with d-FDAA. The two samples of l- and d-FDAA derivatives were analyzed by ESI LC MS. The analysis was performed on a Waters Acquity UPLC coupled with a UV detector (Waters Acquity-TUV detector) and mass spectrometer (Waters Xevo TQD) on a C18 (1.7 μm, 2.1 Å, ~100 mm) column (Waters). The mobile phase compositions were (A) 95:5 H_2_O/MeCN, 0.1% formic acid and (B) MeCN, 0.1% formic acid. The elution gradient was as follows: 1 min of 100% A, linear gradient to 40% B over 25 min, and hold for 4 min. Samples of 10 μL were injected, and the flow rate was 0.5 mL/min. The UV detector was set to 340 nm, and the mass spectrometer was operated in both negative and positive ion modes, scanning between 200 and 650 mass units. The interpretation of the data was conducted after the run on both positive and negative ion modes using Waters MassLynx software (v4.1, Waters, Milford, MA, USA).

### 3.5. Aminopeptidase M Inhibitory Assay

Aminopeptidase M inhibitory activity was determined according to the procedure described by Ishida et al. [10]. A reaction mixture composed of 20 μL of l-leucine-*p*-nitroanilide (2 mM in 0.1 M Tris-HCl buffer at pH 7.0), 50 μL of 0.1 M Tris-HCl buffer (pH 7.0) and 20 μL of test solution was added to each well of a 96-well microtiter plate. The plate was incubated at 37 °C for 5 min; thereafter, 10 μL of porcine kidney microsomal aminopeptidase M [EC Number 232-942-5, Sigma-Aldrich (St. Louis, MO, USA), 5.5 μL in 3.5 M (NH_4_)_2_SO_4_] was added to each well, and the absorbance at 405 nm was measured for 30 min. The microginin samples were dissolved in DMSO at a concentration of 1 mg/mL, and the IC_50_ values were determined by analysis of a series of dilutions (from 45.5 μM to 0.00069 μM). A sigmoidal curve of the enzyme inhibition versus the concentration of the inhibitor was observed that was fit to a 4-parameter logistic model [19].

## 4. Conclusions

In the current research, we isolated ten microginins produced by a *Microcystis* sp. collected from the Kishon Reservoir in Israel. These microginins appear to be encoded by two distinct gene clusters that yield different sequences and absolute configurations of the chiral centers of the Ahda moiety. Compounds **1** to **6** share the same sequence: (2*R*,3*R*)-Ahda-l-Tyr-l-*N*MeLeu-l-Pro-X (X=H or l-Tyr). Compounds **9** and **10** share a different sequence: (2*S*,3*R*)-Ahda-l-Thr-l-Pro-l-Tyr-l-Tyr. Furthermore, these linear peptides are additionally altered by *N*-methylation of Ahda-amine group and, in the case of **1**–**6**, by mono- or dichlorination of Ahda terminal methyl group. Although inhibitory activity depends on the sequences of the microginins [10] and Ahda-*N*-methylation, chlorination of the Ahda terminal methyl group did not influence the extent of the aminopeptidase M inhibition. The augmentation of the diversity of the microginins by halogenation is common to cyanobacteria metabolites, including other protease inhibitors, such as the aeruginosins and micropeptins that are produced by *Microcystis* spp. [1,20,21]. The reason for augmentation of the diversity of these groups of metabolites by mono-, di-, and tri-halogenation and hetero-halogenation is not clear. It might reflect ecological condition or stress, be a signal for managing the cyanobacteria colony, or function in quorum sensing. Our current research is aimed at clarifying this misunderstood phenomenon.

## Figures and Tables

**Table 1:** ^1^H NMR data (500 MHz) of microginins **1**–**10** in DMSO-*d*_6_.

Position	1	2	3	4	5	6	7	8	Position	9	10
Ahda 2	4.30	4.30	4.30	4.14	4.26	4.29	4.30	4.30	Ahda 2	4.10	4.18
2-OH	6.47	6.47	6.46	6.35	6.35	6.41	6.35	6.40	2-OH	6.61	6.82
3	3.27	3.27	3.27	3.27	3.14	3.24	3.22	3.24	3	3.22	3.30
3-NH_1/2/3_	8.35	8.36	8.35	7.83	8.00	7.24	8.42	8.42	3-*N*H_2/3_	7.71	8.17
	8.49	8.49	8.48								8.36
3-*N*CH_3_	2.56	2.56	2.56	-	2.51	2.55	2.53	2.55	3-*N*CH_3_	-	2.50
4	1.39	1.39	1.40	1.37	1.34	1.41	1.36	1.39	4	1.59	1.58
	1.33	1.32	1.33	1.22	1.28	1.33	1.32	1.31		1.44	1.55
5	1.25	1.23	1.24	1.26	1.27	1.38	1.24	1.24	5	1.34	1.33
	1.13	1.11	1.14	1.12	1.13	1.14	1.13	1.13		1.29	1.30
6	1.18	1.13	1.22	1.13	1.14	1.15	1.17	1.13	6	1.23	1.23
	1.13		1.15				1.13				
7	1.19	1.18	1.24	1.22	1.19	1.22	1.20	1.22	7	1.23	1.23
8	1.20	1.33	1.43	1.34	1.20	1.34	1.20	1.33	8	1.23	1.23
9	1.24	1.68	2.13	1.68	1.26	1.68	1.25	1.67	9	1.24	1.24
10	0.84	3.60	6.29	3.60	0.84	3.60	0.84	3.60	10	0.85	0.85
^1^Tyr 2	4.86	4.86	4.86	4.84	4.87	4.86	4.86	4.86	Thr 2	4.48	4.47
2-NH	8.10	8.09	8.09	8.04	8.03	8.09	8.05	8.09	2-NH	7.86	7.91
3	2.86	2.86	2.86	2.88	2.87	2.87	2.87	2.85	3	3.94	3.93
	2.74	2.74	2.74	2.74	2.74	2.75	2.74	2.74			
5,5′	7.00	7.00	7.01	7.00	6.98	6.99	6.99	6.99	3-OH	5.13	5.10
6,6′	6.62	6.62	6.62	6.62	6.61	6.62	6.62	6.62	4	1.14	1.14
7-OH	9.25	9.26	9.26	9.24	9.24	9.25	9.25	9.25			
MeLue 2	5.23	5.23	5.23	5.23	5.24	5.24	5.23	5.23	Pro 2	4.32	4.32
2-*N*CH_3_	2.89	2.90	2.90	2.88	2.86	2.87	2.88	2.89	3	1.90	1.90
										1.69	1.70
3	1.48	1.45	1.46	1.45	1.53	1.53	1.47	1.47	4	1.74	1.74
	1.42	1.42	1.42	1.42	1.36	1.38	1.42	1.41		1.62	1.65
4	1.37	1.35	1.37	1.37	1.39	1.39	1.38	1.38	5	3.63	3.64
5	0.81	0.81	0.81	0.80	0.82	0.82	0.81	0.81	^1^Tyr 2	4.38	4.38
6	0.85	0.85	0.85	0.85	0.85	0.86	0.85	0.85	2-NH	7.64	7.64
Pro 2	4.31	4.31	4.32	4.30	4.17	4.18	4.29	4.29	3	2.87	2.87
										2.62	2.63
3	1.94	1.95	1.94	1.94	2.10	2.11	1.95	1.97	5,5′	7.00	7.00
	1.79	1.79	1.79	1.81	1.82	1.82	1.73	1.74			
4	1.80	1.76	1.80	1.81	1.86	1.86	1.80	1.78	6,6′	6.61	6.60
	1.73	1.73	1.73	1.76	1.81	1.81	1.75	1.73			
5	3.40	3.40	3.40	3.40	3.40	3.41	3.39	3.40	7-OH	9.13	9.12
	3.18	3.18	3.19	3.18	3.24	3.26	3.17	3.18			
^2^Tyr 2	4.27	4.27	4.26	4.27	-	-	4.31	4.30	^2^Tyr 2	4.31	4.31
2-NH	7.92	7.92	7.92	7.93	-	-	8.15	8.15	2-NH	8.03	8.04
3	2.86	2.86	2.87	2.88	-	-	2.86	2.86	3	2.89	2.90
	2.79	2.78	2.78	2.78			2.82	2.82		2.79	2.79
5,5′	7.01	7.01	7.00	6.99	-	-	7.00	7.00	5,5′	7.00	7.00
6,6′	6.64	6.64	6.64	6.62	-	-	6.64	6.64	6,6′	6.63	6.63
7-OH	9.20	9.21	9.20	9.19	-	-	9.23	9.23	7-OH	9.18	9.17
OCH_3_	-	-	-	-	-	-	3.53	3.53			

**Table 2:** ^13^C NMR data (125 MHz) of microginins **1**–**10** in DMSO-*d*_6_.

Position	1	2	3	4	5	6	7	8	Position	9	10
Ahda 1	170.1	170.1	170.1	170.2	170.5	170.1	170.2	170.1	Ahda 1	170.8	171.2
2	68.5	68.4	68.5	70.9	68.7	68.4	68.5	68.4	2	69.6	68.2
3	60.3	60.3	60.2	53.4	60.5	60.2	60.3	60.3	3	53.1	60.1
3-*N*CH_3_	30.8	30.8	30.8	-	31.4	30.8	30.8	30.8	3-*N*CH_3_	-	31.3
4	26.1	26.0	26.0	26.8	25.4	26.1	26.1	26.0	4	28.8	27.9
5	25.2	25.1	25.0	25.0	24.2	25.2	25.3	25.1	5	24.8	25.1
6	29.2	29.0	28.9	29.0	28.6	29.1	29.2	29.0	6	28.5	28.6
7	28.6	28.2	27.8	28.2	29.2	28.2	28.6	28.2	7	28.9	28.9
8	31.4	26.3	25.4	26.4	31.3	26.4	31.4	26.4	8	31.3	31.4
9	22.2	32.2	43.0	32.2	22.2	32.2	22.3	32.2	9	22.2	22.2
10	14.1	45.4	75.0	45.6	14.1	45.5	14.1	45.5	10	14.1	14.1
^1^Tyr 1	171.0	171.0	171.0	171.2	171.0	171.0	171.0	171.0	Thr 1	168.6	168.5
2	50.6	50.6	50.6	50.7	50.4	50.5	50.6	50.6	2	56.0	56.2
3	36.4	36.4	36.4	36.3	36.6	36.5	36.4	36.4	3	67.0	67.0
4	126.1	126.9	126.9	127.0	126.9	126.9	126.9	126.9	4	19.3	19.5
5,5′	130.2	130.3	130.3	130.4	130.3	130.3	130.3	130.3	Pro 1	171.0	171.0
6,6′	115.1	115.1	115.1	115.2	115.1	115.0	115.2	115.2	2	59.5	59.5
7	156.2	156.2	156.2	156.2	156.2	156.0	156.2	156.2	3	29.0	29.1
*N*MeLeu 1	168.5	168.5	168.5	168.5	168.3	168.3	168.4	168.4	4	24.2	24.2
2	51.9	51.9	51.9	52.0	51.6	51.7	51.9	51.9	5	47.4	47.5
2-*N*CH_3_	30.2	30.2	30.2	30.2	30.1	30.2	30.2	30.2	^1^Tyr 1	171.0	171.0
3	37.2	37.1	37.2	37.2	37.2	37.2	37.2	37.1	2	54.1	54.2
4	24.2	24.3	24.2	24.3	24.2	24.2	24.3	24.2	3	36.6	36.7
5	22.4	22.3	22.4	22.3	22.4	22.5	22.4	22.4	4	127.8	127.9
6	23.0	23.1	23.1	23.2	22.8	22.9	23.0	23.1	5,5′	130.2	130.2
Pro 1	171.4	171.5	171.5	171.5	173.4	173.2	171.7	171.7	6,6′	114.9	115.0
2	59.4	59.4	59.4	59.4	58.8	58.8	59.3	59.3	7	155.9	155.9
3	29.1	29.1	29.1	29.1	28.8	28.8	29.2	29.1	^2^Tyr 1	172.9	172.9
4	24.3	24.2	24.3	24.1	24.6	24.6	24.2	24.3	2	53.9	54.0
5	46.7	46.7	46.7	46.7	46.5	46.5	46.7	46.7	3	36.2	36.2
^2^Tyr 1	173.1	173.1	173.1	173.1	-	-	172.2	172.2	4	127.4	127.4
2	54.0	54.0	54.0	54.1	-	-	54.3	54.2	5,5′	130.2	130.3
3	36.1	36.1	36.1	36.2	-	-	36.1	36.1	6,6′	115.1	115.1
4	127.5	127.5	127.6	127.6	-	-	127.2	127.2	7	156.1	156.1
5,5′	130.3	130.3	130.3	130.3	-	-	130.2	130.2			
6,6′	115.1	115.1	115.1	115.2	-	-	115.2	115.1			
7	156.1	156.1	156.1	156.1	-	-	156.2	156.2			
OCH_3_	-	-	-	-	-	-	51.9	51.8			

**Table 3:** Comparison of the chemical shifts of Ahda-C-2, H-2, C-3, and H-3 in DMSO-*d*_6_ of **1**–**10** and known microginins.

Compound	Ahda Derivative	Absolute Configuration	C-2	H-2	C-3	H-3
Microginin [7]	Ahda	(2*S*,3*R*)	69.5	4.04	52.9	3.21
Microginin 51-A [10]	Ahda	(2*S*,3*S*)	70.4	4.14	52.9	3.24
Microginin 51-B [10]	*N*Me-Ahda	(2*S*,3*S*)	68.1	4.28	59.8	3.22
Microginin 478 [10]	*N*Me-Ahda	(2*S*,3*S*)	68.3	4.18	60.0	3.20
Microginin 91-A [10]	Cl-Ahda	(2*R*,3*R*)	70.5	4.26	53.0	3.36
Microginin 91-C [10]	Ahda	(2*R*,3*R*)	71.0	4.18	52.8	3.27
Microginin 91-D [10]	Cl-Ahda	(2*R*,3*R*)	70.5	4.26	53.0	3.36
Microginin 91-E [10]	Cl_2_-Ahda	(2*R*,3*R*)	70.7	4.26	53.0	3.36
Microginin 299-A [8]	Cl-Ahda	(2*S*,3*S*)	70.6	4.23	53.0	3.37
Microginin 299-B [8]	Cl_2_-Ahda	(2*S*,3*S*)	70.6	4.25	52.9	3.37
Microginin 299-C [8]	Ahda	(2*S*,3*S*)	70.6	4.20	53.0	3.37
Microginin 299-D [8]	Cl_2_-Ahda	(2*S*,3*S*)	70.6	4.21	52.9	3.36
Microginin AL584 [17]	Cl-Ahda	(2*S*,3*S*)	71.3	4.19	53.3	3.30
Microginin KR767 (**1**)	*N*Me-Ahda	(2*R*,3*R*)	68.5	4.30	60.3	3.27
Microginin KR801 (**2**)	Cl-*N*Me-Ahda	(2*R*,3*R*)	68.4	4.30	60.3	3.27
Microginin KR835 (**3**)	Cl_2_-*N*Me-Ahda	(2*R*,3*R*)	68.5	4.30	60.2	3.27
Microginin KR787 (**4**)	Cl-Ahda	(2*R*,3*R*)	70.9	4.14	53.4	3.27
Microginin KR604 (**5**)	*N*Me-Ahda	(2*R*,3*R*)	68.7	4.26	60.5	3.14
Microginin KR638 (**6**)	Cl-*N*Me-Ahda	(2*R*,3*R*)	68.4	4.29	60.2	3.24
Microginin KR781 (**7**)	*N*Me-Ahda	(2*R*,3*R*)	68.5	4.30	60.3	3.22
Microginin KR815 (**8**)	Cl-*N*Me-Ahda	(2*R*,3*R*)	68.4	4.30	60.3	3.24
Microginin FR3 (**9**)	Ahda	(2*S*,3*R*)	69.4	4.10	53.1	3.22
Microginin FR4 (**10**)	*N*Me-Ahda	(2*S*,3*R*)	68.0	4.18	60.1	3.30

**Table 4:** Inhibition of aminopeptidase M by the isolated microginins.

Compound Name	IC_50_ in μM ± SD	Compound Name	IC_50_ in μM ± SD
Microginin KR767 (**1**)	0.5 ± 0.2	Microginin KR638 (**6**)	3.8 ± 0.3
Microginin KR801 (**2**)	0.1 ± 0.2	Microginin KR815 (**8**)	72.0 ± 0.1
Microginin KR835 (**3**)	0.4 ± 0.2	Microginin FR3 (**9**)	6.2 ± 0.03
Microginin KR787 (**4**)	5.7 ± 0.1	Microginin FR4 (**10**)	1.8 ± 0.04
Microginin KR604 (**5**)	7.5 ± 0.1	-	-

## Supplementary Materials

The following are available online at http://www.mdpi.com/1660-3397/16/3/78/s1. Table S1: Isolated Microginins, Tables S2–S11: the full NMR data of **1**–**10**. 1D (^1^H, ^13^C) and 2D NMR (HSQC, HMBC, COSY, TOCSY, ROESY) spectra and HR MS data of compounds **1**–**10**.
