# Supplementary material for: Microginins from a Microcystis sp. Bloom Material Collected from the Kishon Reservoir, Israel
Source: Mar Drugs. 2018 Mar 2;16(3):78. doi: 10.3390/md16030078 (PMC5867622; doi:10.3390/md16030078)
Supplement: Supplementary file 1 [file marinedrugs-16-00078-s001.pdf]

Microginins from a *Microcystis* sp. bloom material collected from the Kishon Reservoir, Israel

Anat Lodin-Friedman and Shmuel Carmeli *

Raymond and Beverly Sackler School of Chemistry and Faculty of Exact Sciences, Tel Aviv University, Ramat-Aviv Tel- Aviv 69978, Israel;
anatlodin@gmail.com (ALF), carmeli@post.tau.ac.il (SC)

Supplementary Material

Table of content

S4. **Table S1**. Isolated Microginins.
S6. **Table S2**. NMR Data (500/125 MHz) of Microginin KR767 (**1**) in DMSO-d_6
S8. ^{1}H NMR Spectrum of Microginin KR767 (**1**) in DMSO-d_6
S9. ^{13}C NMR Spectrum of Microginin KR767 (1) in DMSO-d_6
S10. HSQC Spectrum Microginin KR767 (**1**) in DMSO-d_6
S11. HMBC Spectrum of Microginin KR767 (**1**) in DMSO-d_6
S12. COSY Spectrum of Microginin KR767 (**1**) in DMSO-d_6
S13. TOCSY Spectrum of Microginin KR767 (**1**) in DMSO-d_6
S14. ROESY Spectrum of Microginin KR767 (**1**) in DMSO-d_6
S15. HR ESI MS data of Microginin KR767 (**1**)
S16. **Table S3**. NMR Data (500/125 MHz) of Microginin KR801 (**2**) in DMSO-d_6
S18. ^{1}H NMR Spectrum of Microginin KR801 (**2**) in DMSO-d_6
S19. ^{13}C NMR Spectrum of Microginin KR801 (**2**) in DMSO-d_6
S20. HSQC Spectrum Microginin KR801 (**2**) in DMSO-d_6
S21. HMBC Spectrum of Microginin KR801 (**2**) in DMSO-d_6
S22. COSY Spectrum of Microginin KR801 (**2**) in DMSO-d_6
S23. TOCSY Spectrum of Microginin KR801 (**2**) in DMSO-d_6
S24. ROESY Spectrum of Microginin KR801 (**2**) in DMSO-d_6
S25. HR ESI MS data of Microginin KR801 (**2**)
S26. **Table S4**. NMR Data (500/125 MHz) of Microginin KR835 (**3**) in DMSO-d_6
S28. ^{1}H NMR Spectrum of Microginin KR835 (**3**) in DMSO-d_6
S29. ^{13}C NMR Spectrum of Microginin KR835 (**3**) in DMSO-d_6
S30. HSQC Spectrum Microginin KR835 (**3**) in DMSO-d_6
S31. HMBC Spectrum of Microginin KR835 (**3**) in DMSO-d_6
S32. COSY Spectrum of Microginin KR835 (**3**) in DMSO-d_6
S33. TOCSY Spectrum of Microginin KR835 (**3**) in DMSO-d_6
S34. ROESY Spectrum of Microginin KR835 (**3**) in DMSO-d_6
S35. HR ESI MS data of Microginin KR835 (**3**)
S36. **Table S5**. NMR Data (500/125 MHz) of Microginin KR787 (**4**) in DMSO-d_6
S38. ^{1}H NMR Spectrum of Microginin KR787 (**4**) in DMSO-d_6

S39. ^{13}C NMR Spectrum of Microginin KR787 (**4**) in DMSO-d_6
S40. HSQC Spectrum Microginin KR787 (**4**) in DMSO-d_6
S41. HMBC Spectrum of Microginin KR787 (**4**) in DMSO-d_6
S42. COSY Spectrum of Microginin KR787 (**4**) in DMSO-d_6
S43. TOCSY Spectrum of Microginin KR787 (**4**) in DMSO-d_6
S44. ROESY Spectrum of Microginin KR787 (**4**) in DMSO-d_6
S45. HR ESI MS data of Microginin KR787 (**4**)
S46. **Table S6**. NMR Data (500/125 MHz) of Microginin KR604 (**5**) in DMSO-d_6
S48. ^{1}H NMR Spectrum of Microginin KR604 (**5**) in DMSO-d_6
S49. ^{13}C NMR Spectrum of Microginin KR604 (**5**) in DMSO-d_6
S50. HSQC Spectrum Microginin KR604 (**5**) in DMSO-d_6
S51. HMBC Spectrum of Microginin KR604 (**5**) in DMSO-d_6
S52. COSY Spectrum of Microginin KR604 (**5**) in DMSO-d_6
S53. TOCSY Spectrum of Microginin KR604 (**5**) in DMSO-d_6
S54. ROESY Spectrum of Microginin KR604 (**5**) in DMSO-d_6
S55. HR ESI MS data of Microginin KR604 (**5**)
S56. **Table S7**. NMR Data (500/125 MHz) of Microginin KR638 (**6**) in DMSO-d_6
S58. ^{1}H NMR Spectrum of Microginin KR638 (**6**) in DMSO-d_6
S59. ^{13}C NMR Spectrum of Microginin KR638 (**6**) in DMSO-d_6
S60. HSQC Spectrum Microginin KR638 (**6**) in DMSO-d_6
S61. HMBC Spectrum of Microginin KR638 (**6**) in DMSO-d_6
S62. COSY Spectrum of Microginin KR638 (**6**) in DMSO-d_6
S63. TOCSY Spectrum of Microginin KR638 (**6**) in DMSO-d_6
S64. ROESY Spectrum of Microginin KR638 (**6**) in DMSO-d_6
S65. HR ESI MS data of Microginin KR638 (**6**)
S66. **Table S8**. NMR Data (500/125 MHz) of Microginin KR781 (**7**) in DMSO-d_6
S68. ^{1}H NMR Spectrum of Microginin KR781 (**7**) in DMSO-d_6
S69. ^{13}C NMR Spectrum of Microginin KR781 (**7**) in DMSO-d_6
S70. HSQC Spectrum Microginin KR781 (**7**) in DMSO-d_6
S71. HMBC Spectrum of Microginin KR781 (**7**) in DMSO-d_6
S72. COSY Spectrum of Microginin KR781 (**7**) in DMSO-d_6

S73. TOCSY Spectrum of Microginin KR781 (**7**) in DMSO-d_6
S74. ROESY Spectrum of Microginin KR781 (**7**) in DMSO-d_6
S75. HR ESI MS data of Microginin KR781 (**7**)
S76. **Table S9**. NMR Data (500/125 MHz) of Microginin KR815 (**8**) in DMSO-d_6
S78. ^{1}H NMR Spectrum of Microginin KR815 (**8**) in DMSO-d_6
S79. ^{13}C NMR Spectrum of Microginin KR815 (**8**) in DMSO-d_6
S80. HSQC Spectrum Microginin KR815 (**8**) in DMSO-d_6
S81. HMBC Spectrum of Microginin KR815 (**8**) in DMSO-d_6
S82. COSY Spectrum of Microginin KR815 (**8**) in DMSO-d_6
S83. TOCSY Spectrum of Microginin KR815 (**8**) in DMSO-d_6
S84. ROESY Spectrum of Microginin KR815 (**8**) in DMSO-d_6
S85. HR ESI MS data of Microginin KR815 (**8**)
S86. **Table S10**. NMR Data (500/125 MHz) of Microginin FR3 (**9**) in DMSO-d_6
S88. ^{1}H NMR Spectrum of Microginin FR3 (**9**) in DMSO-d_6
S89. ^{13}C NMR Spectrum of Microginin FR3 (**9**) in DMSO-d_6
S90. HSQC Spectrum Microginin FR3 (**9**) in DMSO-d_6
S91. HMBC Spectrum of Microginin FR3 (**9**) in DMSO-d_6
S92. COSY Spectrum of Microginin FR3 (**9**) in DMSO-d_6
S93. TOCSY Spectrum of Microginin FR3 (**9**) in DMSO-d_6
S94. ROESY Spectrum of Microginin FR3 (**9**) in DMSO-d_6
S95. HR ESI MS data of Microginin FR3 (**9**)
S96. **Table S11**. NMR Data (500/125 MHz) of Microginin FR4 (**10**) in DMSO-d_6
S98. ^{1}H NMR Spectrum of Microginin FR4 (**10**) in DMSO-d_6
S99. ^{13}C NMR Spectrum of Microginin FR4 (**10**) in DMSO-d_6
S100. HSQC Spectrum Microginin FR4 (**10**) in DMSO-d_6
S101. HMBC Spectrum of Microginin FR4 (**10**) in DMSO-d_6
S102. COSY Spectrum of Microginin FR4 (**10**) in DMSO-d_6
S103. TOCSY Spectrum of Microginin FR4 (**10**) in DMSO-d_6
S104. ROESY Spectrum of Microginin FR4 (**10**) in DMSO-d_6
S105. HR ESI MS data of Microginin FR4 (**10**)

S4. **Table S1**. Isolated Microginins.

Name	Residue 1[a]	2	3	4	5	6	Activity
Microginin[1]	(2*S*,3*R*)-Ahda	L-Ala	L-Val	*N*Me-L-Tyr	L-Tyr	-	ACE
Microginin 299-A[2]	Cl-(2*S*,3*S*)-Ahda	L-Val	*N*Me-L-Val	*N*Me-L-Tyr	L-Pro	L-Tyr	APM (5.2 uM), ACE-ni
Microginin 299-B[2]	Cl_2-(2*S*,3*S*)-Ahda	L-Val	*N*Me-L-Val	*N*Me-L-Tyr	L-Pro	L-Tyr	APM (7.1 uM), ACE-ni
Microginin FR1[3]	(2*S*,3*R*)-Ahda	L-Ala	L-Leu	*N*Me-L-Tyr	L-Tyr		ACE (16 uM), cAPM (1.3 uM), mAPM (6 nM)
Microginin 299-C[4]	(2*S*,3*S*)-Ahda	L-Val	*N*Me-L-Val	*N*Me-L-Tyr	L-Pro	L-Tyr	APM (2.3 uM), ACE-ni
Microginin 299-D[4]	Cl_2-(2*S*,3*S*)-Ahda	L-Val	*N*Me-L-Val	*N*Me-L-Tyr	L-Pro		APM (8.5 uM), ACE-ni
Microginin 99-A[4]	Cl-(3*R*)-Ada	L-Tyr	L-Leu	*N*Me-L-Tyr	L-Pro		APM, ACE-ni
Microginin 99-B[4]	Cl_2-(3*R*)-Ada	L-Tyr	L-Leu	*N*Me-L-Tyr	L-Pro		APM, ACE-ni
Microginin T1[5]	Cl-Ahda	L-Ala	L-Pro	L-Tyr	L-Tyr		ACE (6.8 uM), AMP (2.7 uM)
Microginin T2[5]	Ahda	L-Ala	L-Pro	L-Tyr	L-Tyr		ACE (10 uM), AMP (2.9 uM)
Microginin 478[6]	*N*Me-(2*S*,3*S*)-Ahda	L-Val	*N*Me-L-Val	*N*Me-L-Tyr	L-Tyr		APM (132 uM), ACE (13.2 uM)
Microginin 51-A[6]	(2*S*,3*S*)-Ahda	L-Tyr	*N*Me-L-Val	*N*Me-L-Tyr	L-Pro	L-Tyr	APM (4.9 uM), ACE-ni
Microginin 51-B[6]	*N*Me-(2*S*,3*S*)-Ahda	L-Tyr	*N*Me-L-Val	*N*Me-L-Tyr	L-Pro	L-Tyr	APM, ACE-ni
Microginin 91-A[6]	(2*R*,3*R*)-Ahda	L-Ile	*N*Me-L-Leu	L-Pro			APM, ACE-ni
Microginin 91-B[6]	Cl-(2*R*,3*R*)-Ahda	L-Ile	*N*Me-L-Leu	L-Pro			APM, ACE-ni
Microginin 91-C[6]	(2*R*,3*R*)-Ahda	L-Ile	*N*Me-L-Leu	L-Pro	L-Tyr		APM (71.1 uM), ACE-ni
Microginin 91-D[6]	Cl-(2*R*,3*R*)-Ahda	L-Ile	*N*Me-L-Leu	L-Pro	L-Tyr		APM (43.4 uM), ACE-ni
Microginin 91-E[6]	Cl_2-(2*R*,3*R*)-Ahda	L-Ile	*N*Me-L-Leu	L-Pro	L-Tyr		APM (19.5 uM), ACE-ni
Microginin SD755[7]	*N*Me-Ahoa	L-Val	*N*Me-L-Ile	*N*Me-L-Tyr	L-Tyr		APN (18.5 uM)
Nostoginin BN741[8]	(2*S*,3*S*)-Ahoa	L-Val	*N*Me-L-Ile	*N*Me-L-Tyr	L-Tyr		APN (1.3 uM)
Nostoginin BN578[8]	(2*S*,3*S*)-Ahoa	L-Val	*N*Me-L-Ile	*N*Me-L-Tyr	-		ND
Cyanostatin A[9]	(2*S*,3*R*)-Ahda	L-Ala	L-Val	*N*Me-L-Tyr	L-Hty		APM (55 uM), ACE (150 uM)
Cyanostatin B[9]	(2*S*,3*R*)-Ahda	L-Tyr	*N*Me-L-Leu	L-Pro	L-Tyr		APM (16 uM), ACE (170 uM)
Microginin AL584[10]	Cl-(2*S*,3*S*)-Ahda	L-Ala	*N*Me-L-Val	L-Tyr			APN-ni
Microginin HG787[11]	Cl-Ahda	L-Tyr	*N*Me-L-Ile	L-Pro	L-Tyr		APN (7.7 uM)
Microginin 680[12]	(2*S**,3*R**)-Cl_2-Ahoa	L-Tyr	*N*Me-L-Tyr	L-Pro			
Microginin 646[12]	(2*S**,3*R**)-Cl-Ahoa	L-Tyr	*N*Me-L-Tyr	L-Pro			
Microginin 612[12]	(2*S**,3*R**)-Ahoa	L-Tyr	*N*Me-L-Tyr	L-Pro			

Microginin 565[13]	Ahda	Ala	*N*Me-Leu	Tyr			
Microginin KR767[14]	(2*R*,3*R*)-*N*Me-Ahda	L-Tyr	*N*Me-L-Leu	L-Pro	L-Tyr		APM (0.5 nM)
Microginin KR801[14]	(2*R*,3*R*)-*N*Me-Cl-Ahda	L-Tyr	*N*Me-L-Leu	L-Pro	L-Tyr		APM (0.1 nM)
Microginin KR835[14]	(2*R*,3*R*)-*N*Me-Cl_2-Ahda	L-Tyr	*N*Me-L-Leu	L-Pro	L-Tyr		APM (0.4 nM)
Microginin KR604[14]	(2*R*,3*R*)-*N*Me-Ahda	L-Tyr	*N*Me-L-Leu	L-Pro			APM (7.5 nM)
Microginin KR638[14]	(2*R*,3*R*)-*N*Me-Cl-Ahda	L-Tyr	*N*Me-L-Leu	L-Pro			APM (3.8 nM)
Microginin KR815[14]	(2*R*,3*R*)-*N*Me-Cl-Ahda	L-Tyr	*N*Me-L-Leu	L-Pro	L-Tyr-OMe		APM (72.0 nM) esterification product
Microginin KR781[14]	(2*R*,3*R*)-*N*Me-Ahda	L-Tyr	*N*Me-L-Leu	L-Pro	L-Tyr-OMe		APM (? nM) esterification product
Microginin KR787[14]	(2*R*,3*R*)-Cl-Ahda	L-Tyr	*N*Me-L-Leu	L-Pro	L-Tyr		APM (5.7 nM)
Microginin FR3[14]	(2*S*,3*R*)-Ahda	L-Thr	L-Pro	L-Tyr	L-Tyr		APM (6.2 nM)
Microginin FR4[14]	(2*S*,3*R*)-*N*Me-Ahda	L-Thr	L-Pro	L-Tyr	L-Tyr		APM (1.8 nM)
Microginin 674[15]	(2*S*,3*S*)-Ahda	*N*Me-L-Met	L-Tyr	L-Tyr			ACE inhibitor
Microginin 690[16]	(2*S*,3*S*)-Ahda	*N*Me-L-Met(O)	L-Tyr	L-Tyr			ACE inhibitor oxidation product of 674

References

1. Okino, T.; Matsuda, H.; Murakami, M.; Yamaguchi, K. *Tetrahedron Lett.* **1993**, *34*, 501-504.
2. Ishida, K.; Matsuda, H.; Murakami, M.; Yamaguchi, K. *Tetrahedron* **1997**, *53*, 10281-10288.
3. Neumann, U.; Forchert, A.; Flury, T.; Weckesser, J. *FEMS Microbiol. Lett.* **1997**, *153*, 475-478.
4. Ishida, K.; Matsuda, H.; Murakami, M. *Tetrahedron* **1998**, *54*, 13475-13484.
5. Kodani, S.; Susuki, S.; Ishida, K.; Murakami, M. *FEMS Microbiol. Lett.* **1999**, *178*, 343-348.
6. Ishida, K.; Kato, T.; Murakami, M.: Watanabe, M.; Watanabe, M. F. *Tetrahedron* **2000**, *56*, 8643-8656.
7. Resef, V.; Carmeli, S. *Tetrahedron* **2001**, *57*, 2885-2894.
8. Ploutno, A.; Carmeli, S. *Tetrahedron* **2002**, *58*, 9949-9957.
9. Sano, T.; Takagi, H.; Morrison, L. F.; Metcalf, J. S.; Codd, G. A.; Kaya, K. *Phytochem.* **2005**, *66*, 543-548.
10. Gesner-Apter, S.; Carmeli, S. *Tetrahedron* **2008**, *64*, 6628-6634.
11. Lifshits, M.; Zafrir-Ilan, E.; Raveh, A.; Carmeli, S. *Tetrahedron* **2011**, *67*, 4017-4024.
12. Strangman, W. K., Wright, L. C. *Tet. Lett.* **2016**, *57*, 1801-1803.
13. Bagchi, S. N.; Sondhia, S.; Agrawal, M. K.; Banerjee, S. *J. Appl. Phycol.* **2016**, *28*, 177-180.
14. Lodin-Fridman, A, Carmeli, S. This manuscript.
15. Product of LKT Laboratories, Inc.
16. Product of Santa Cruz Biotechnology.

S6. **Table S2**. NMR Data (500/125 MHz) of Microginin KR767 (**1**) in DMSO-d_6.

Position	δ_C	δ_H Multiplicity, *J* (Hz)	HMBC correlations	COSY correlations	NOESY correlations
Ahda 1	170.1 C		Ahda-2,2-OH, [1]Tyr-2,NH		
2	68.5 CH	4.30 brs	Ahda-2-OH	Ahda-2-OH,3	Ahda-2-OH,3,3-NH_2, *N*CH_3,4,4',5',5', [1]Tyr-NH
2-OH		6.47 d, 6.1		Ahda-2	Ahda-2,3,3-*N*CH_3,4,6, [1]Tyr-NH
3	60.3 CH	3.27 brm	Ahda-2,2-OH,3-*N*CH_3	Ahda-2,3-NH_2,4,4'	Ahda-2,2-OH,3-NH_2, *N*CH_3,4,4',5',5', [1]Tyr-NH
3-NH_2		8.35 brs		Ahda-3,3-NH',3-*N*CH_3	Ahda-2,3,3-*N*CH_3
		8.49 brs		Ahda-3,3-NH',3-*N*CH_3	Ahda-2,3,3-*N*CH_3
3-*N*CH_3	30.8 CH_3	2.56 brt, 4.7		Ahda-3-NH_2	Ahda-2,2-OH,3,3-NH_2, 4,4'
4	26.1 CH_2	1.39 m	Ahda-2	Ahda-3,4',5,5'	Adha-2,3
		1.33 m		Ahda-3,4,5,5'	Adha-2,3
5	25.2 CH_2	1.25 m	Ahda-4,4'	Ahda-4,4',5',6	Adha-2,3
		1.13 m		Ahda-4,4',5,6	Adha-2,3
6	29.2 CH_2	1.18 m	Ahda-7,8		
		1.13 m		Ahda-7	
7	28.6 CH_2	1.19 m	Ahda-5,5',6,6'	Ahda-6'	
8	31.4 CH_2	1.20 m	Ahda-9,10	Ahda-9	
9	22.2 CH_2	1.24 m	Ahda-8,10	Ahda-8,10	
10	14.1 CH_3	0.84 t, 7.3	Ahda-9	Ahda-9	
[1]Tyr 1	171.0 C		[1]Tyr-2,3,3', *N*MeLeu-2, *N*CH_3		
2	50.6 CH	4.86 ddd, 8.2,7.7,6.3	[1]Tyr-3,3',NH	[1]Tyr-2-NH,3,3'	[1]Tyr-2-NH,3,3',5,5', *N*MeLeu-*N*Me
2-NH		8.10 d, 8.2		[1]Tyr-2	[1]Tyr-2,3,3',5,5', Ahda-2,2-OH,3
3	36.4 CH_2	2.86 m	[1]Tyr-2,5,5'	[1]Tyr-2,3'	[1]Tyr-2,3',5,5'
		2.74 m		[1]Tyr-2,3	[1]Tyr-2,3,5,5'
4	126.1 C		[1]Tyr-2,3,3',6,6'		
5,5'	130.2 CH	7.00 d, 8.4	[1]Tyr-3,3',5',5,6,6'	[1]Tyr-6,6'	[1]Tyr-2,3,3',6,6'

6,6’	115.1 CH	6.62 d, 8.4	[1]Tyr-5,5’,6’,6,7-OH	[1]Tyr-5,5’	[1]Tyr-5,5’,7-OH
7	156.2 C		[1]Tyr-5,5’,6,6’,7-OH		
7-OH		9.25 s			[1]Tyr-6,6’
*N*MeLeu 1	168.5 C		*N*MeLeu-2,3,3’		
2	51.9 CH	5.23 dd, 8.1,6.6	*N*MeLeu-3,3’,*N*Me	*N*MeLeu-3,3’	*N*MeLeu-3,3’,5,6,*N*Me, Pro-5,5’
2-*N*CH_3	30.2 CH_3	2.89 s	*N*MeLeu-2		*N*MeLeu-2,3,3’
3	37.2 CH_2	1.48 m	*N*MeLeu-2,4,5,6	*N*MeLeu-2,3’,4	*N*MeLeu-2,5,6,*N*Me Pro-5,5’
		1.42 m		*N*MeLeu-2,3,4	*N*MeLeu-2,5,6,*N*Me, Pro-5,5’
4	24.2 CH	1.37 m	*N*MeLeu-2,3,3’,5,6	*N*MeLeu-3,3’,5,6	*N*MeLeu-*N*Me
5	22.4 CH_3	0.81 d, 6.6	*N*MeLeu-3,3’,4,6	*N*MeLeu-4	*N*MeLeu-2,3,3’
6	23.0 CH_3	0.85 d, 6.6	*N*MeLeu-3,3’,4,5	*N*MeLeu-4	*N*MeLeu-2,3,3’
Pro 1	171.4 C		Pro-2,3’, [2]Tyr-NH		
2	59.4 CH	4.31 m	Pro-4’	Pro-3,3’	Pro-3,4,5’
3	29.1 CH_2	1.94 m	Pro-2,4,4’,5’	Pro-2,3’,4,4’	Pro-2,4’
		1.79 m		Pro-2,3,4,4’	
4	24.3 CH_2	1.80 m	Pro-2,3,5’	Pro-3,3’,5,5’	Pro-2
		1.73 m		Pro-3,3’,5,5’	Pro-3
5	46.7 CH_2	3.40 m	Pro-2	Pro-4,4’,5’	Pro-4,4’,5’, *N*MeLeu-2,3,3’
		3.18 dt, 9.0, 7.3		Pro-4,4’,5	Pro-2,3,4,4’,5, *N*MeLeu-2,3,3’
[2]Tyr 1	173.1 C		[2]Tyr-2,3,3’		
2	54.0 CH	4.27 td, 7.6,5.6	[2]Tyr-3,3’,NH	[2]Tyr-3,3’,NH	[2]Tyr-3,3’,5,5’,NH
2-NH		7.92 d, 7.6		[2]Tyr-2	[2]Tyr-2,3,3’,5,5’, Pro-3’
3	36.1 CH_2	2.86 m	[2]Tyr-2,NH,5,5’	[2]Tyr-2,3’	[2]Tyr-2,3’,5,5’,NH
		2.79 m		[2]Tyr-2,3	[2]Tyr-2,3,5,5’,NH
4	127.5 C		[2]Tyr-2,3,3’,6,6’		
5,5’	130.3 CH	7.01d, 8.4	[2]Tyr-3,3’,5’,5,6,6’	[2]Tyr-6,6’	[2]Tyr-2,3,3’,6,6’,NH
6,6’	115.1 CH	6.64 d, 8.4	[2]Tyr-5,5’,6’,6,7-OH	[2]Tyr-5,5’	[2]Tyr-5,5’,7-OH
7	156.1 C		[2]Tyr-5,5’,6,6’,7-OH		
7-OH		9.20 s			[2]Tyr-6,6’

S8. ^{1}H NMR Spectrum of Microginin KR767 (**1**) in DMSO-d_6

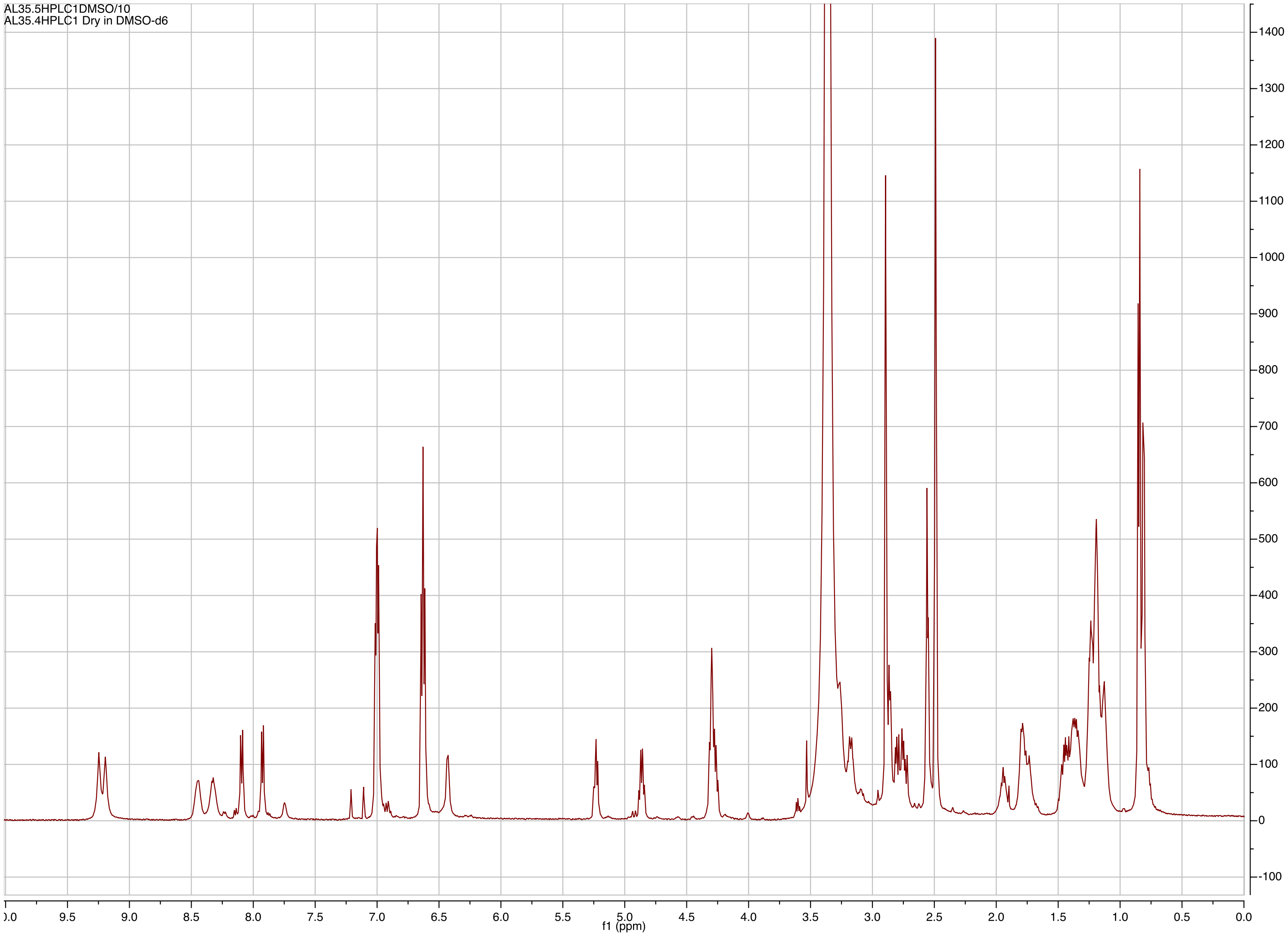

S9. ^{13}C NMR Spectrum of Microginin KR767 (**1**) in DMSO-d_6

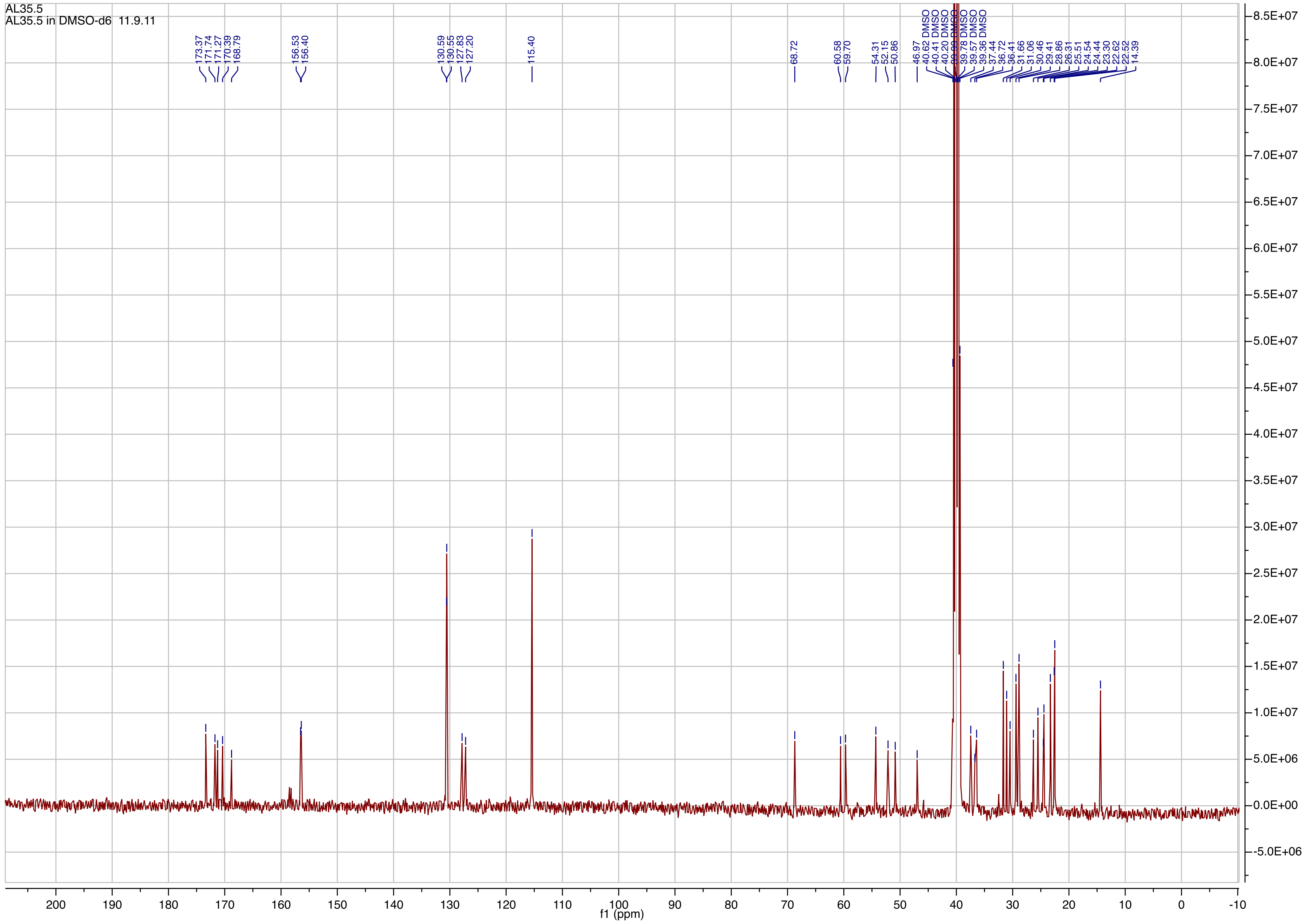

S10. HSQC Spectrum Microginin KR767 (**1**) in DMSO-d_6

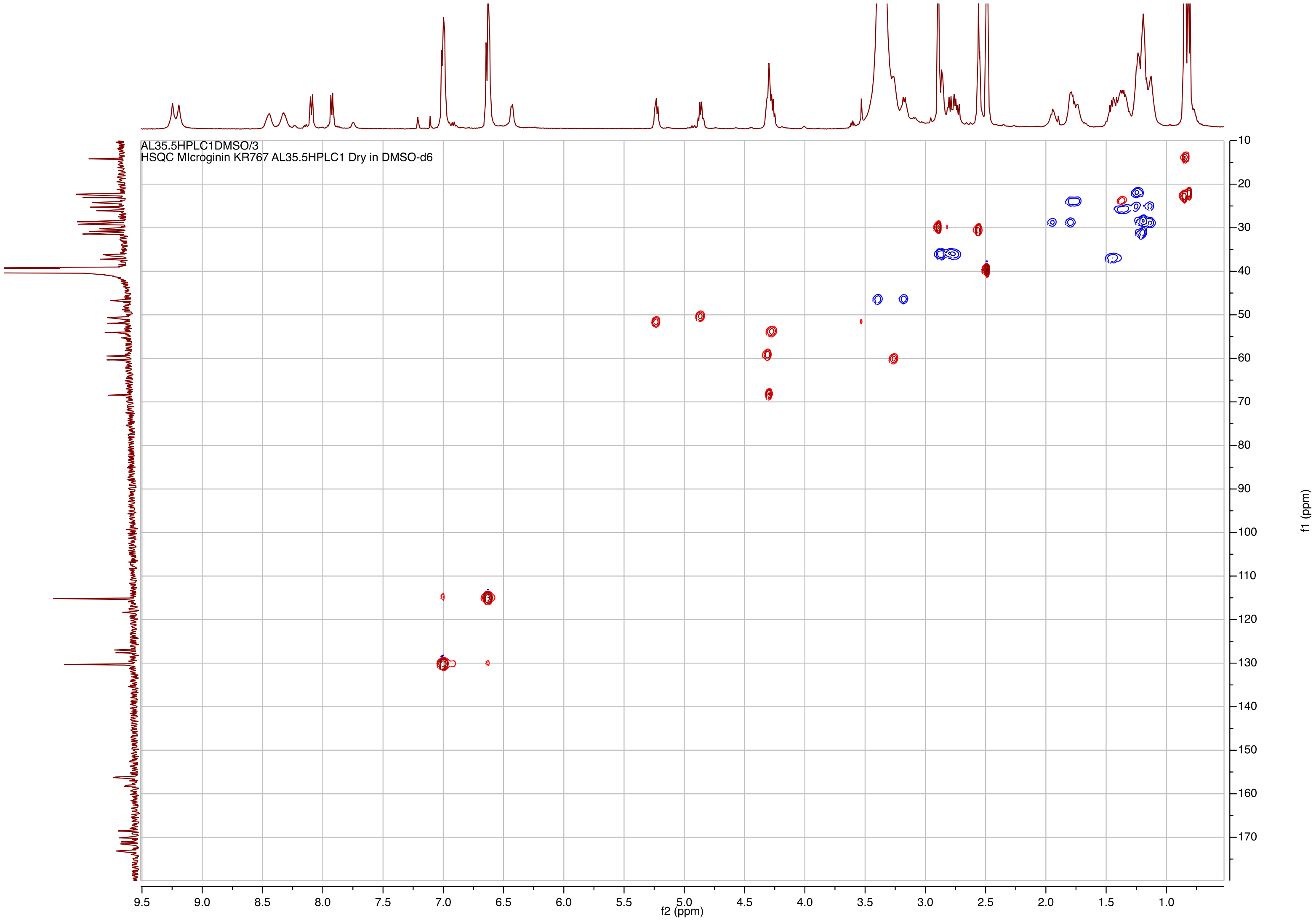

S11. HMBC Spectrum of Microginin KR767 (**1**) in DMSO-d_6

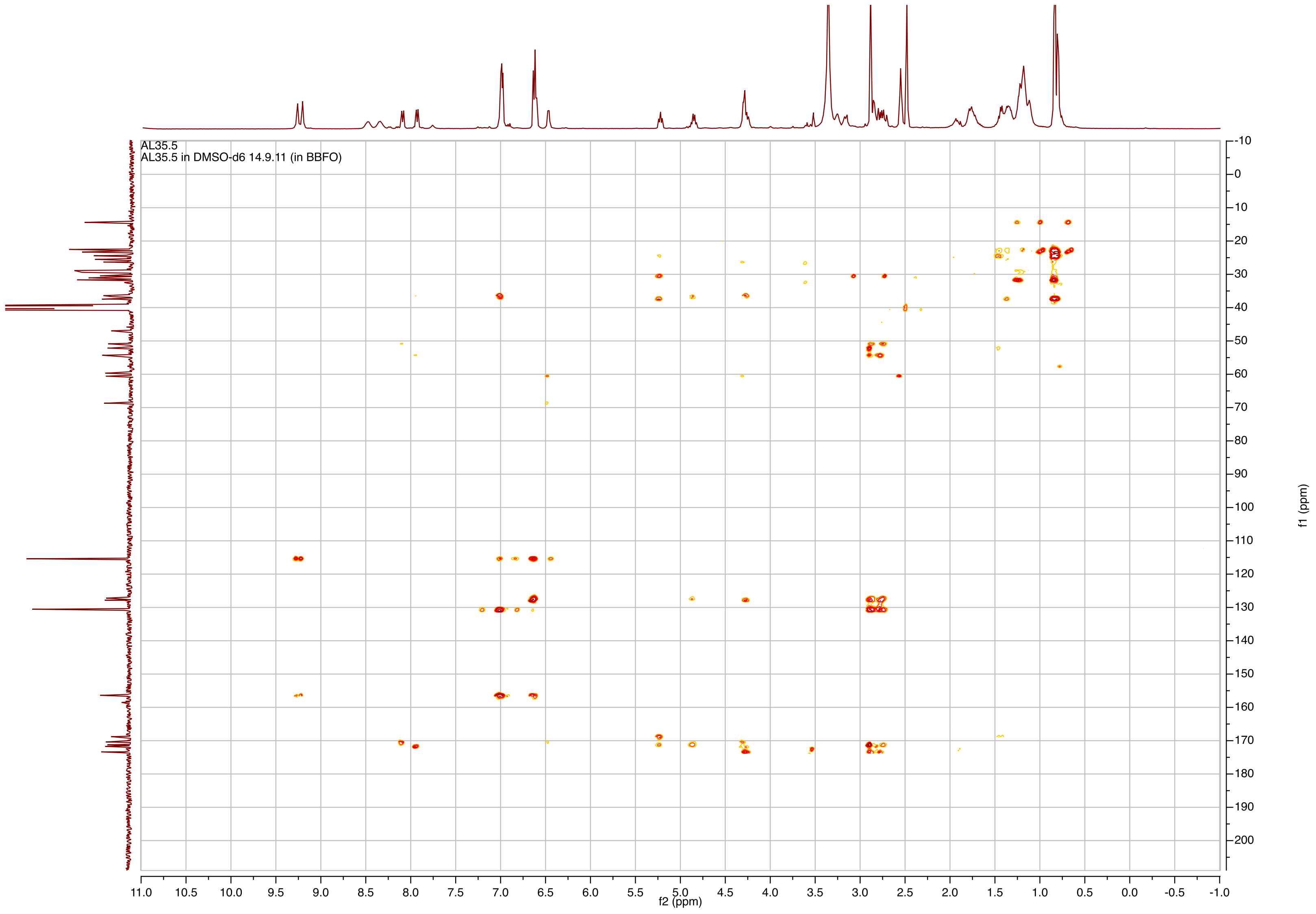

S12. COSY Spectrum of Microginin KR767 (**1**) in DMSO-d_6

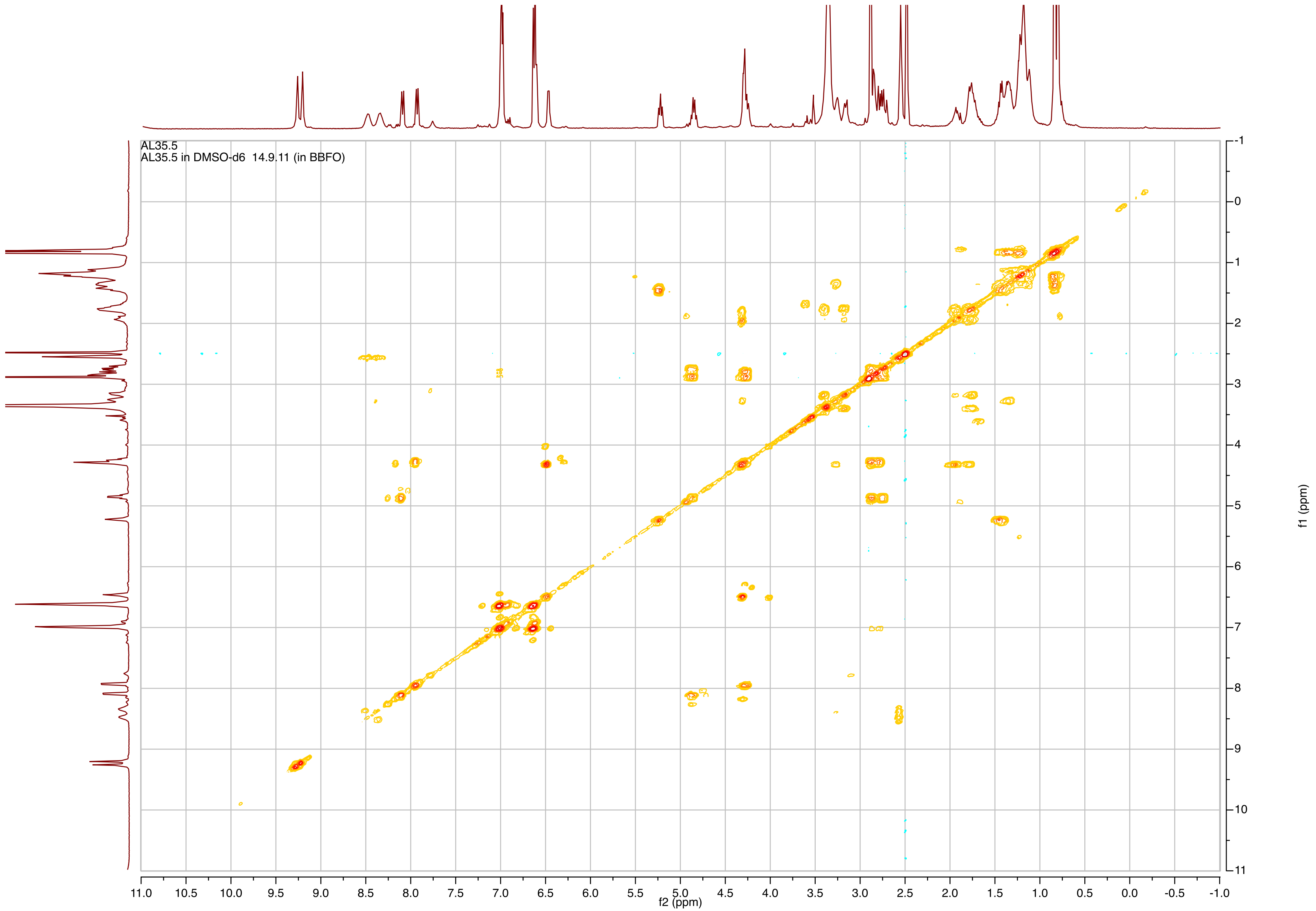

S13. TOCSY Spectrum of Microginin KR767 (**1**) in DMSO-d_6

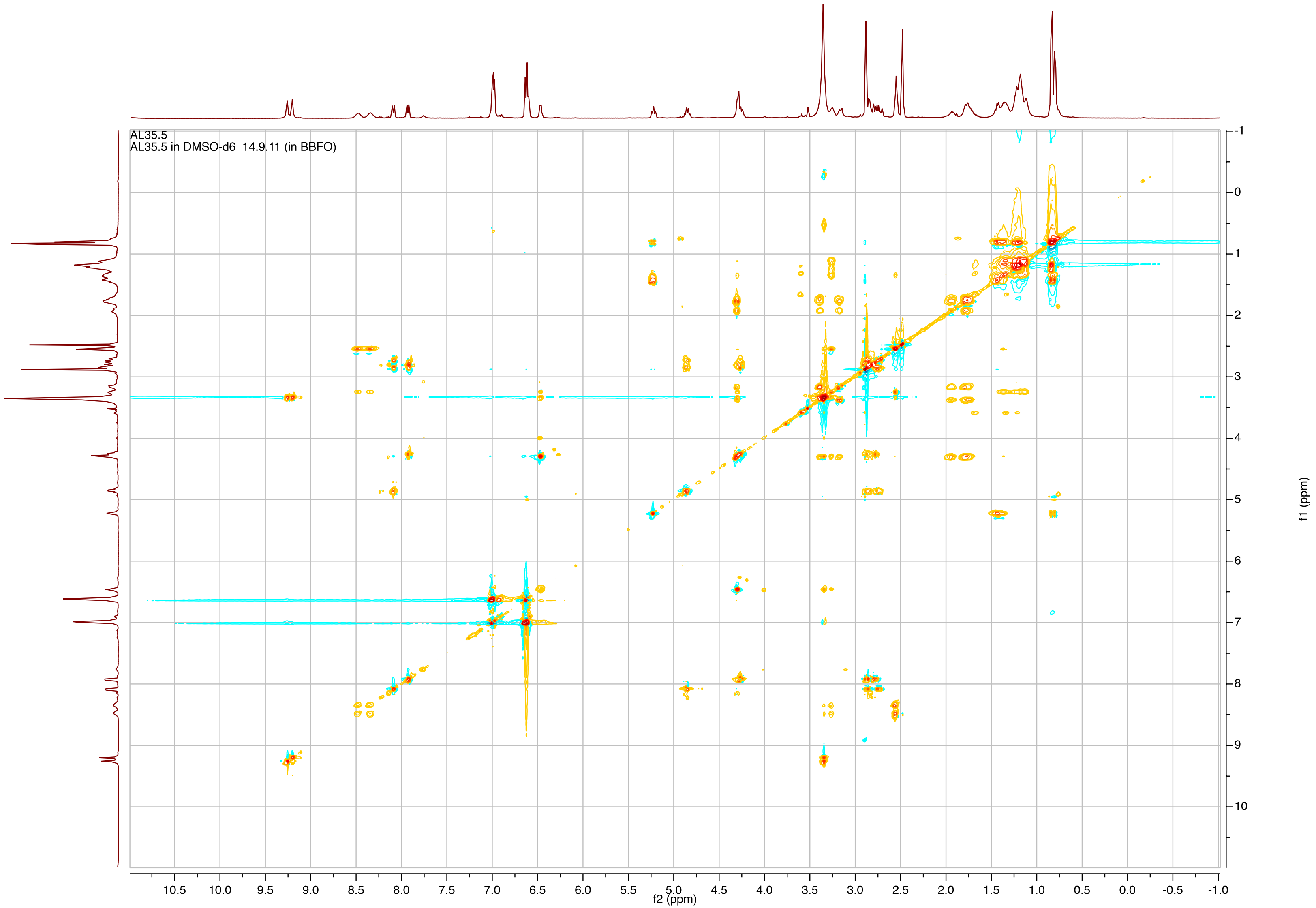

S14. ROESY Spectrum of Microginin KR767 (**1**) in DMSO-d_6

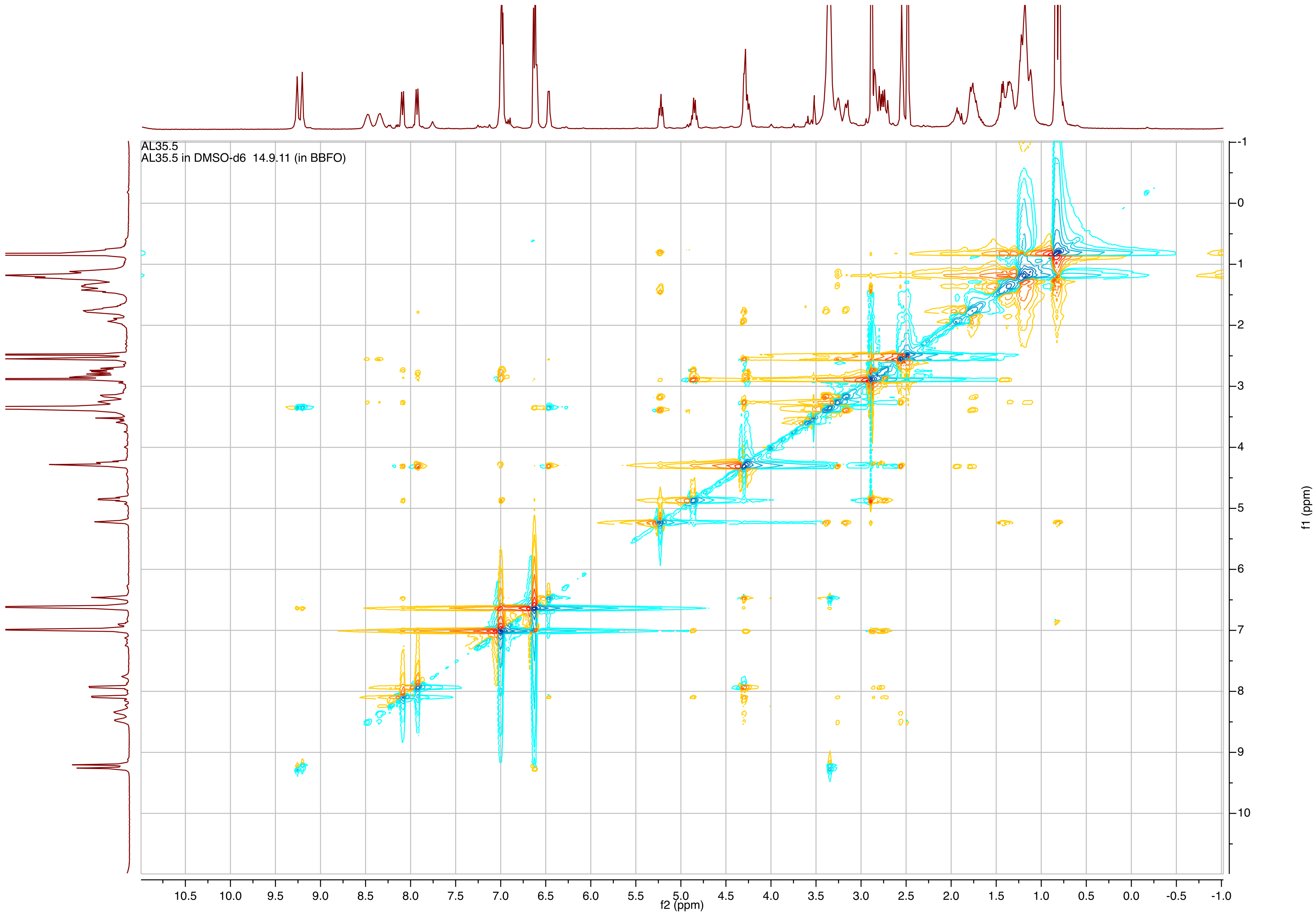

S15. HR ESI MS data of Microginin KR767 (**1**)

Elemental Composition Report

Single Mass Analysis

Tolerance = 2.0 PPM / DBE: min = -1.5, max = 50.0
Element prediction: Off
Number of isotope peaks used for i-FIT = 2

Monoisotopic Mass, Even Electron Ions
309 formula(e) evaluated with 3 results within limits (up to 50 closest results for each mass)
Elements Used:
C: 35-45 H: 55-65 N: 0-10 O: 5-15 Cl: 0-3

AL35.5
carmeli684 40 (1.770)
Anat Iodin
1: TOF MS ES+
5.00e+004

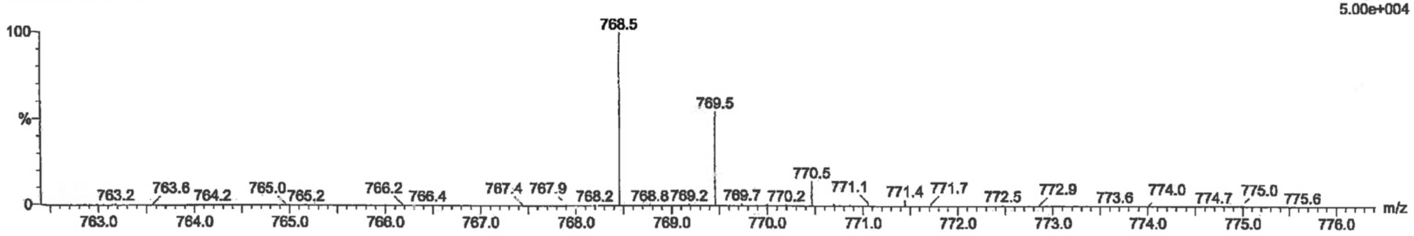

Minimum:				-1.5			
Maximum:		10.0	2.0	50.0			
Mass	Calc. Mass	mDa	PPM	DBE	i-FIT	i-FIT (Norm)	Formula
768.4551	768.4548	0.3	0.4	13.5	62.0	1.2	C41 H62 N5 O9 ←
	768.4561	-1.0	-1.3	18.5	61.2	0.4	C42 H58 N9 O5
	768.4539	1.2	1.6	9.5	64.4	3.6	C36 H63 N9 O7 Cl

S16. **Table S3**. NMR Data (500/125 MHz) of Microginin KR801 (**2**) in DMSO-d_6.

Position	δ_C	δ_H Multiplicity, *J* (Hz)	HMBC correlations	COSY correlations	NOESY correlations
Ahda 1	170.1 C		Ahda-2, [1]Tyr-2,NH		
2	68.4 CH	4.30 brs	Ahda-2-OH	Ahda-2-OH,3	Ahda-2-OH,3,3-NH_2,*N*CH_3,4,4',5',5',[1]Tyr-NH
2-OH		6.47 d, 5.6		Ahda-2	Ahda-2,3,3-*N*CH_3,4,4'5,[1]Tyr-NH
3	60.3 CH	3.27 brm	Ahda-2,4',3-*N*CH_3	Ahda-2,3-NH_2,4,4'	Ahda-2,2-OH,3-NH_2,*N*CH_3,4,4',5',5', [1]Tyr-NH
3-NH_2		8.36 brs		Ahda-3,3-NH',3-*N*CH_3	Ahda-2,3,3-*N*CH_3
		8.49 brs		Ahda-3,3-NH',3-*N*CH_3	Ahda-2,3,3-*N*CH_3
3-*N*CH_3	30.8 CH_3	2.56 brt, 4.8		Ahda-3-NH_2	Ahda-2,2-OH,3,3-NH_2, 4,4'
4	26.0 CH_2	1.39 m		Ahda-3,4',5,5'	Adha-2,3,2-OH
		1.32 m		Ahda-3,4,5,5'	Adha-2,3,2-OH
5	25.1 CH_2	1.23 m	Ahda-4,4',6,7	Ahda-4,4',5',6	Adha-2,3,2-OH
		1.11 m		Ahda-4,4',5,6	Adha-2,3
6	29.0 CH_2	1.13 m	Ahda-5,8	Ahda-7	
7	28.2 CH_2	1.18 m	Ahda-8,9	Ahda-6,8	
8	26.3 CH_2	1.33 m	Ahda-7,9,10	Ahda-7,9	
9	32.2 CH_2	1.68 tt, 7.3,6.6	Ahda-8,10	Ahda-8,10	
10	45.4 CH_2	3.60 t, 6.6	Ahda-8,9	Ahda-9	
[1]Tyr 1	171.0 C		[1]Tyr-2,3,3', *N*MeLeu-2, *N*CH_3		
2	50.6 CH	4.86 ddd, 8.5,7.7,6.2	[1]Tyr-3,3',NH	[1]Tyr-2-NH,3,3'	[1]Tyr-2-NH,3,3',5,5', *N*MeLeu-*N*Me
2-NH		8.09 d, 8.5		[1]Tyr-2	[1]Tyr-2,3,3',5,5', Ahda-2,2-OH,3
3	36.4 CH_2	2.86 m	[1]Tyr-2,5,5',NH	[1]Tyr-2,3'	[1]Tyr-2,3',5,5',NH
		2.74 m		[1]Tyr-2,3	[1]Tyr-2,3,5,5',NH
4	126.9 C		[1]Tyr-2,3,3',6,6'		
5,5'	130.3 CH	7.00 d, 8.1	[1]Tyr-3,3',5',5,6,6'	[1]Tyr-6,6'	[1]Tyr-2,3,3',6,6'
6,6'	115.1 CH	6.62 d, 8.1	[1]Tyr-5,5',6',6	[1]Tyr-5,5'	[1]Tyr-5,5',7-OH

7	156.2 C		[1]Tyr-5,5',6,6'		
7-OH		9.26 s			[1]Tyr-6,6'
*N*MeLeu 1	168.5 C		*N*MeLeu-2,3,3'		
2	51.9 CH	5.23 dd, 8.0,6.4	*N*MeLeu-3,3',4,*N*Me	*N*MeLeu-3,3'	*N*MeLeu-3,3',4,5,6,*N*Me, Pro-5,5'
2-*N*CH_3	30.2 CH_3	2.90 s	*N*MeLeu-2		*N*MeLeu-2,3,3',4
3	37.1 CH_2	1.45 m	*N*MeLeu-2,4,5,6	*N*MeLeu-2,3',4	*N*MeLeu-2,5,6,*N*Me Pro-5
		1.42 m		*N*MeLeu-2,3,4	*N*MeLeu-2,5,6,*N*Me, Pro-5
4	24.3 CH	1.35 m	*N*MeLeu-2,3,3',5,6	*N*MeLeu-3,3',5,6	*N*MeLeu-*N*Me
5	22.3 CH_3	0.81 d, 6.3	*N*MeLeu-3,3',4,6	*N*MeLeu-4	*N*MeLeu-2,3,3'
6	23.1 CH_3	0.85 d, 6.4	*N*MeLeu-3,3',4,5	*N*MeLeu-4	*N*MeLeu-2,3,3'
Pro 1	171.5 C		Pro-2,3,3', [2]Tyr-NH		
2	59.4 CH	4.31 m	Pro-3,3',4,4'	Pro-3,3'	Pro-3,3'
3	29.1 CH_2	1.95 m	Pro-2,4,5'	Pro-2,3',4,4',5,5'	Pro-2,4'
		1.79 m		Pro-2,3,4,4'	Pro-2
4	24.2 CH_2	1.76 m	Pro-2,3,3',5'	Pro-3,3',5,5'	Pro-2
		1.73 m		Pro-3,3',5,5'	Pro-3
5	46.7 CH_2	3.40 m	Pro-2,3',4,4'	Pro-3,4,4',5'	Pro-3,5', *N*MeLeu-2,3,3'
		3.18 dt, 9.0, 7.8		Pro-3,4,4',5	Pro-3,4,4',5, *N*MeLeu-2,*N*Me
[2]Tyr 1	173.1 C		[2]Tyr-2,3,3'		
2	54.0 CH	4.27 td, 7.6,5.6	[2]Tyr-3,3',NH	[2]Tyr-3,3',NH	[2]Tyr-3,3',5,5',NH
2-NH		7.92 d, 7.6		[2]Tyr-2	[2]Tyr-2,3,3', Pro-2,3'
3	36.1 CH_2	2.86 m	[2]Tyr-2,5,5',NH	[2]Tyr-2,3'	[2]Tyr-2,3',5,5',NH
		2.78 m		[2]Tyr-2,3	[2]Tyr-2,3,5,5',NH
4	127.5 C		[2]Tyr-2,3,3',6,6'		
5,5'	130.3 CH	7.01 d, 8.3	[2]Tyr-3,3',5',5,6,6'	[2]Tyr-6,6'	[2]Tyr-2,3,3',6,6',NH
6,6'	115.1 CH	6.64 d, 8.3	[2]Tyr-5,5',6',6	[2]Tyr-5,5'	[2]Tyr-5,5',7-OH
7	156.1 C		[2]Tyr-5,5',6,6'		
7-OH		9.21 s			[2]Tyr-6,6'

S18. ^{1}H NMR Spectrum of Microginin KR801 (**2**) in DMSO-d_6

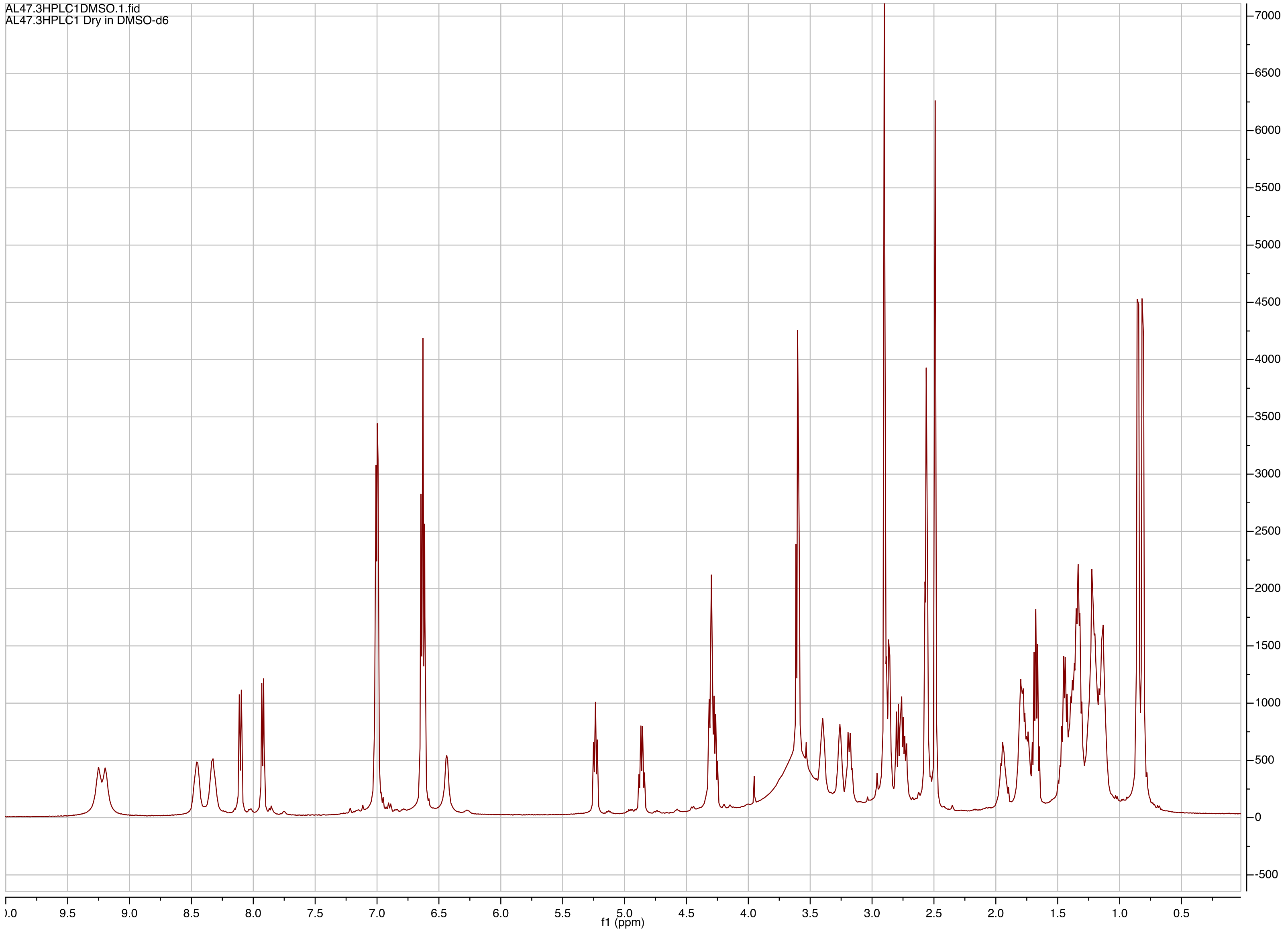

S19. ^{13}C NMR Spectrum of Microginin KR801 (**2**) in DMSO-d_6

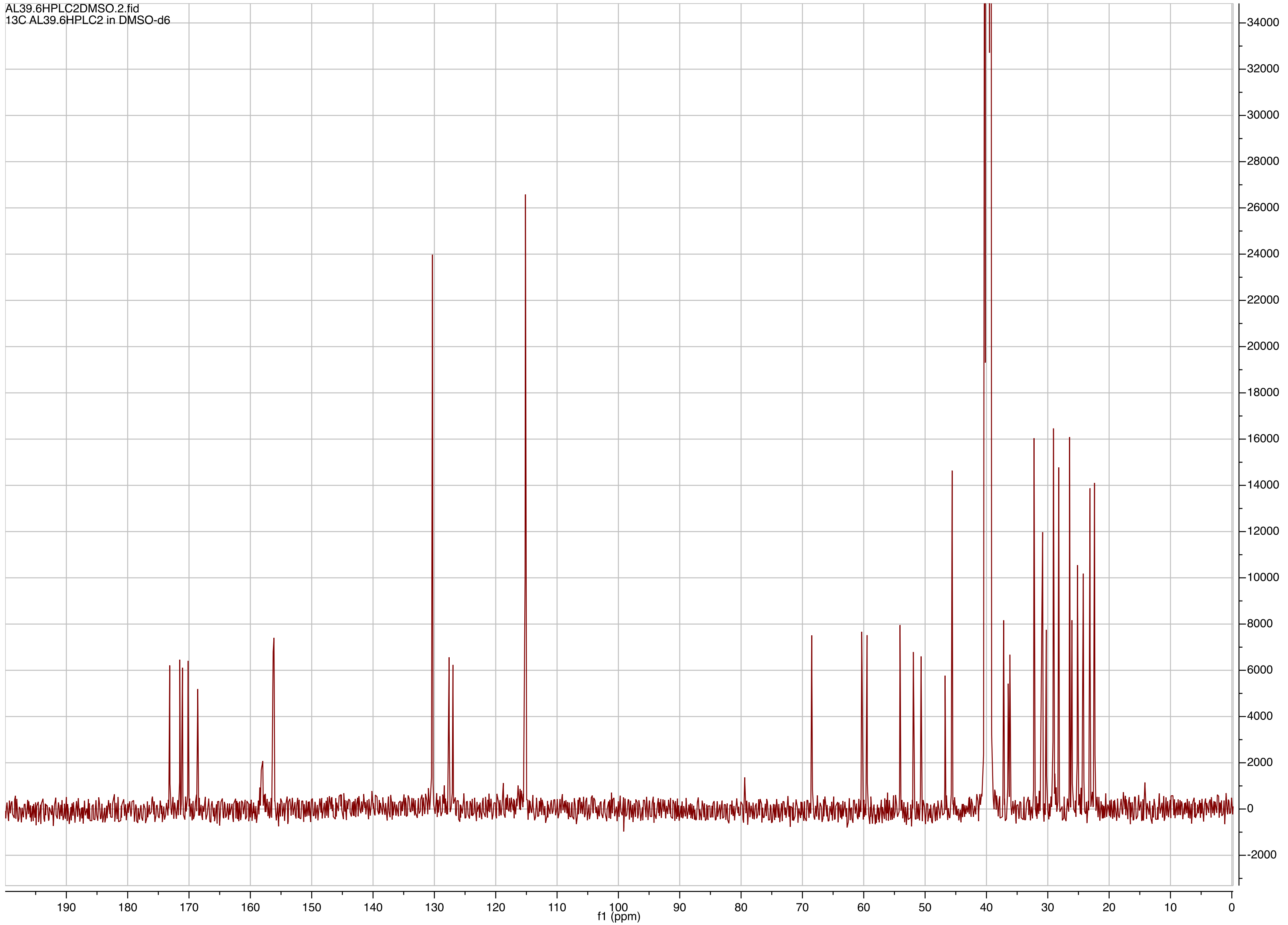

S20. HSQC Spectrum Microginin KR801 (**2**) in DMSO-d_6

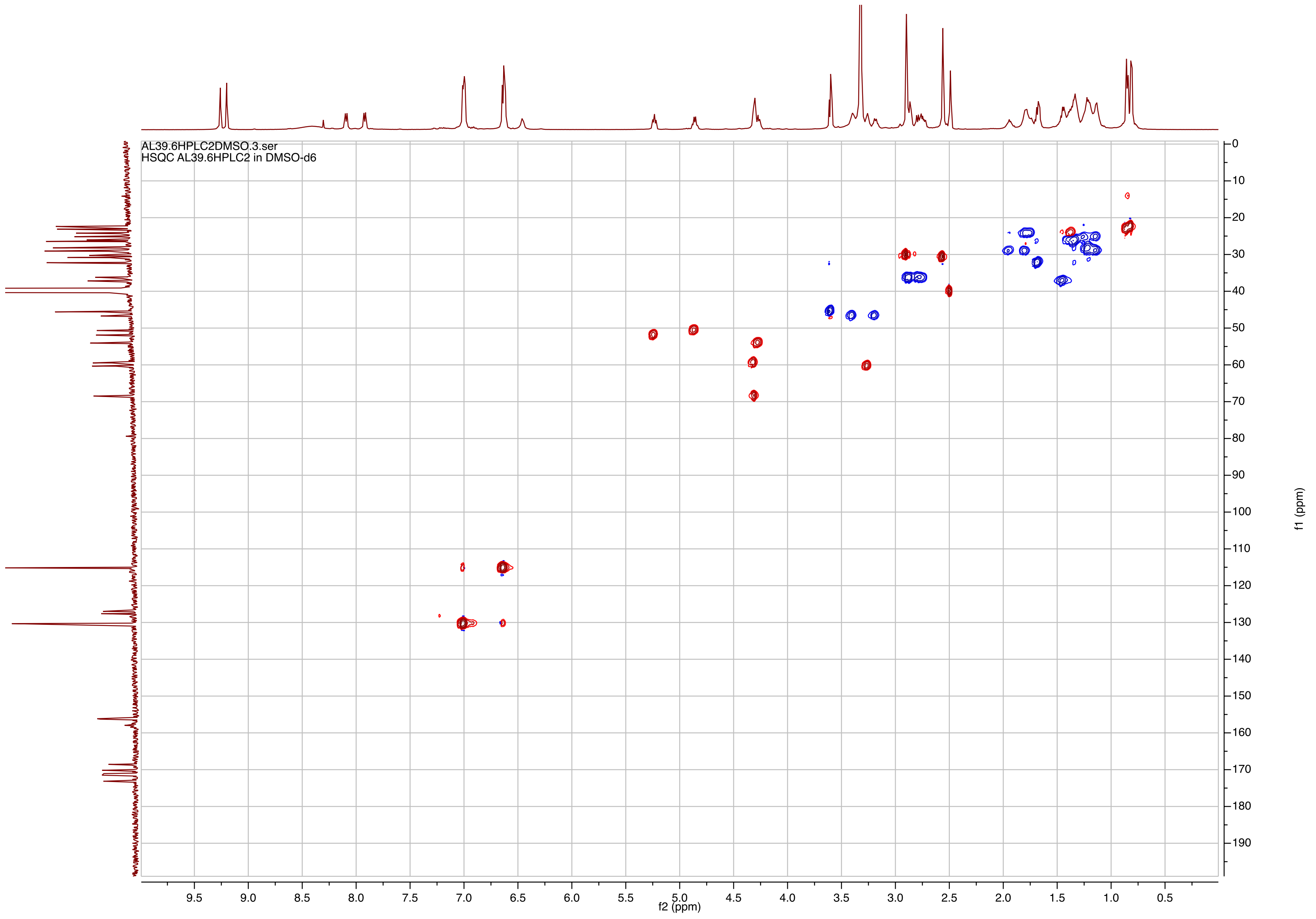

S21. HMBC Spectrum of Microginin KR801 (**2**) in DMSO-d_6

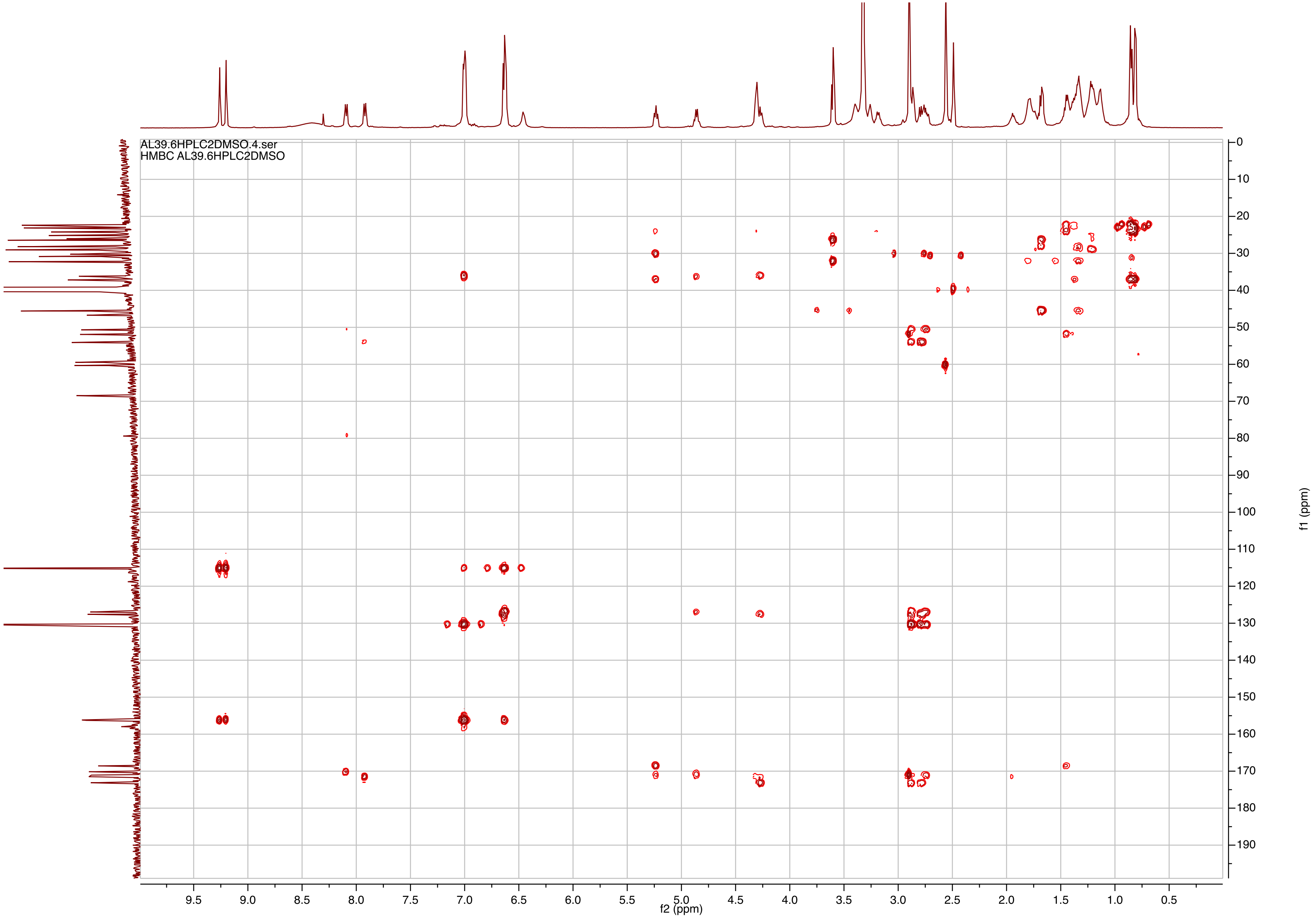

S22. COSY Spectrum of Microginin KR801 (**2**) in DMSO-d_6

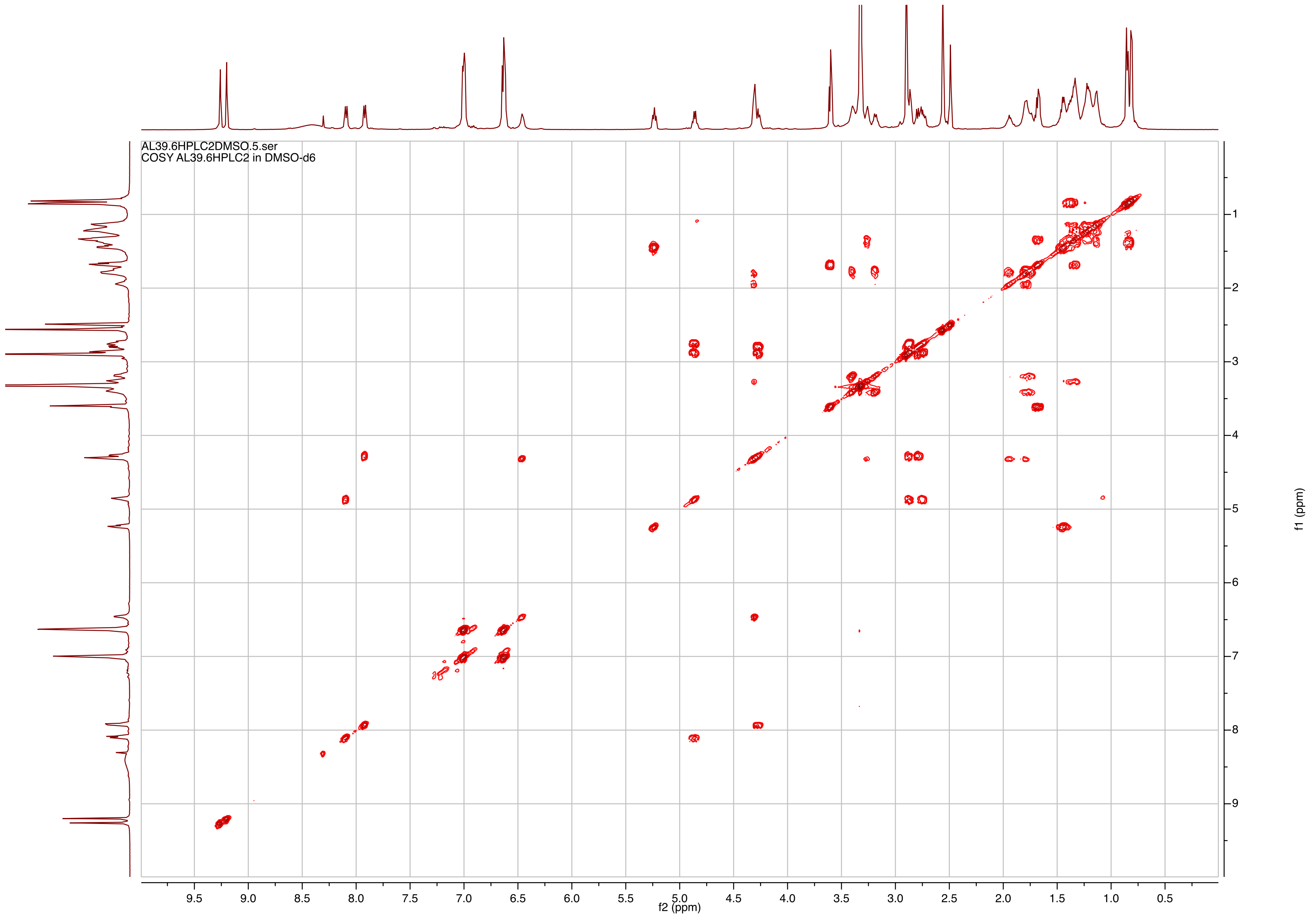

S23. TOCSY Spectrum of Microginin KR801 (**2**) in DMSO-d_6

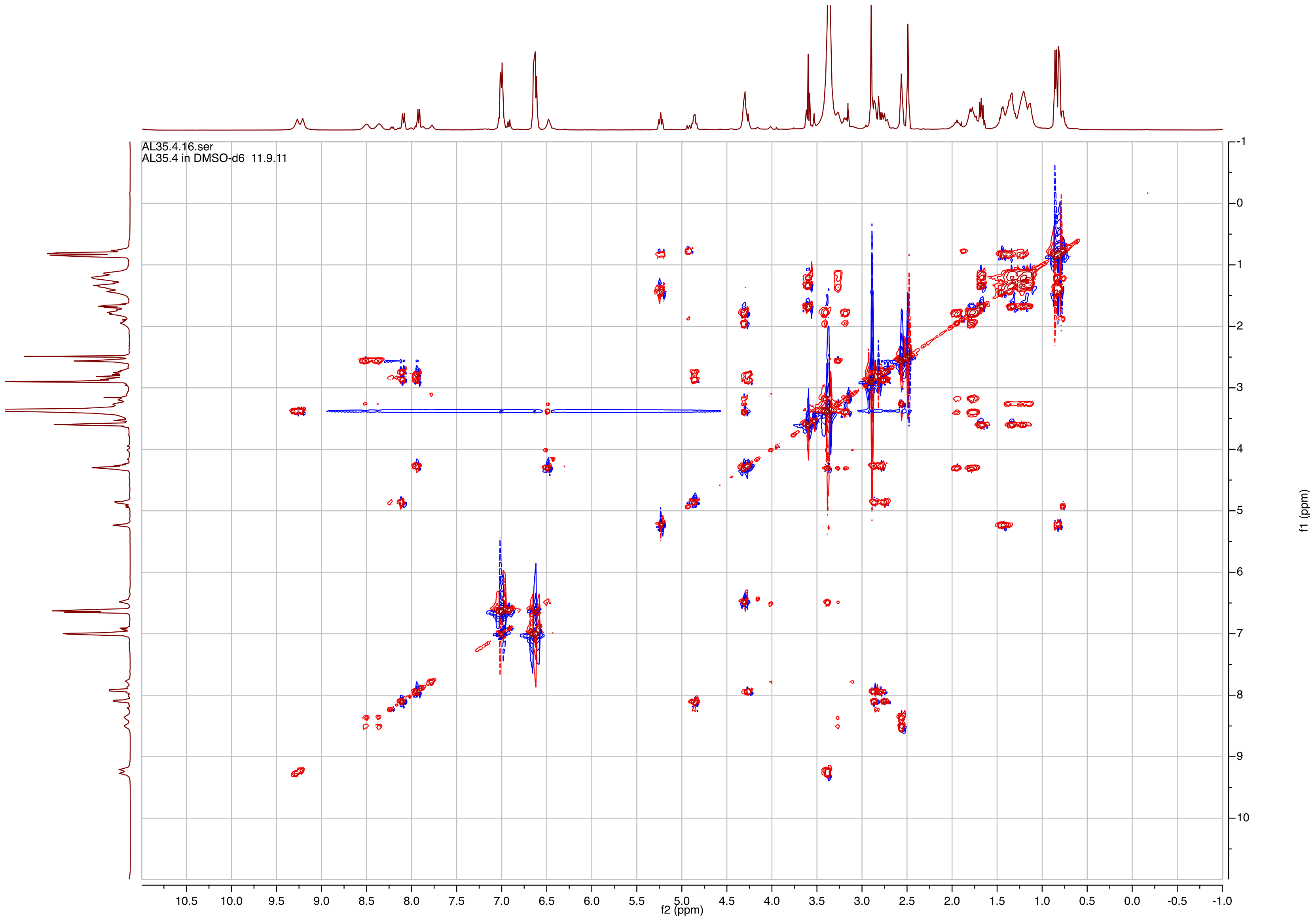

S24. ROESY Spectrum of Microginin KR801 (**2**) in DMSO-d_6

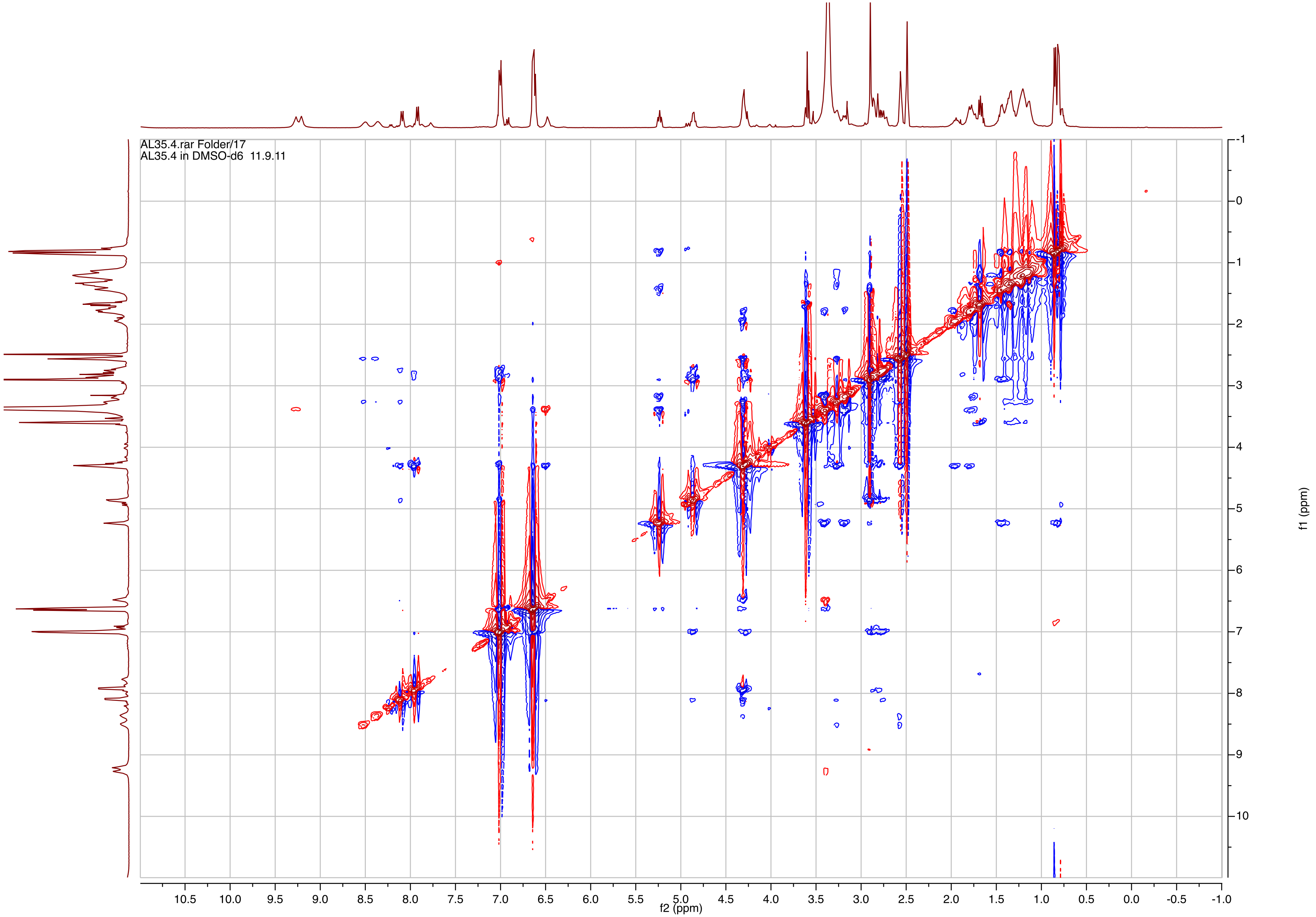

S25. HR ESI MS data of Microginin KR801 (**2**)

Elemental Composition Report

Single Mass Analysis
Tolerance = 2.0 PPM / DBE: min = -1.5, max = 50.0
Element prediction: Off
Number of isotope peaks used for i-FIT = 2

Monoisotopic Mass, Even Electron Ions
299 formula(e) evaluated with 2 results within limits (up to 50 closest results for each mass)
Elements Used:
C: 35-45 H: 55-65 N: 0-10 O: 5-15 Cl: 0-3
AL35.4 Anat Iodin
carmeli683 39 (1.736) Cm (38:39-5:21x10.000) 1: TOF MS ES+ 2.84e+004

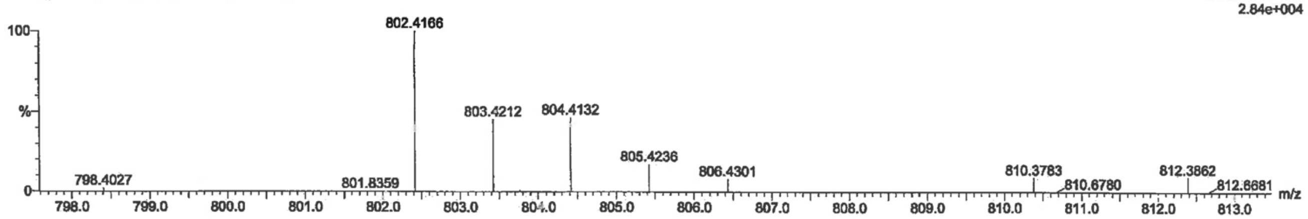

Minimum:				-1.5			
Maximum:		10.0	2.0	50.0			

Mass	Calc. Mass	mDa	PPM	DBE	i-FIT	i-FIT (Norm)	Formula
802.4166	802.4171	-0.5	-0.6	18.5	35.8	1.1	C42 H57 N9 O5 Cl
	802.4158	0.8	1.0	13.5	35.1	0.4	C41 H61 N5 O9 Cl

S26. **Table S4**. NMR Data (500/125 MHz) of Microginin KR835 (**3**) in DMSO-d_6

Position	δ_C	δ_H Multiplicity, J (Hz)	HMBC correlations	COSY correlations	NOESY correlations
Ahda 1	170.1 C		Ahda-2,2-OH, ^{1}Tyr-2,NH		
2	68.5 CH	4.30 brd, 5.6	Ahda-2-OH	Ahda-2-OH,3	Ahda-2-OH,3,3-NH$_2$, *N*CH$_3$,4,4',5',5', ^{1}Tyr-NH
2-OH		6.46 d, 6.0		Ahda-2	Ahda-2,3,3-*N*CH$_3$,4,6, ^{1}Tyr-NH
3	60.2 CH	3.27 brm	Ahda-2,2-OH,3-*N*CH$_3$	Ahda-2,3-NH$_2$,4,4'	Ahda-2,2-OH,3-NH$_2$, *N*CH$_3$,4,4',5',5'
3-NH$_2$		8.35 brs		Ahda-3,3-NH',3-*N*CH$_3$	Ahda-2,3,3-*N*CH$_3$
		8.48 brs		Ahda-3,3-NH',3-*N*CH$_3$	Ahda-2,3,3-*N*CH$_3$
3-*N*CH$_3$	30.8 CH$_3$	2.56 brt, 4.7		Ahda-3-NH$_2$	Ahda-2,2-OH,3,3-NH$_2$, 4,4'
4	26.0 CH$_2$	1.40 m	Ahda-2	Ahda-3,4',5,5'	Adha-2,3
		1.33 m		Ahda-3,4,5,5'	Adha-2,3
5	25.0 CH$_2$	1.24 m	Ahda-4,4',6,6'	Ahda-4,4',5',6,6'	Adha-2,3
		1.14 m		Ahda-4,4',5,6,6'	Adha-2,3
6	28.9 CH$_2$	1.22 m	Ahda-5,7,8		
		1.15 m			
7	27.8 CH$_2$	1.24 m	Ahda-8,9		
8	25.4 CH$_2$	1.43 m	Ahda-9,10	Ahda-7,9	
9	43.0 CH$_2$	2.12 td, 7.3,5.9	Ahda-7,8,10	Ahda-8,10	
10	75.0 CH	6.29 t, 5.9	Ahda-8,9	Ahda-9	
^{1}Tyr 1	171.0 C		^{1}Tyr-2,3,3', *N*MeLeu-2, *N*CH$_3$		
2	50.6 CH	4.86 ddd, 8.0,7.7,6.4	^{1}Tyr-3,3',NH	^{1}Tyr-2-NH,3,3'	^{1}Tyr-2-NH,3,3',5,5'
2-NH		8.09 d, 8.0		^{1}Tyr-2	^{1}Tyr-2,3,3', Ahda-2,2-OH,3
3	36.4 CH$_2$	2.86 m	^{1}Tyr-2,5,5',NH	^{1}Tyr-2,3'	^{1}Tyr-2,3',5,5',NH
		2.74 m		^{1}Tyr-2,3	^{1}Tyr-2,3,5,5',NH
4	126.9 C		^{1}Tyr-2,3,3',6,6'		
5,5'	130.3 CH	7.00 d, 8.3	^{1}Tyr-3,3',5',5,6,6'	^{1}Tyr-6,6'	^{1}Tyr-2,3,3',6,6'
6,6'	115.1 CH	6.62 d, 8.3	^{1}Tyr-5,5',6',6,7-OH	^{1}Tyr-5,5'	^{1}Tyr-5,5',7-OH

7	156.2 C		[1]Tyr-5,5’,6,6’,7-OH		
7-OH		9.26 s			[1]Tyr-6,6’
*N*MeLeu 1	168.5 C		*N*MeLeu-2,3,3’		
2	51.9 CH	5.23 dd, 8.2,6.6	*N*MeLeu-3,3’,4,*N*Me	*N*MeLeu-3,3’	*N*MeLeu-3,3’,5,6,*N*Me, Pro-5
2-*N*CH_3	30.2 CH_3	2.90 s	*N*MeLeu-2		*N*MeLeu-2,3,3’, [2]Tyr-2,3
3	37.2 CH_2	1.46 m	*N*MeLeu-2,4,5,6	*N*MeLeu-2,3’,4	*N*MeLeu-2,5,6,*N*Me
		1.42 m		*N*MeLeu-2,3,4	*N*MeLeu-2,5,6,*N*Me
4	24.2 CH	1.37 m	*N*MeLeu-2,3,3’,5,6	*N*MeLeu-3,3’,5,6	*N*MeLeu-5,6
5	22.4 CH_3	0.81 d, 6.2	*N*MeLeu-3,3’,4,6	*N*MeLeu-4	*N*MeLeu-2,3,3’,4,6
6	23.1 CH_3	0.85 d, 6.3	*N*MeLeu-3,3’,4,5	*N*MeLeu-4	*N*MeLeu-2,3,3’,4,5
Pro 1	171.5 C		Pro-2,3,3’, [2]Tyr-NH		
2	59.4 CH	4.32 dd, 8.5,5.0	Pro-4’	Pro-3,3’	Pro-3’, [2]Tyr-NH
3	29.1 CH_2	1.94 m	Pro-2,4,5’	Pro-2,3’,4,4’,5’	Pro-2,3’,4
		1.79 m		Pro-2,3,4,4’	Pro-2,3,4’
4	24.3 CH_2	1.80 m	Pro-2,3,3’,5,5’	Pro-3,3’,5,5’	Pro-3,5’
		1.73 m		Pro-3,3’,5,5’	Pro-3’
5	46.7 CH_2	3.40 m	Pro-2	Pro-4,4’,5’	Pro-4, *N*MeLeu-2, [2]Tyr-7-OH
		3.19 dt, 9.3,7.3		Pro-3,4,4’,5	Pro-2,4,5, *N*MeLeu-2
[2]Tyr 1	173.1 C		[2]Tyr-2,3,3’,NH		
2	54.0 CH	4.26 td, 7.7,5.7	[2]Tyr-3,3’,NH	[2]Tyr-3,3’,NH	[2]Tyr-3,5,5’,NH
2-NH		7.92 d, 7.7		[2]Tyr-2	[2]Tyr-2,3’, Pro-2
3	36.1 CH_2	2.87 m	[2]Tyr-2,5,5’,NH	[2]Tyr-2,3’	[2]Tyr-2,3’,5,5’
		2.78 m		[2]Tyr-2,3	[2]Tyr-2,3,5,5’,NH
4	127.6 C		[2]Tyr-2,3,3’,6,6’		
5,5’	130.3 CH	7.01d, 8.0	[2]Tyr-3,3’,5’,5,6,6’	[2]Tyr-6,6’	[2]Tyr-2,3,6,6’
6,6’	115.1 CH	6.64 d, 8.0	[2]Tyr-5,5’,6’,6,7-OH	[2]Tyr-5,5’	[2]Tyr-5,5’,7-OH
7	156.1 C		[2]Tyr-5,5’,6,6’,7-OH		
7-OH		9.20 s			[2]Tyr-6,6’

S28. ^{1}H NMR Spectrum of Microginin KR835 (**3**) in DMSO-*d*$_6$

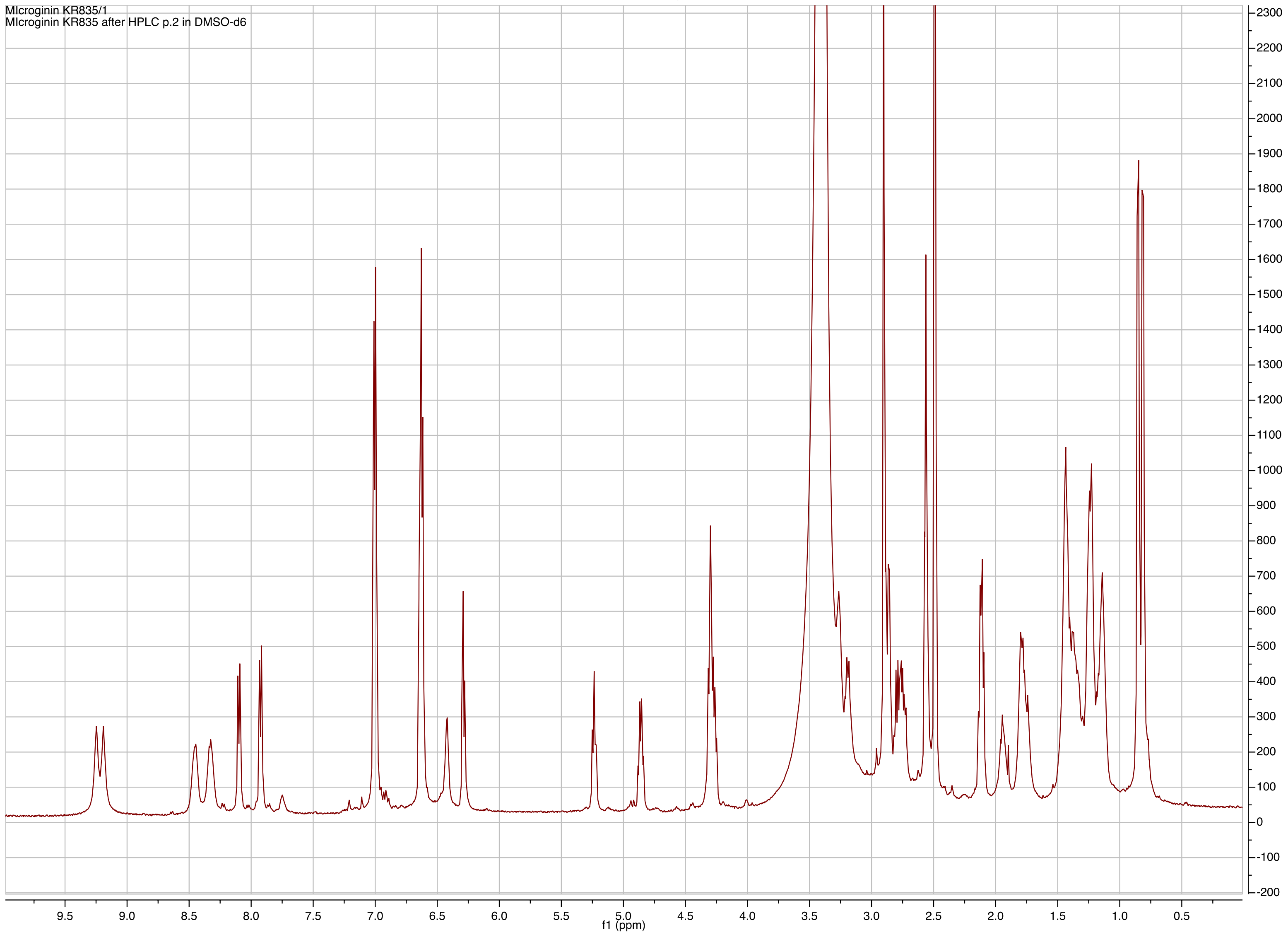

S29. ^{13}C NMR Spectrum of Microginin KR835 (**3**) in DMSO-d_6

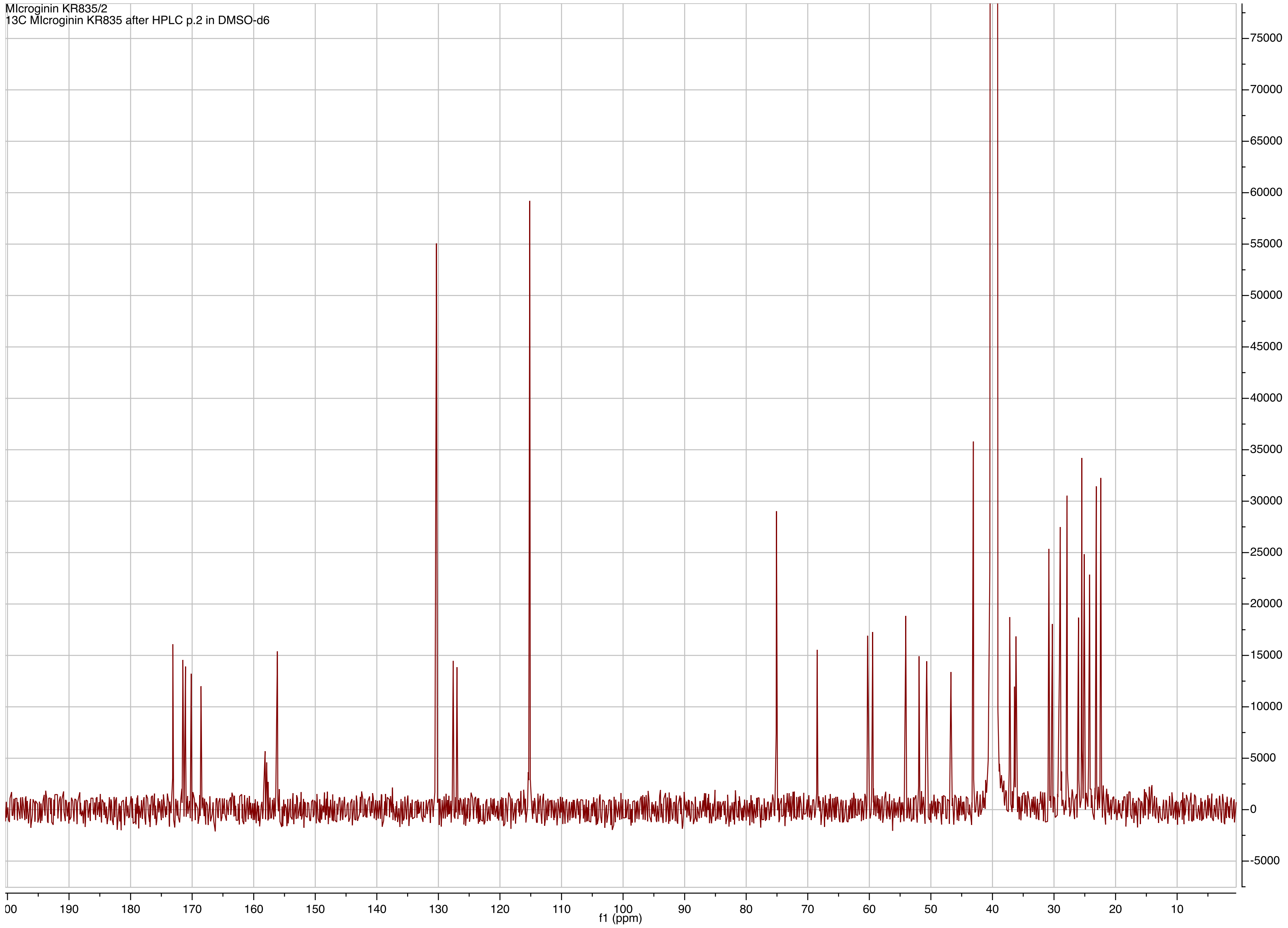

S30. HSQC Spectrum Microginin KR835 (**3**) in DMSO-d_6

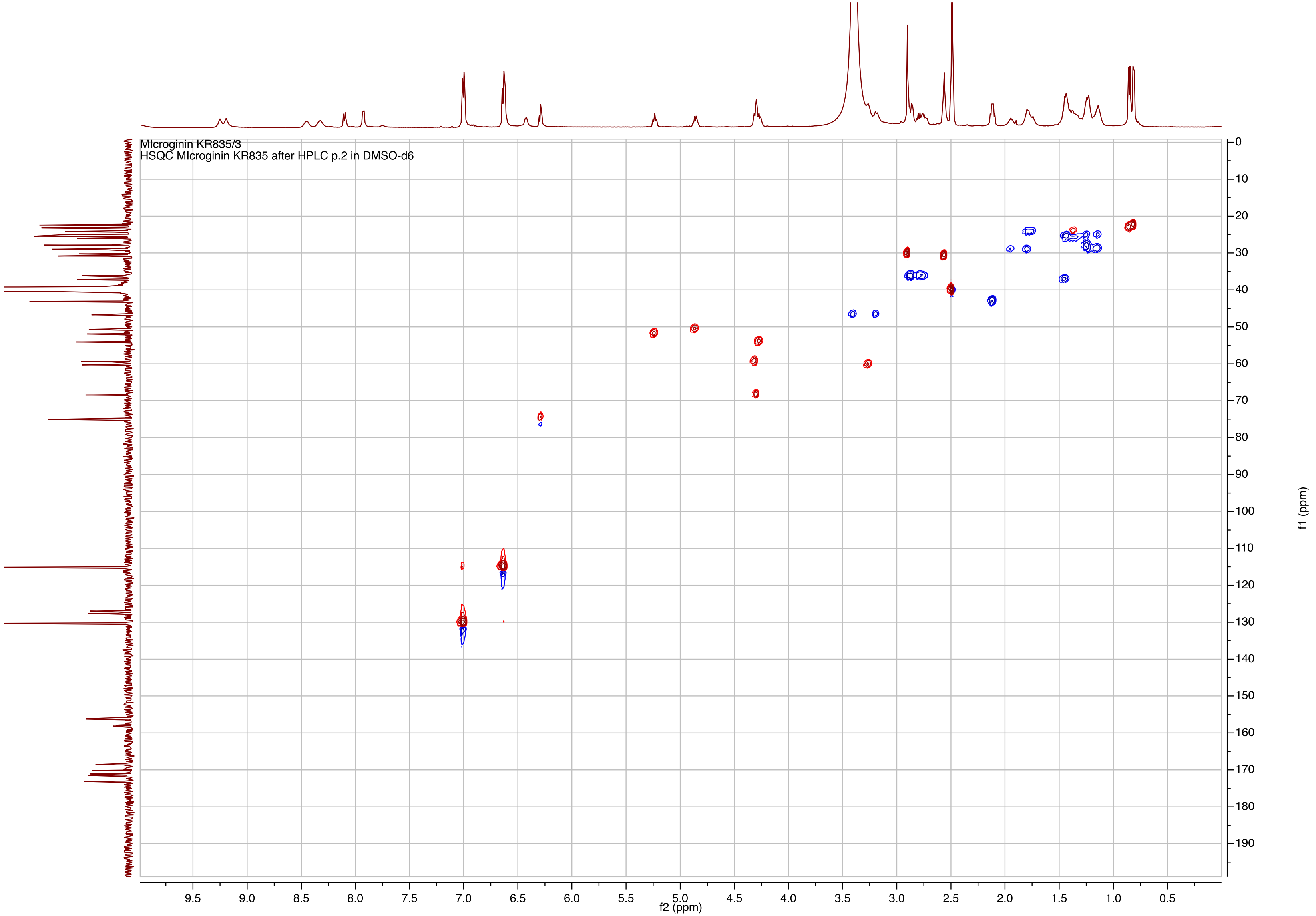

S31. HMBC Spectrum of Microginin KR835 (**3**) in DMSO-d_6

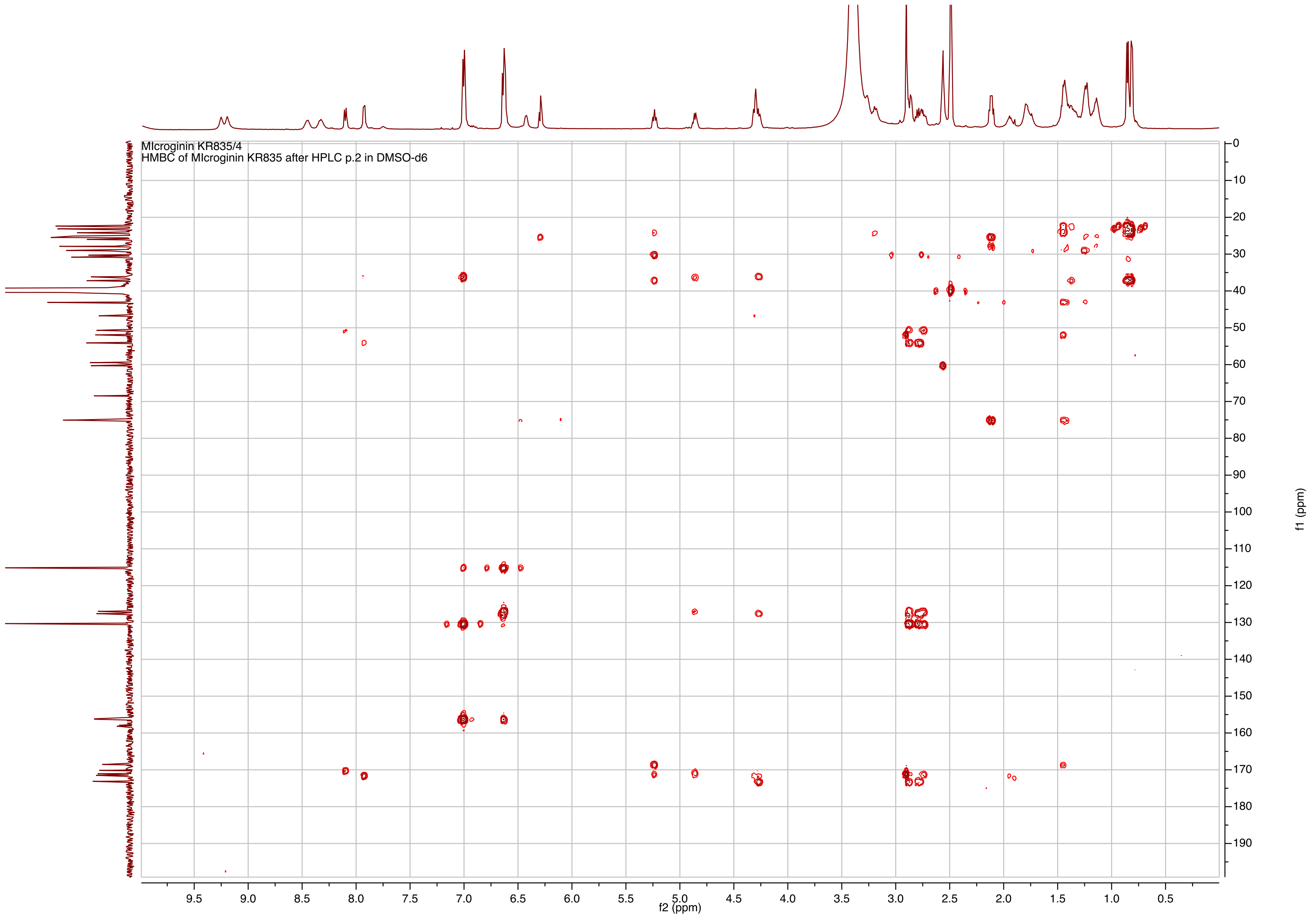

S32. COSY Spectrum of Microginin KR835 (**3**) in DMSO-d_6

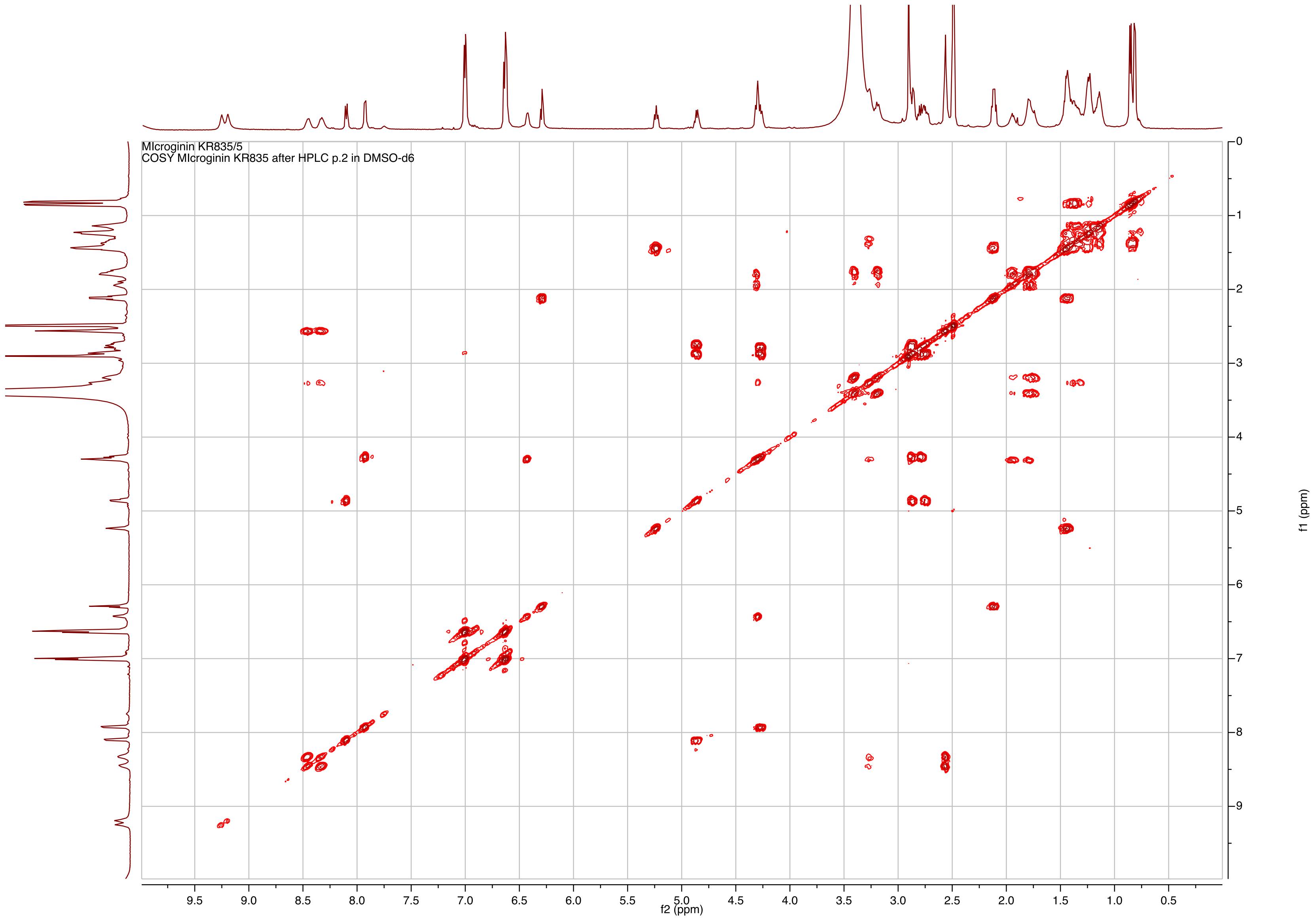

S33. TOCSY Spectrum of Microginin KR835 (**3**) in DMSO-d_6

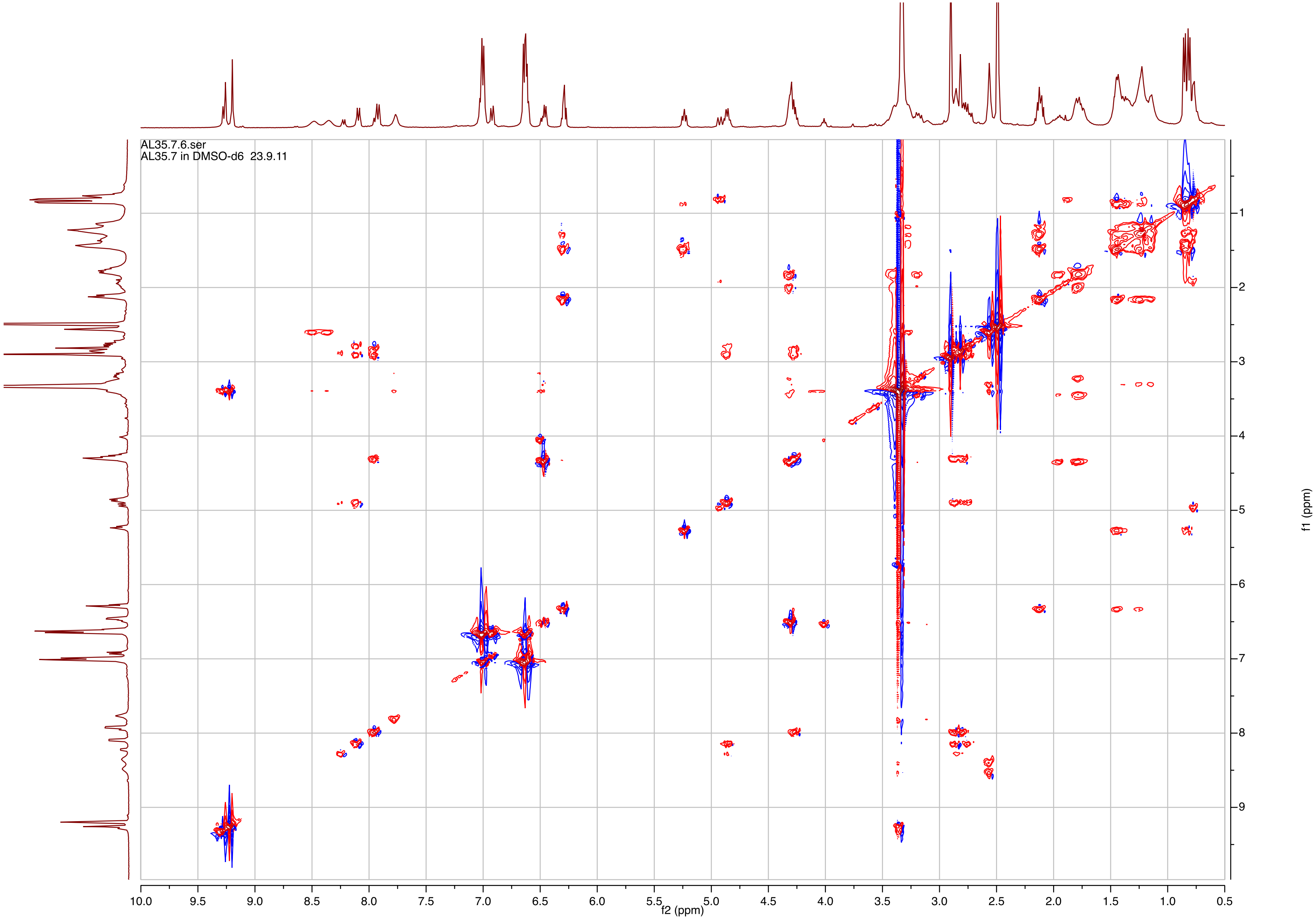

S34. ROESY Spectrum of Microginin KR835 (**3**) in DMSO-d_6

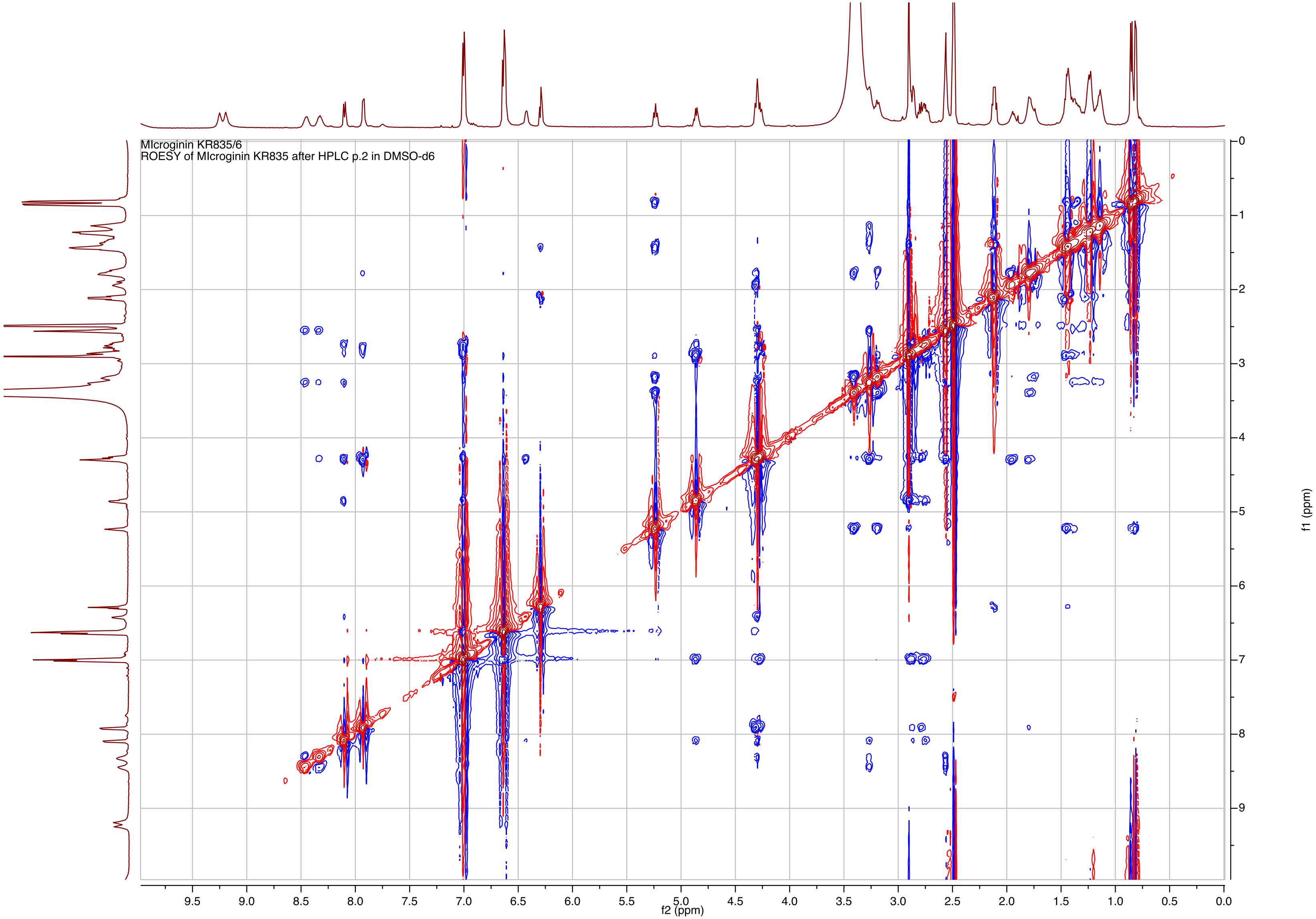

S35. HR ESI MS data of Microginin KR835 (**3**)

Elemental Composition Report Page 1

Single Mass Analysis
Tolerance = 3.0 PPM / DBE: min = -1.5, max = 50.0
Element prediction: Off
Number of isotope peaks used for i-FIT = 2

Monoisotopic Mass, Even Electron Ions
446 formula(e) evaluated with 7 results within limits (up to 50 closest results for each mass)
Elements Used:
C: 35-45 H: 55-65 N: 0-10 O: 5-15 Cl: 0-5
AL35.7 Anat Iodin
carmeli682 39 (1.736) Cm (39:52-(70:72+69:72)x10.000) 1: TOF MS ES+ 3.22e+004

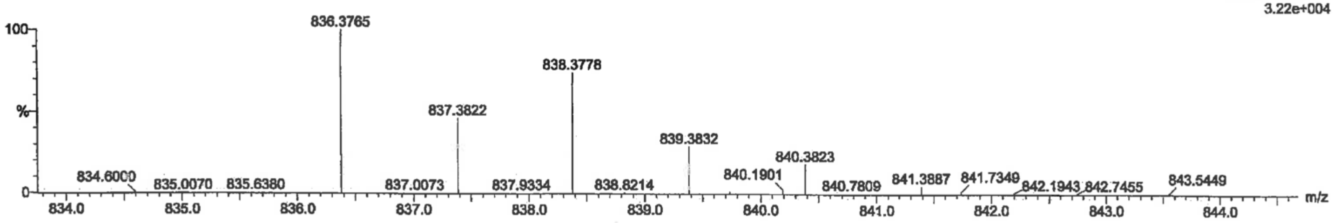

Minimum:				-1.5			
Maximum:		10.0	3.0	50.0			
Mass	Calc. Mass	mDa	PPM	DBE	i-FIT	i-FIT (Norm)	Formula
836.3765	836.3768	-0.3	-0.4	13.5	76.8	3.8	C41 H60 N5 O9 Cl2
	836.3760	0.5	0.6	9.5	76.6	3.6	C36 H61 N9 O7 Cl3
	836.3755	1.0	1.2	8.5	73.8	0.8	C40 H64 N O13 Cl2
	836.3750	1.5	1.8	18.5	79.6	6.6	C42 H55 N7 O9 Cl
	836.3781	-1.6	-1.9	18.5	78.8	5.8	C42 H56 N9 O5 Cl2
	836.3746	1.9	2.3	4.5	79.9	6.9	C35 H65 N5 O11 Cl3
	836.3786	-2.1	-2.5	8.5	73.8	0.7	C40 H65 N3 O9 Cl3

S36. **Table S5**. NMR Data (500/125 MHz) of Microginin KR787 (**4**) in DMSO-d_6

Position	δ_C	δ_H Multiplicity, *J* (Hz)	HMBC correlations	COSY correlations	NOESY correlations
Ahda 1	170.2 C		^{1}Tyr-NH		
2	70.9 CH	4.14 dd, 5.4,2.5		Ahda-2-OH,3	Ahda-2-OH,3,3-NH_2,4,4',5',5', ^{1}Tyr-NH
2-OH		6.35 d, 5.4		Ahda-2	Ahda-2, ^{1}Tyr-NH
3	53.4 CH	3.27 brm	Ahda-2,NH$_{\underline{2}}$	Ahda-2,3-NH_2,4,4'	Ahda-2,3-NH$_{\underline{2}}$,4',5,5', ^{1}Tyr-NH
3-NH$_{\underline{2}}$		7.83 brd, 2.6		Ahda-3	Ahda-2,3
4	26.8 CH_2	1.37 m		Ahda-3,4',5,5'	Adha-3
		1.22 m		Ahda-3,4,5,5'	Adha-2,3
5	25.0 CH_2	1.26 m		Ahda-4,4',5',6	Adha-2,3
		1.12 m		Ahda-4,4',5,6	Adha-2,3
6	29.0 CH_2	1.13 m	Ahda-5,8	Ahda-7	
7	28.2 CH_2	1.22 m	Ahda-9	Ahda-6,8	
8	26.4 CH_2	1.34 m	Ahda-9,10	Ahda-7,9	
9	32.2 CH_2	1.68 tt, 7.6,6.7	Ahda-8,10	Ahda-8,10	Ahda-10
10	45.6 CH_2	3.60 t, 6.7	Ahda-8,9	Ahda-9	Ahda-7,8,9
^{1}Tyr 1	171.2 C		^{1}Tyr-2,3,3', *N*MeLeu-2, *N*CH_3		
2	50.7 CH	4.84 ddd, 8.2,7.4,6.2	^{1}Tyr-3,3'	^{1}Tyr-2-NH,3,3'	^{1}Tyr-2-NH,3,3',5,5', *N*MeLeu-*N*Me
2-NH		8.04 d, 8.2		^{1}Tyr-2	^{1}Tyr-2,3,3',5,5', Ahda-2,2-OH,3
3	36.3 CH_2	2.88 m	^{1}Tyr-2,5,5'	^{1}Tyr-2,3'	^{1}Tyr-2,3',5,5',NH
		2.74 dd, 13.6,7.9		^{1}Tyr-2,3	^{1}Tyr-2,3,5,5',NH
4	127.0 C		^{1}Tyr-2,3,3',6,6'		
5,5'	130.4 CH	7.00 d, 8.1	^{1}Tyr-3,3',5',5,6,6'	^{1}Tyr-6,6'	^{1}Tyr-2,3,3',6,6',NH
6,6'	115.2 CH	6.62 d, 8.1	^{1}Tyr-5,5',6',6,7-OH	^{1}Tyr-5,5'	^{1}Tyr-5,5',7-OH
7	156.2 C		^{1}Tyr-5,5',6,6',7-OH		
7-OH		9.24 s			^{1}Tyr-6,6'
*N*MeLeu 1	168.5 C		*N*MeLeu-2,3,3'		
2	52.0 CH	5.23 dd, 8.1,6.9	*N*MeLeu-3,3',4,*N*Me	*N*MeLeu-3,3'	*N*MeLeu-3,3',4,5,6,*N*Me, Pro-5,5'

2-*N*CH_3	30.2 CH_3	2.89 s	*N*MeLeu-2		*N*MeLeu-2,3,3’,4, Pro-5’
3	37.2 CH_2	1.45 m	*N*MeLeu-2,4,5,6	*N*MeLeu-2,3’,4	*N*MeLeu-2,5,6,*N*Me
		1.42 m		*N*MeLeu-2,3,4	*N*MeLeu-2,5,6,*N*Me
4	24.3 CH	1.37 m	*N*MeLeu-2,3,3’,5,6	*N*MeLeu-3,3’,5,6	*N*MeLeu-*N*Me
5	22.3 CH_3	0.80 d, 6.1	*N*MeLeu-3,3’,4,6	*N*MeLeu-4	*N*MeLeu-2,3,3’
6	23.2 CH_3	0.85 d, 6.2	*N*MeLeu-3,3’,4,5	*N*MeLeu-4	*N*MeLeu-2,3,3’
Pro 1	171.5 C		Pro-2, ^{2}Tyr-NH		
2	59.4 CH	4.30 m		Pro-3,3’	Pro-3,3’, ^{2}Tyr-NH
3	29.1 CH_2	1.94 m	Pro-5’	Pro-2,3’,4,4’,5,5’	Pro-2,3’
		1.81 m		Pro-2,3,4,4’	Pro-2,3, ^{2}Tyr-NH
4	24.1 CH_2	1.81 m	Pro-2	Pro-3,3’,5,5’	Pro-2
		1.76 m		Pro-3,3’,5,5’	
5	46.7 CH_2	3.40 m		Pro-3,4,4’,5’	Pro-3,5, *N*MeLeu-2,
		3.18 dt, 9.0,7.2		Pro-3,4,4’,5	Pro-3,4,4’, *N*MeLeu-2,*N*Me
^{2}Tyr 1	173.1 C		^{2}Tyr-2,3,3’		
2	54.1 CH	4.27 td, 7.6,5.9	^{2}Tyr-3,3’,NH	^{2}Tyr-3,3’,NH	^{2}Tyr-3,3’,5,5’,NH
2-NH		7.93 d, 7.6		^{2}Tyr-2	^{2}Tyr-2,3,3’,5,5 Pro-2,3’
3	36.2 CH_2	2.88 m	^{2}Tyr-2,5,5’	^{2}Tyr-2,3’	^{2}Tyr-2,3’,5,5’,NH
		2.78 dd, 16.7,8.5		^{2}Tyr-2,3	^{2}Tyr-2,3,5,5’,NH
4	127.6 C		^{2}Tyr-2,3,3’,6,6’		
5,5’	130.3 CH	7.00 d, 8.3	^{2}Tyr-3,3’,5’,5,6,6’	^{2}Tyr-6,6’	^{2}Tyr-2,3,3’,6,6’,NH
6,6’	115.2 CH	6.64 d, 8.3	^{2}Tyr-5,5’,6’,6,7-OH	^{2}Tyr-5,5’	^{2}Tyr-5,5’,7-OH
7	156.1 C		^{2}Tyr-5,5’,6,6’		
7-OH		9.19 s			^{2}Tyr-6,6’

S38. ^{1}H NMR Spectrum of Microginin KR787 (**4**) in DMSO-d_6

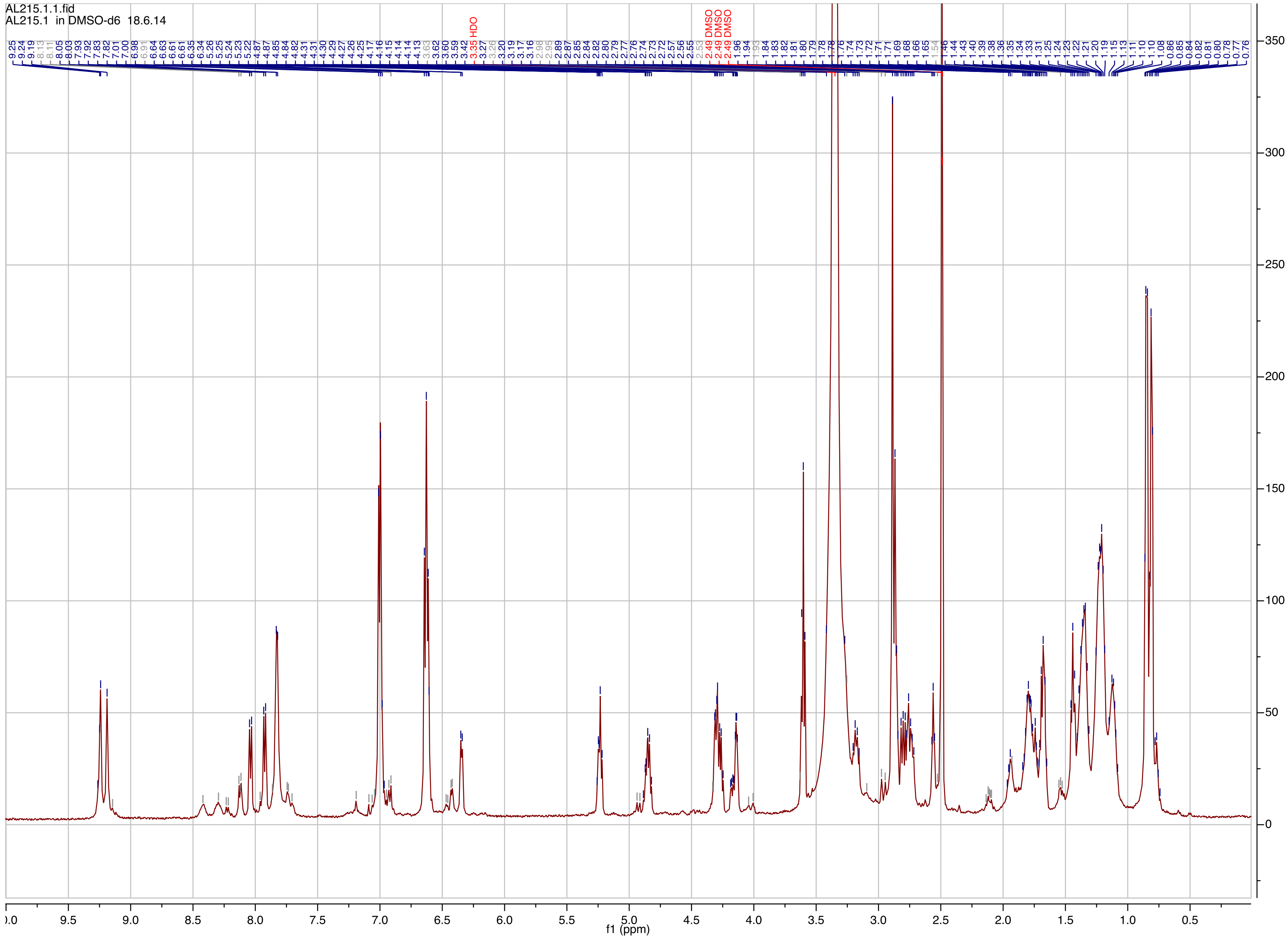

S39. ^{13}C NMR Spectrum of Microginin KR787 (**4**) in DMSO-d_6

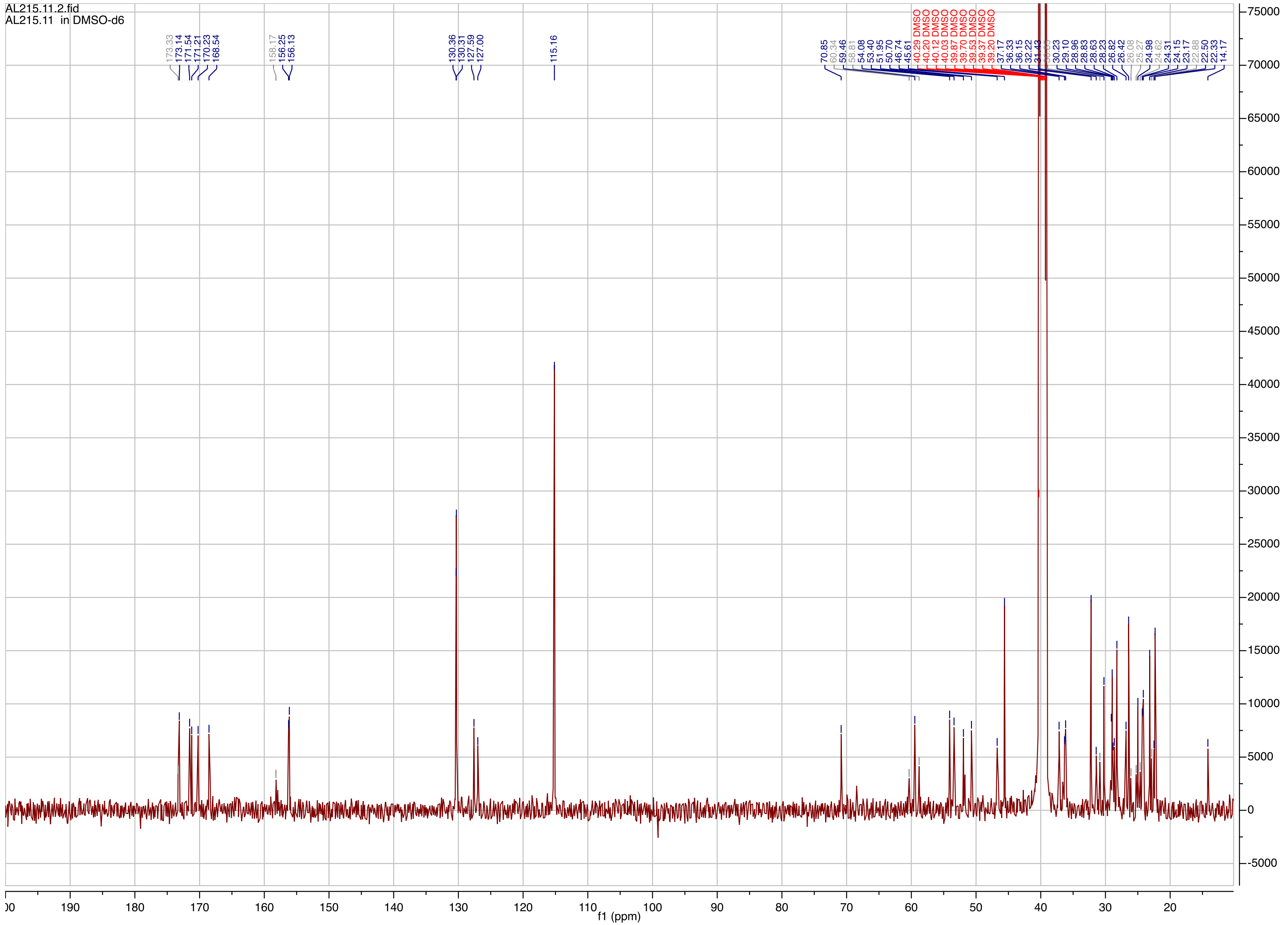

S40. HSQC Spectrum Microginin KR787 (**4**) in DMSO-d_6

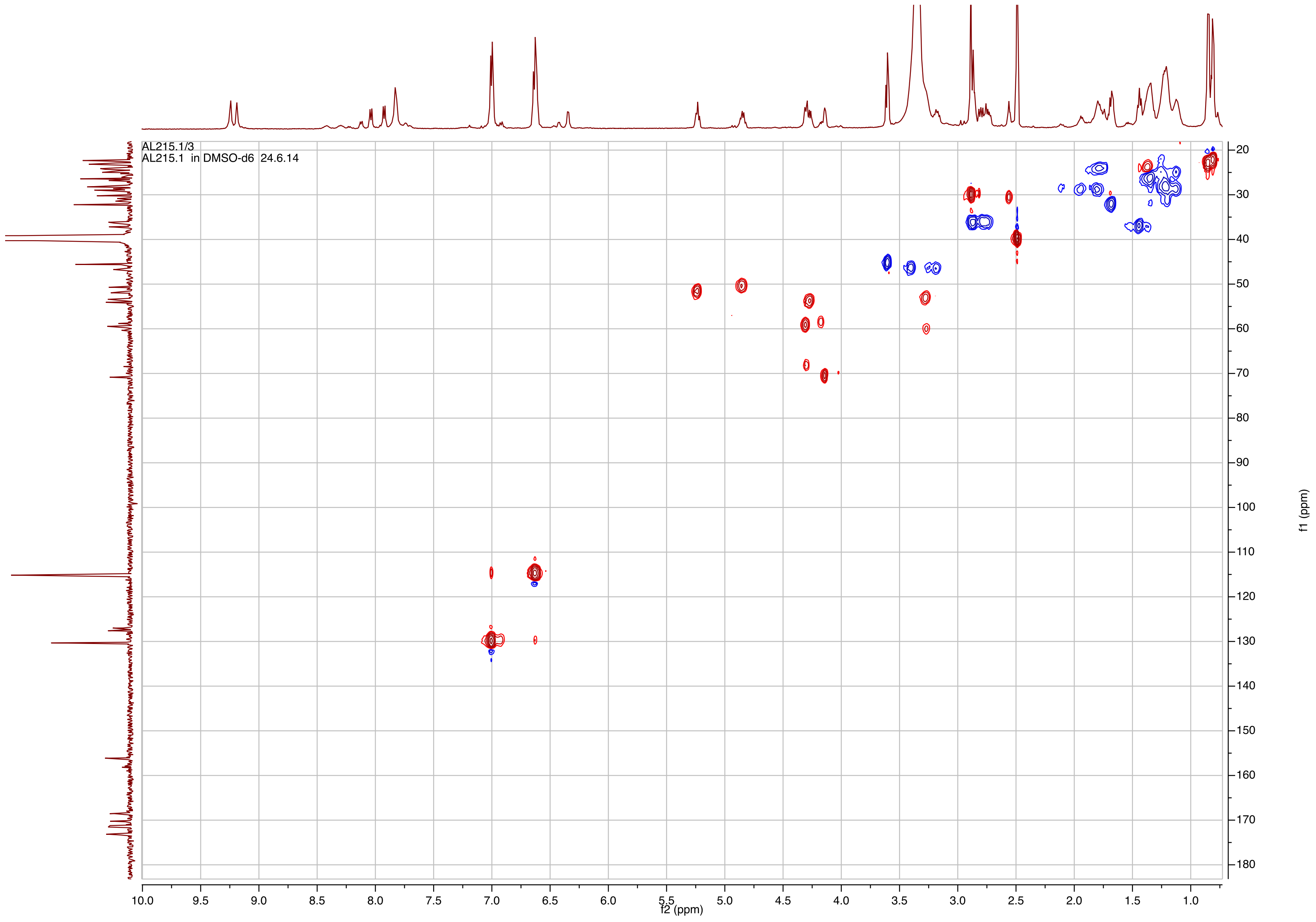

S41. HMBC Spectrum of Microginin KR787 (**4**) in DMSO-d_6

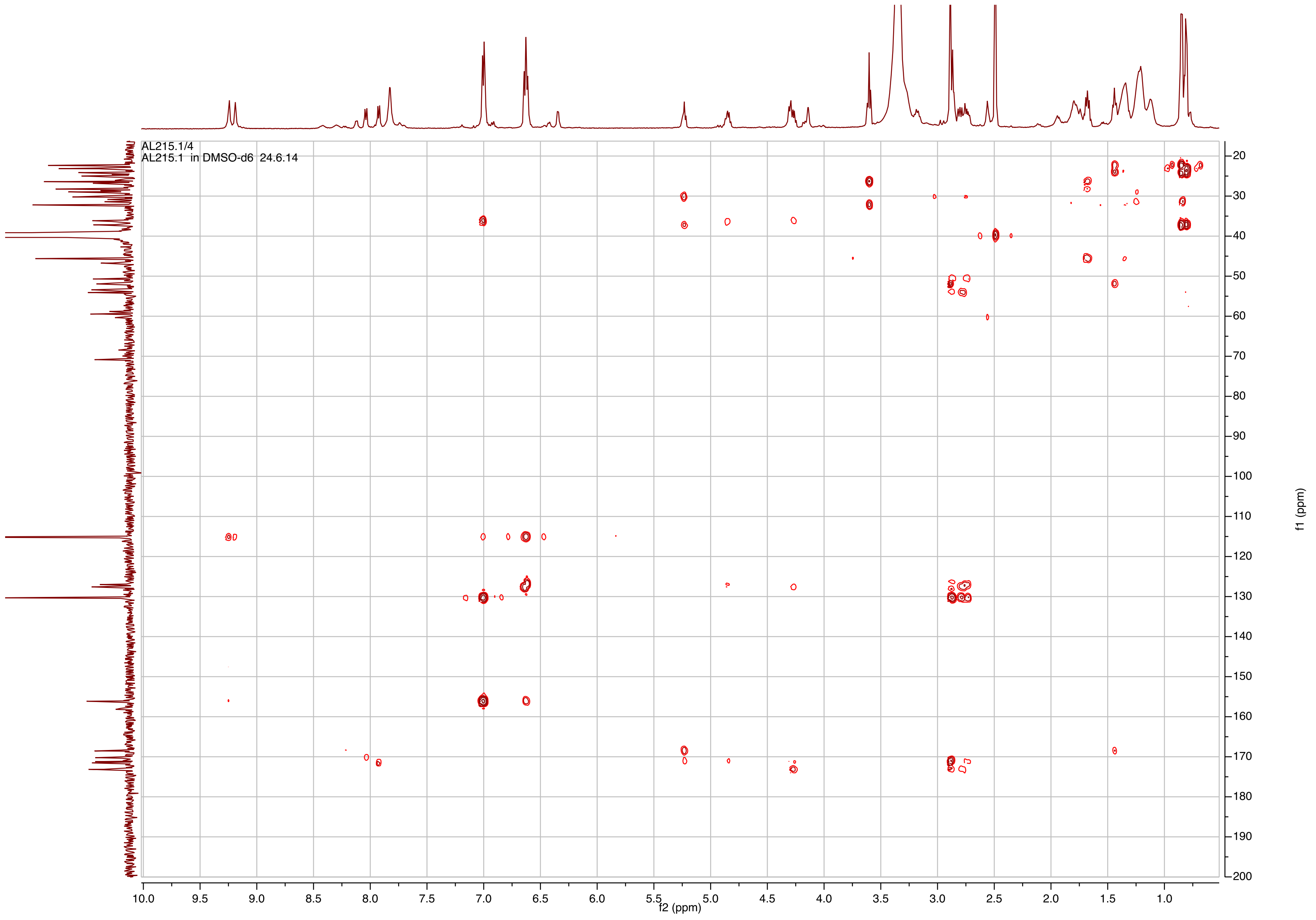

S42. COSY Spectrum of Microginin KR787 (**4**) in DMSO-d_6

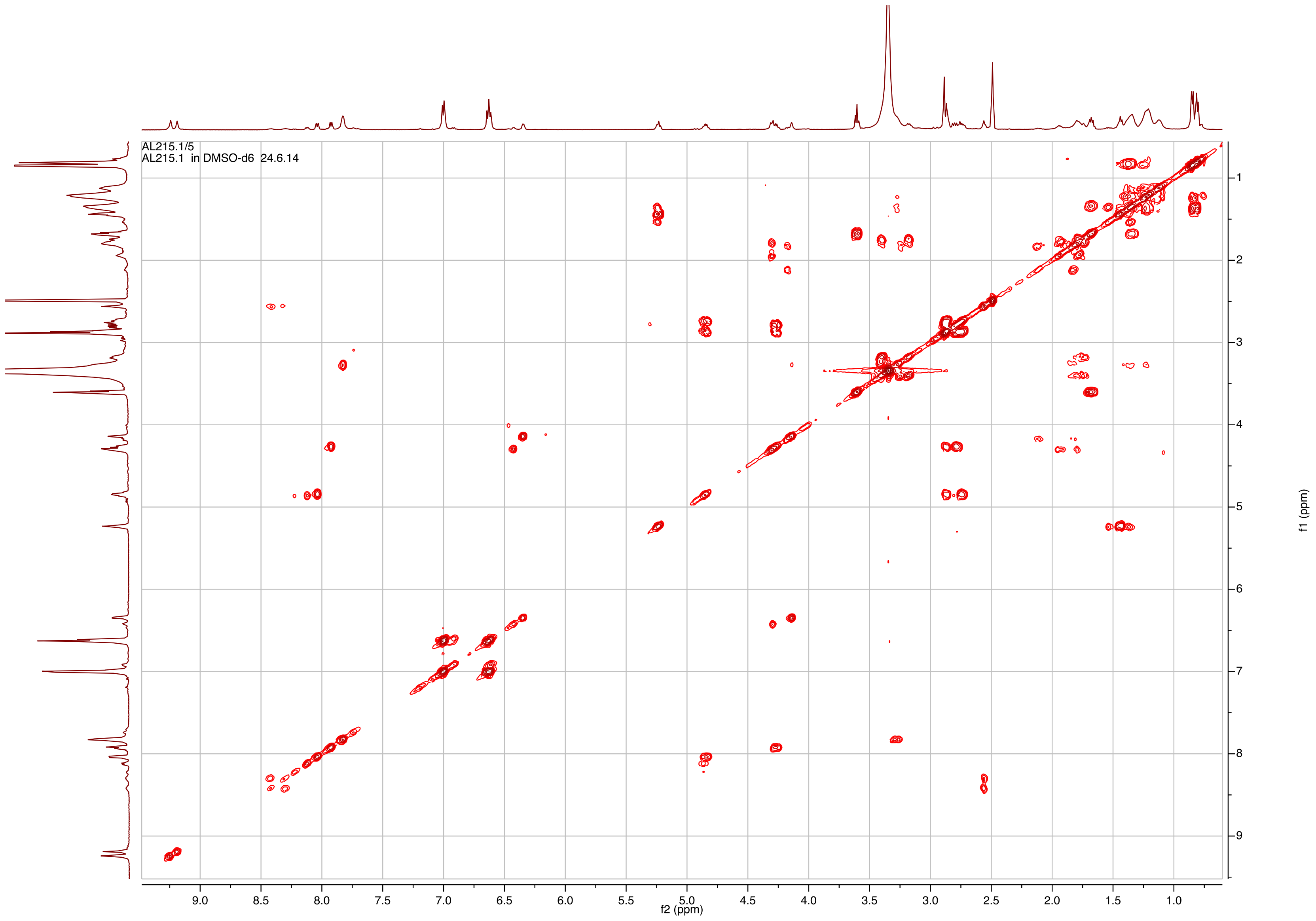

S43. TOCSY Spectrum of Microginin KR787 (**4**) in DMSO-d_6

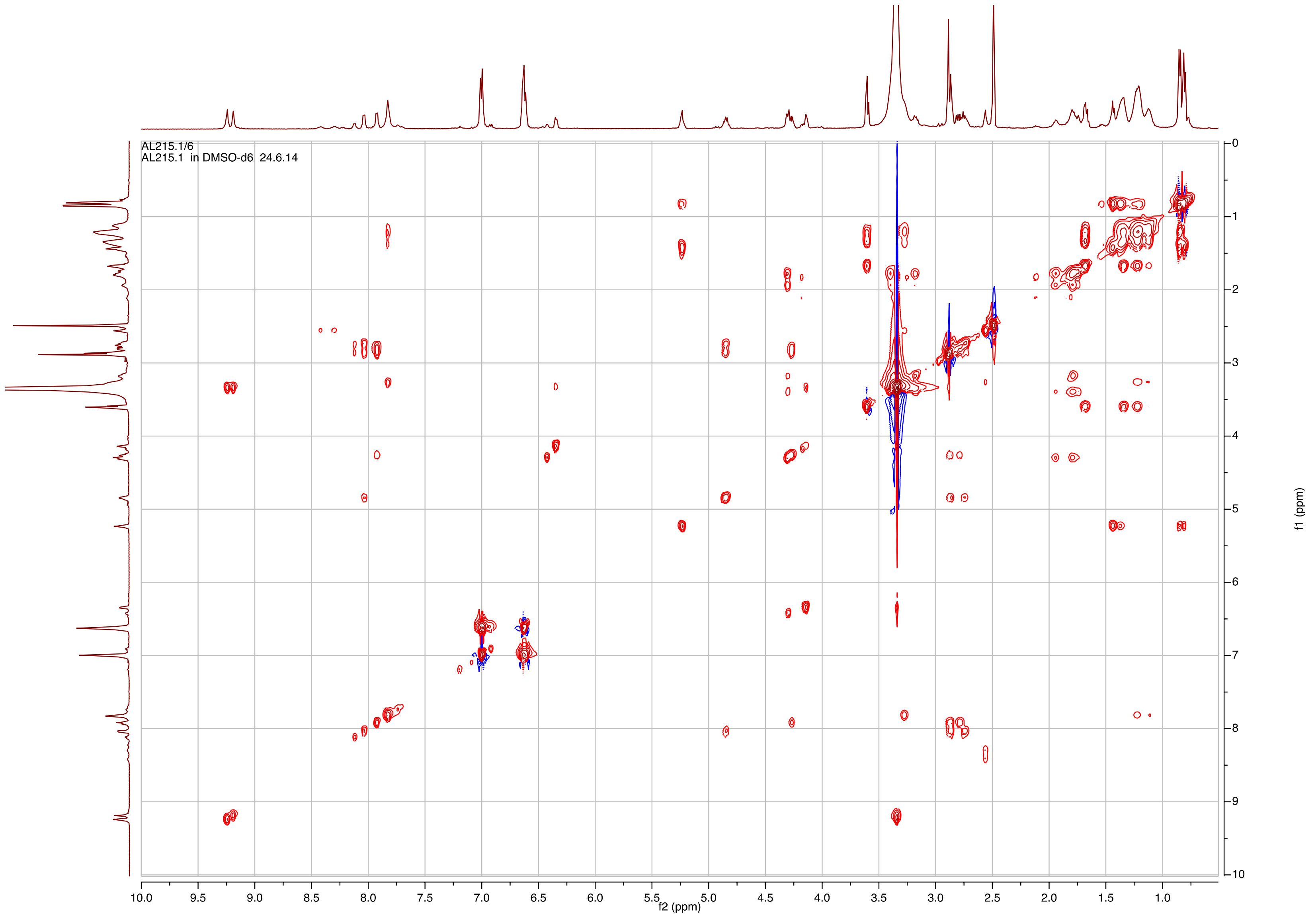

S44. ROESY Spectrum of Microginin KR787 (**4**) in DMSO-d_6

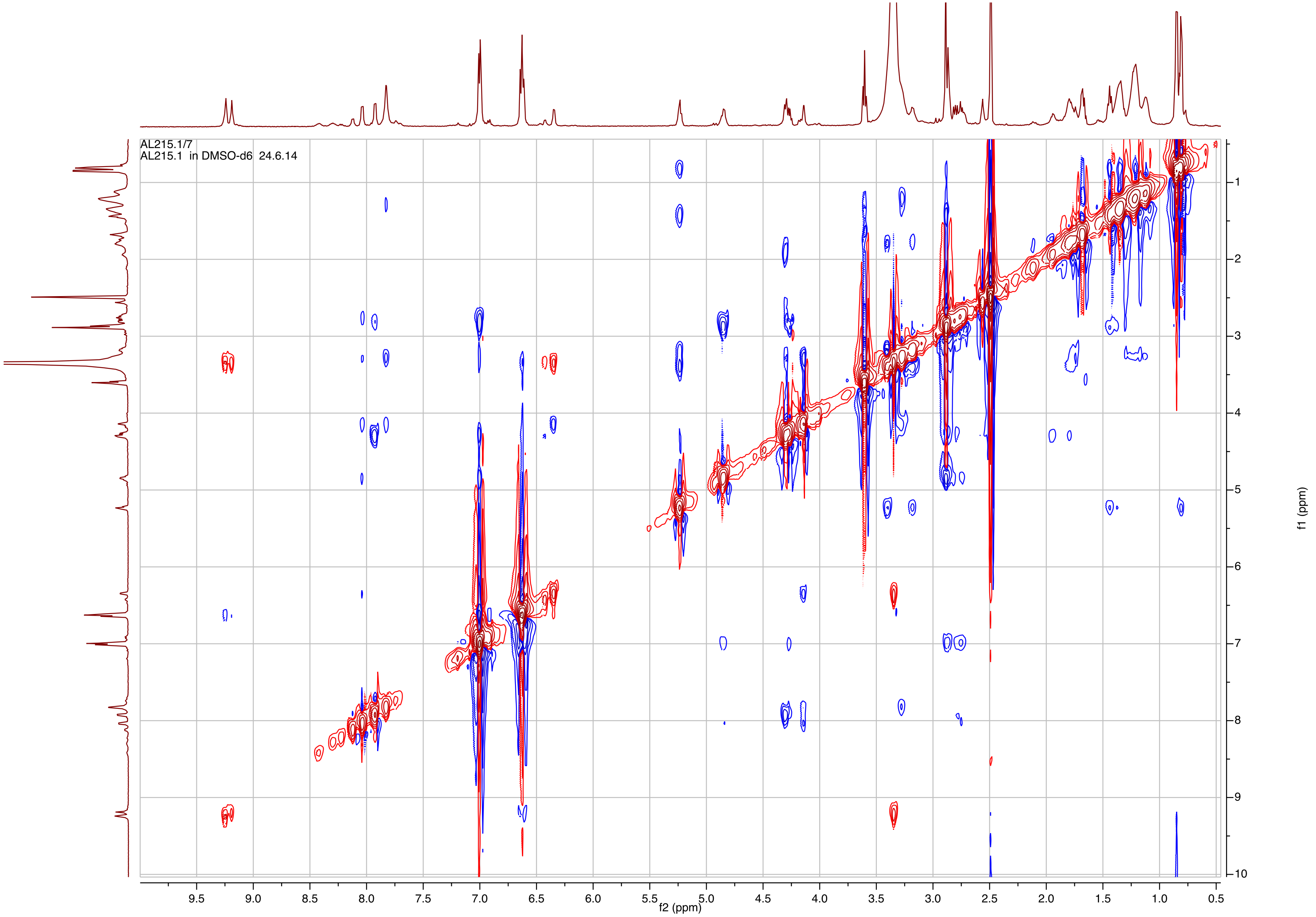

S45. HR ESI MS data of Microginin KR787 (**4**)

Elemental Composition Report

Single Mass Analysis
Tolerance = 2.0 PPM / DBE: min = -1.5, max = 50.0
Element prediction: Off
Number of isotope peaks used for i-FIT = 3

Monoisotopic Mass, Even Electron Ions
98 formula(e) evaluated with 2 results within limits (all results (up to 1000) for each mass)
Elements Used:
C: 38-45 H: 55-65 N: 0-10 O: 0-15 Cl: 1-1

AL129.1 Anat Iodin
carmel688b 27 (1.210) Cm (27:30) 1: TOF MS ES+ 1.73e+005

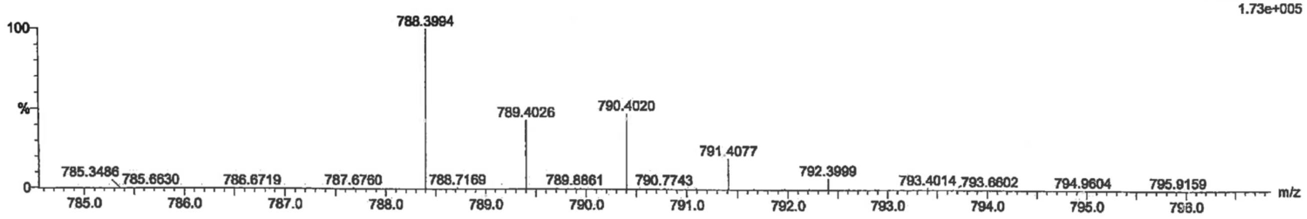

Minimum:				-1.5			
Maximum:		5.0	2.0	50.0			

Mass	Calc. Mass	mDa	PPM	DBE	i-FIT	i-FIT (Norm)	Formula
788.3994	788.3988	0.6	0.8	8.5	237.4	0.4	C39 H63 N O13 Cl
	788.4001	-0.7	-0.9	13.5	238.2	1.1	C40 H59 N5 O9 Cl ←

S46. **Table S6**. NMR Data (500/125 MHz) of Microginin KR604 (**5**) in DMSO-d_6

Position	δ_C	δ_H Multiplicity, *J* (Hz)	HMBC correlations	COSY correlations	NOESY correlations
Ahda 1	170.5 C		Ahda-2, ^{1}Tyr-NH		
2	68.7 CH	4.26 brd, 2.0		Ahda-3	Ahda-3,3-*N*CH_3, ^{1}Tyr-NH
2-OH		6.35 brs			
3	60.5 CH	3.14 brs	Ahda-2,3-*N*CH_3	Ahda-2,4,4’	Ahda-2,3-*N*CH_3
3-NH		8.00 brs			
3-*N*CH_3	31.4 CH_3	2.51 brs			Ahda-2,3
4	25.4 CH_2	1.34 m		Ahda-3,4’,5,5’	
		1.28 m		Ahda-3,4,5,5’	
5	24.2 CH_2	1.27 m	Ahda-4	Ahda-4,4’,5’,6	
		1.13 m		Ahda-4,4’,5,6	
6	28.6 CH_2	1.14 m	Ahda-5	Ahda-6	
7	29.2 CH_2	1.19 m	Ahda-8		
8	31.3 CH_2	1.20 m	Ahda-9,10		
9	22.2 CH_2	1.26 m	Ahda-8,10	Ahda-8,10	
10	14.1 CH_3	0.84 t, 6.6	Ahda-9	Ahda-9	
^{1}Tyr 1	171.0 C		^{1}Tyr-2,3,3’, *N*MeLeu-2, *N*CH_3		
2	50.4 CH	4.87 dt, 8.1,7.4	^{1}Tyr-3,3’	^{1}Tyr-2-NH,3,3’	^{1}Tyr-2-NH,3,3’, *N*MeLeu-*N*Me
2-NH		8.03 d, 8.1		^{1}Tyr-2	^{1}Tyr-2,3,3’, Ahda-2
3	36.6 CH_2	2.87 dd, 13.8,6.4	^{1}Tyr-2,5,5’	^{1}Tyr-2,3’	^{1}Tyr-2,3’,5,5’
		2.74 dd, 13.8,7.6		^{1}Tyr-2,3	^{1}Tyr-2,3,5,5’
4	126.9 C		^{1}Tyr-2,3,3’,6,6’		
5,5’	130.3 CH	6.98 d, 8.3	^{1}Tyr-3,3’,5’,5,6,6’	^{1}Tyr-6,6’	^{1}Tyr-2,3,3’,6,6’, *N*MeLeu-*N*Me
6,6’	115.1 CH	6.61 d, 8.3	^{1}Tyr-5,5’,6’,6,7-OH	^{1}Tyr-5,5’	^{1}Tyr-5,5’,7-OH, *N*MeLeu-*N*Me
7	156.2 C		^{1}Tyr-5,5’,6,6’,7-OH		
7-OH		9.24 s			^{1}Tyr-6,6’
*N*MeLeu 1	168.3 C		*N*MeLeu-2,3,3’		

2	51.6 CH	5.24 dd, 7.8,6.6	*N*MeLeu-3,3’,*N*Me	*N*MeLeu-3,3’	*N*MeLeu-3,3’,5,6,*N*Me, Pro-5,5’
2-*N*CH_3	30.1 CH_3	2.86 s	*N*MeLeu-2		*N*MeLeu-2,3’,4
3	37.2 CH_2	1.53 dt, 6.6,6.0	*N*MeLeu-2,4,5,6	*N*MeLeu-2,3’,4	*N*MeLeu-2,5,6
		1.36 m		*N*MeLeu-2,3,4	*N*MeLeu-2,5,6,*N*Me
4	24.2 CH	1.39 m	*N*MeLeu-2,3,3’,5,6	*N*MeLeu-3,3’,5,6	*N*MeLeu-*N*Me
5	22.4 CH_3	0.82 d, 6.2	*N*MeLeu-3,3’,4,6	*N*MeLeu-4	*N*MeLeu-2,3,3’
6	22.8 CH_3	0.85 d, 6.2	*N*MeLeu-3,3’,4,5	*N*MeLeu-4	*N*MeLeu-2,3,3’
Pro 1	173.4 C		Pro-2,3,3’		
2	58.8 CH	4.17 dd, 8.9,3.8	Pro-3,4,4’	Pro-3,3’	Pro-3,4’
3	28.8 CH_2	2.10 m	Pro-2,4,4’,5,5’	Pro-2,3’,4,4’	Pro-2,3’,4’
		1.82 m		Pro-2,3,4,4’	Pro-3
4	24.6 CH_2	1.86 m	Pro-2,3,3’,5,5’	Pro-3,3’,5,5’	Pro-3,4’
		1.81 m		Pro-3,3’,5,5’	Pro-2,4
5	46.5 CH_2	3.40 dt, 9.9,5.2		Pro-4,4’,5’	Pro-4,4’,5’
		3.24 dt, 9.9,6.9		Pro-4,4’,5	Pro-4,4’,5

S48. ^{1}H NMR Spectrum of Microginin KR604 (**5**) in DMSO-d_6

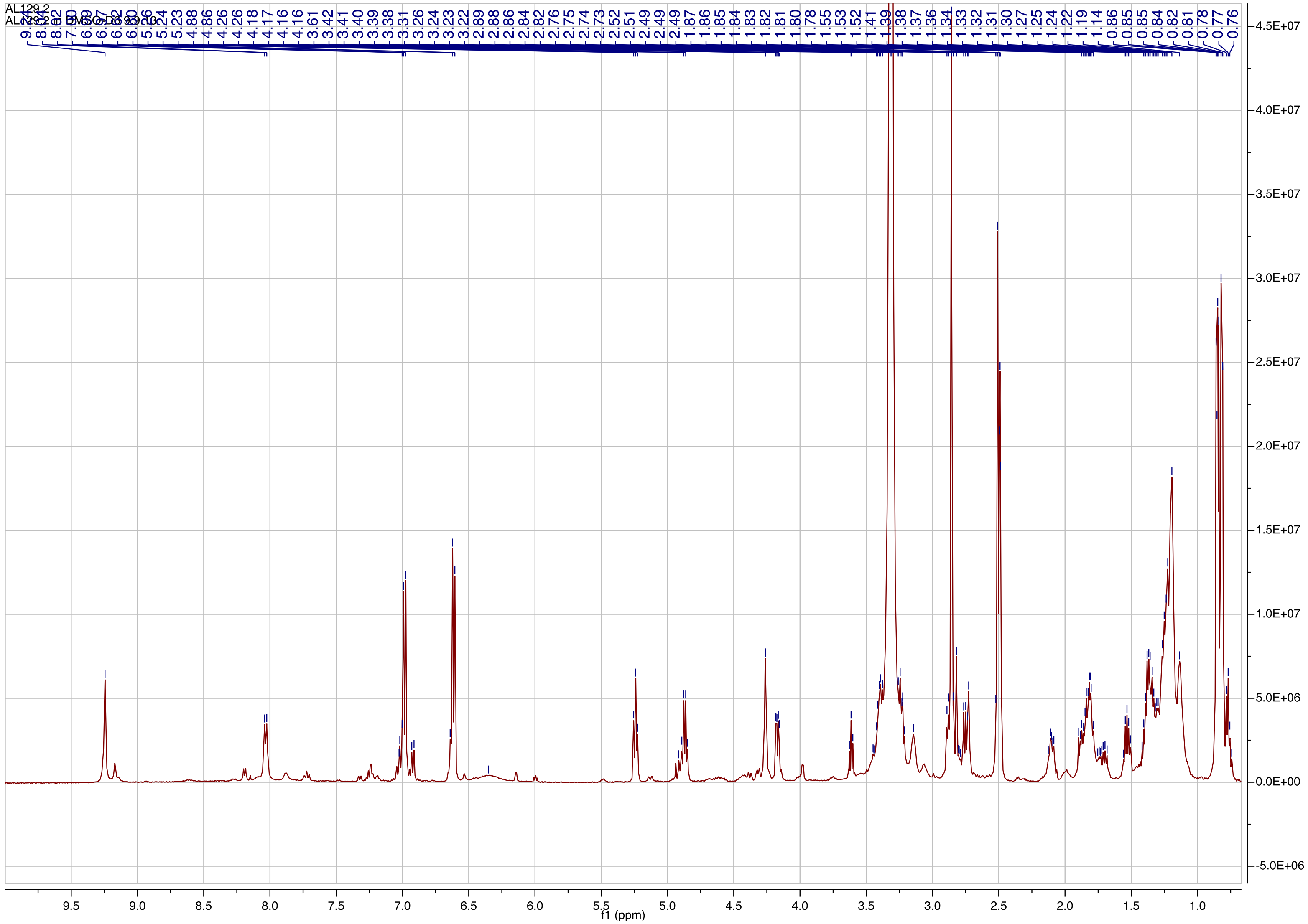

S49. ^{13}C NMR Spectrum of Microginin KR604 (**5**) in DMSO-d_6

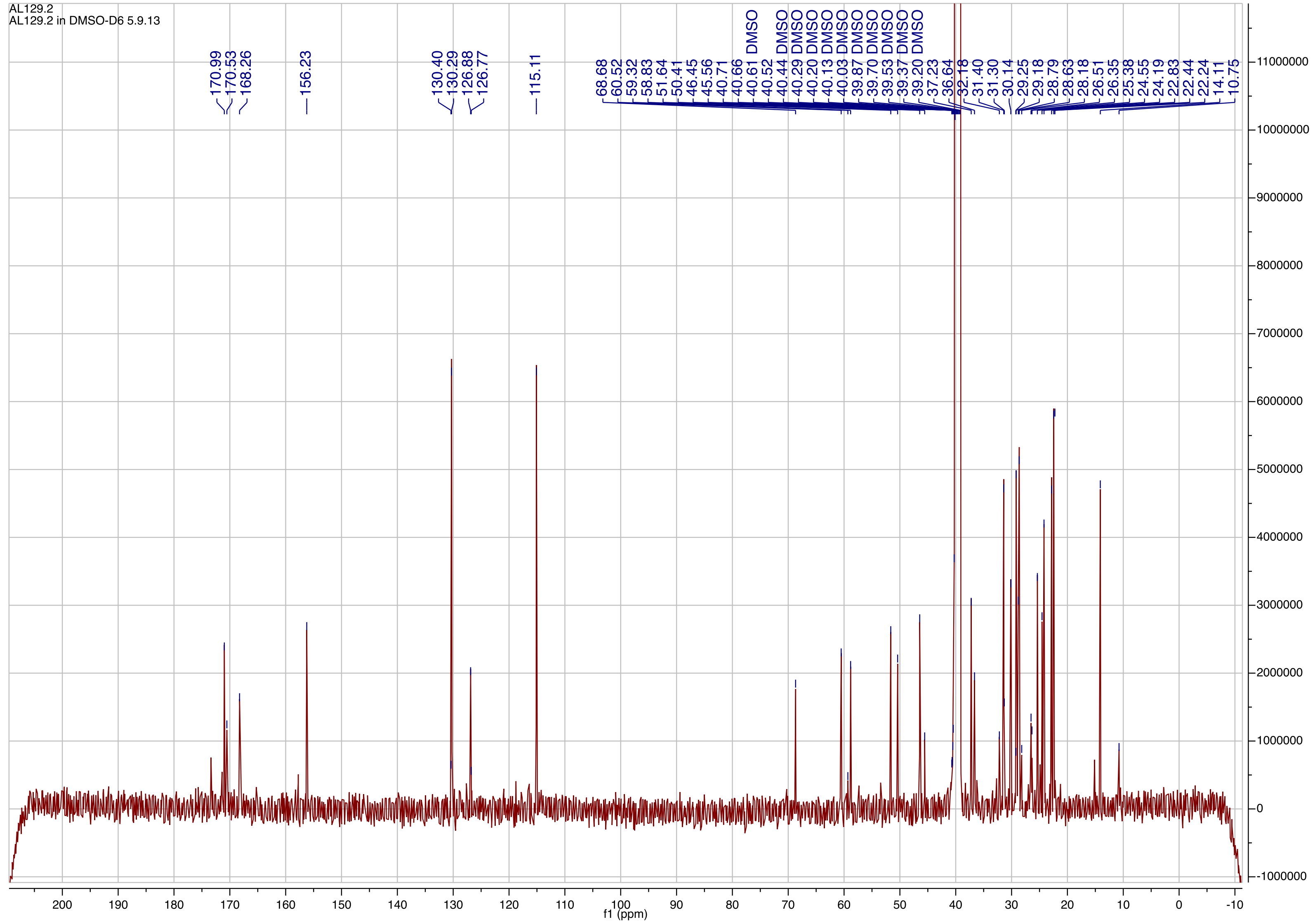

S50. HSQC Spectrum Microginin KR604 (**5**) in DMSO-d_6

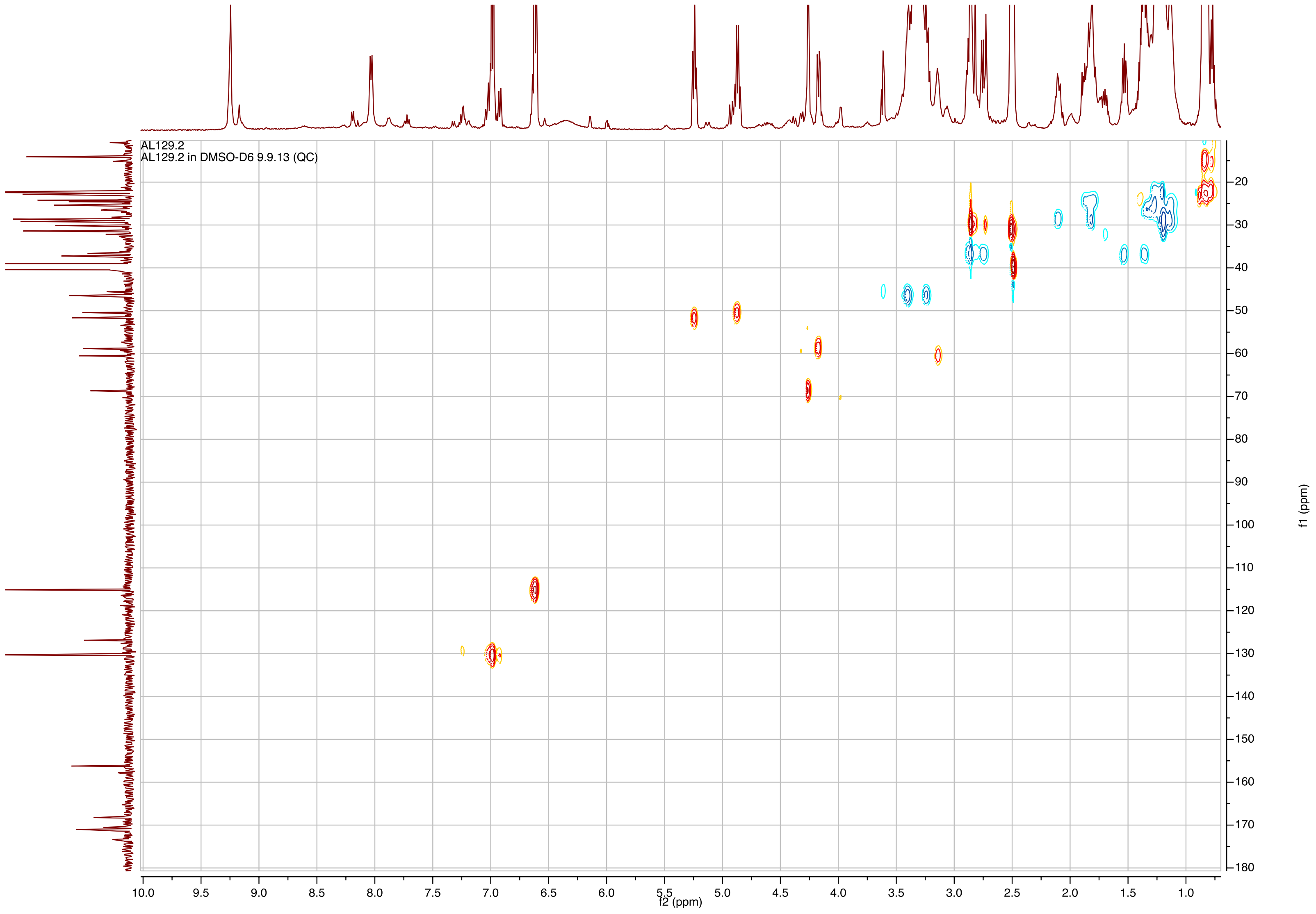

S51. HMBC Spectrum of Microginin KR604 (**5**) in DMSO-d_6

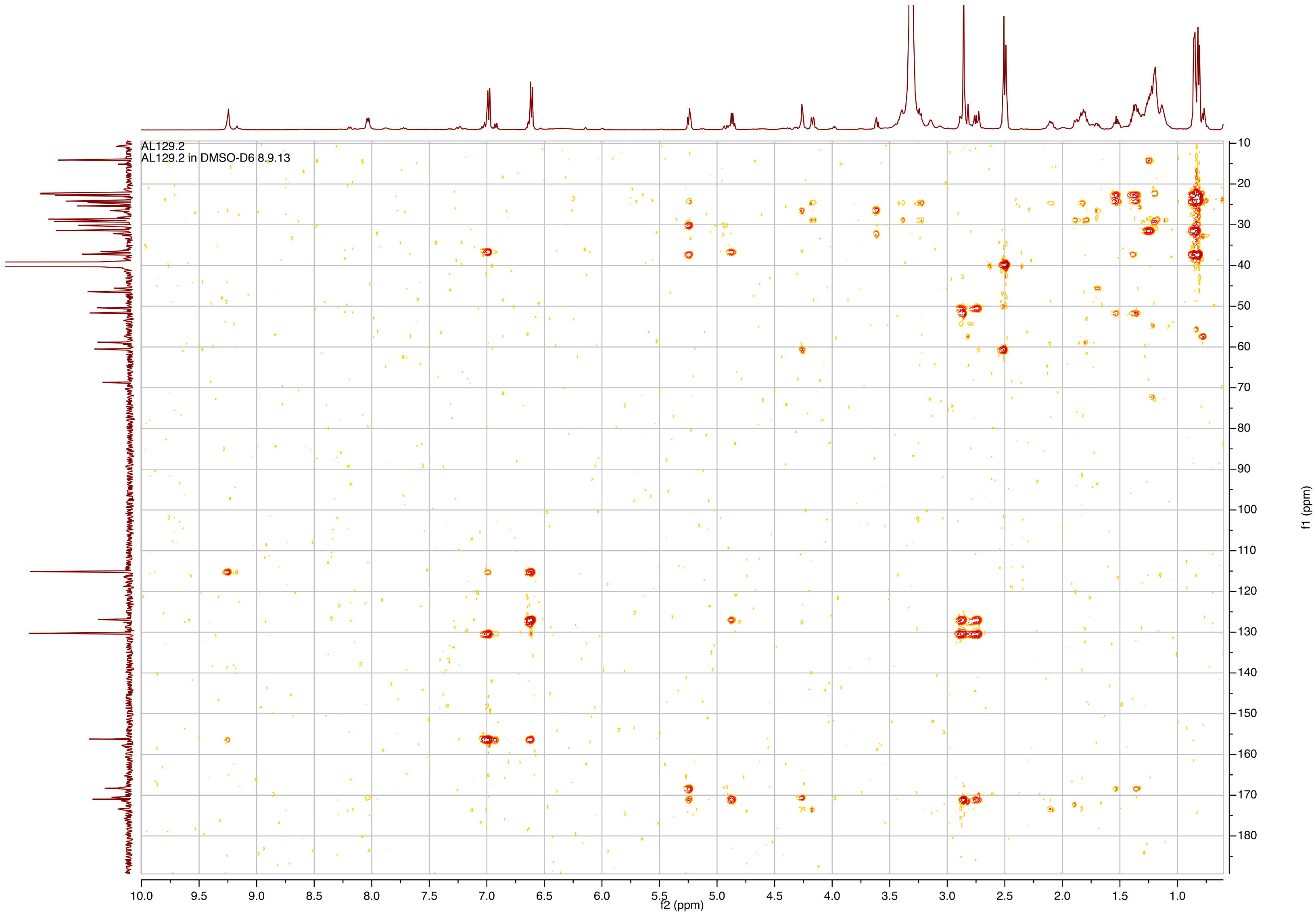

S52. COSY Spectrum of Microginin KR604 (**5**) in DMSO-d_6

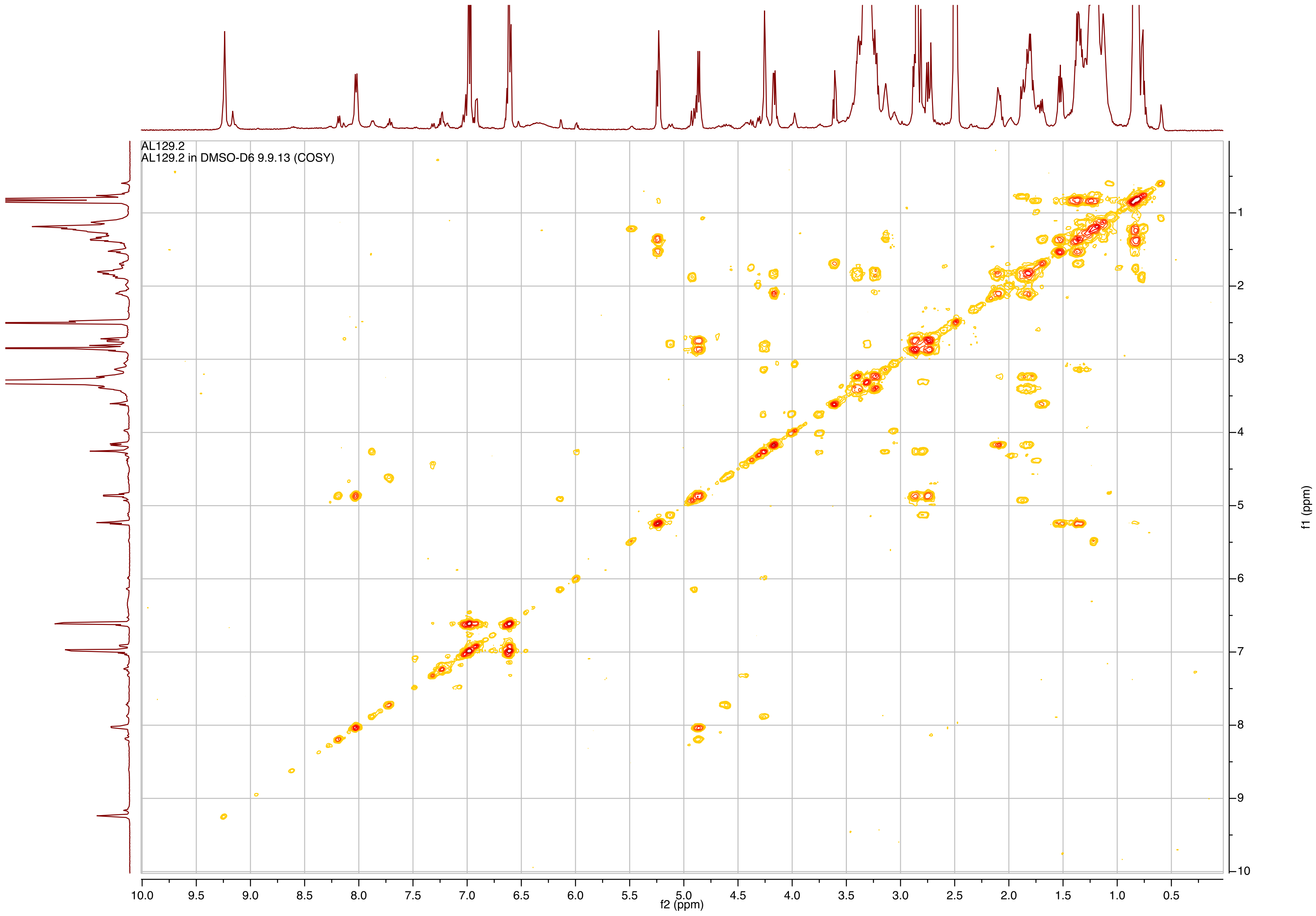

S53. TOCSY Spectrum of Microginin KR604 (**5**) in DMSO-d_6

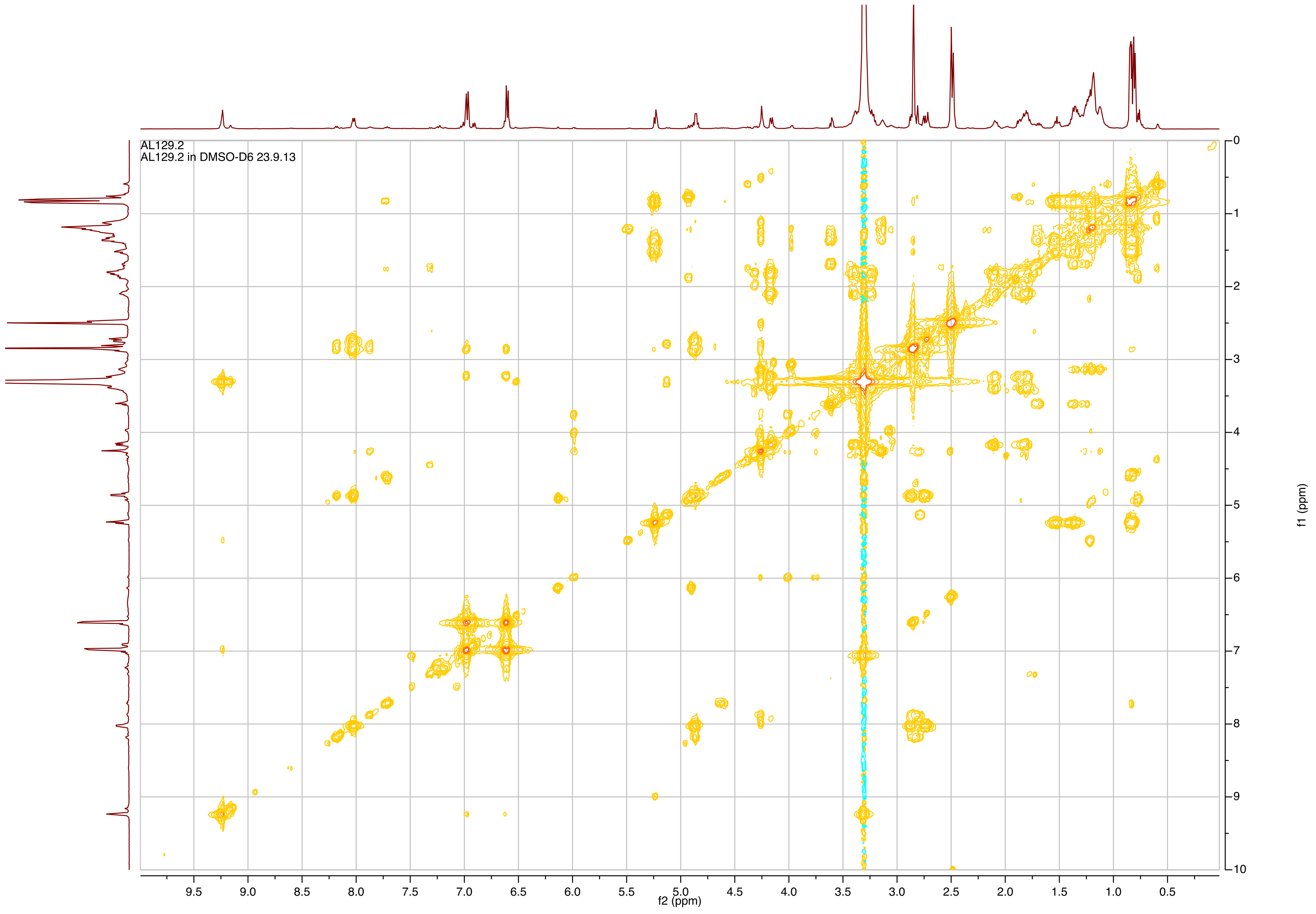

S54. ROESY Spectrum of Microginin KR604 (**5**) in DMSO-d_6

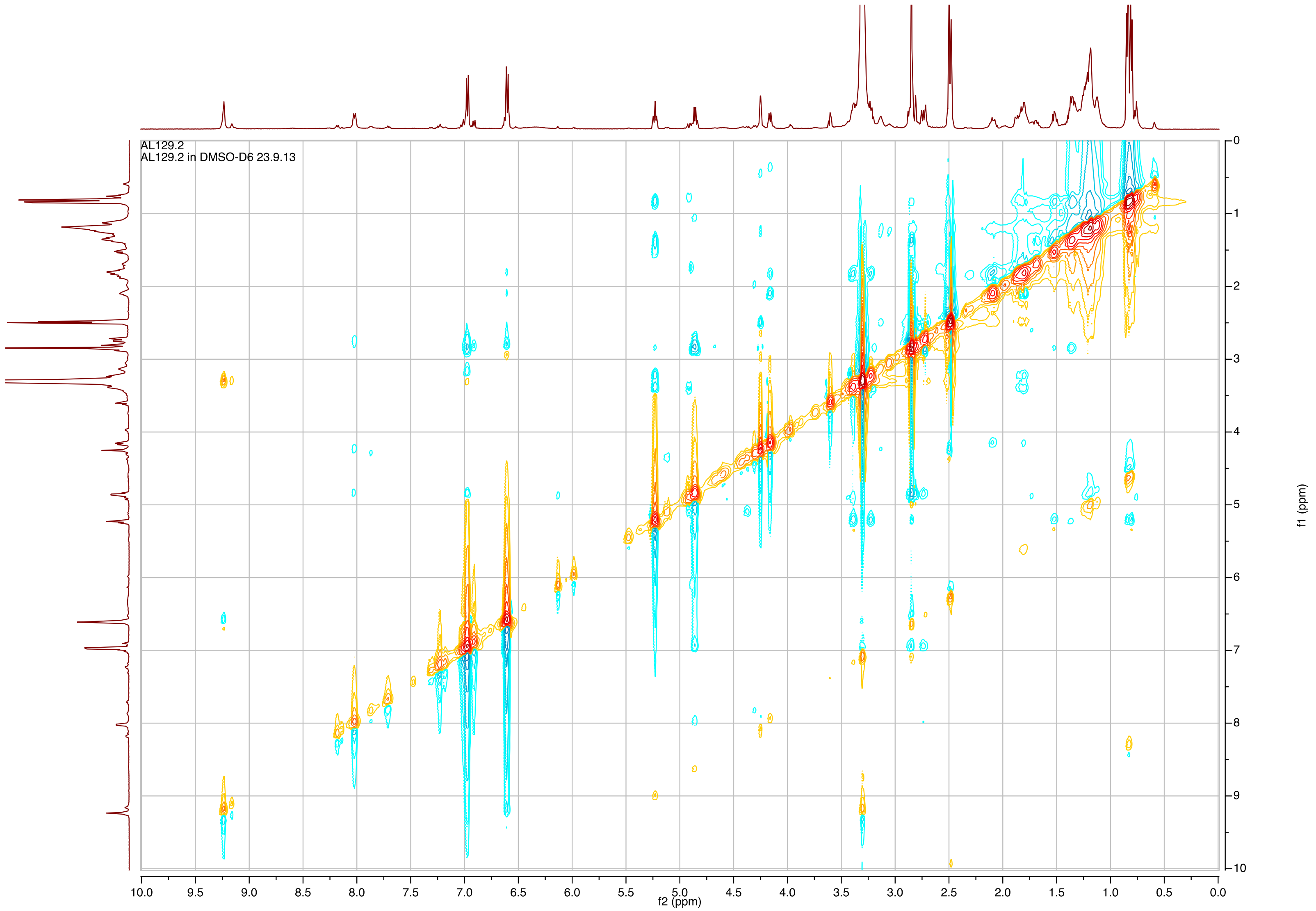

S55. HR ESI MS data of Microginin KR604 (**5**)

Elemental Composition Report Page 1

Single Mass Analysis
Tolerance = 3.0 PPM / DBE: min = -1.5, max = 50.0
Element prediction: Off
Number of isotope peaks used for i-FIT = 2

Monoisotopic Mass, Even Electron Ions
302 formula(e) evaluated with 4 results within limits (up to 50 closest results for each mass)
Elements Used:
C: 28-38 H: 45-60 N: 0-10 O: 0-15 23Na: 0-1
AL129.2 Anat Iodin
carmeli687 50 (1.913) Cm (50:55) 1: TOF MS ES- 1.44e+005

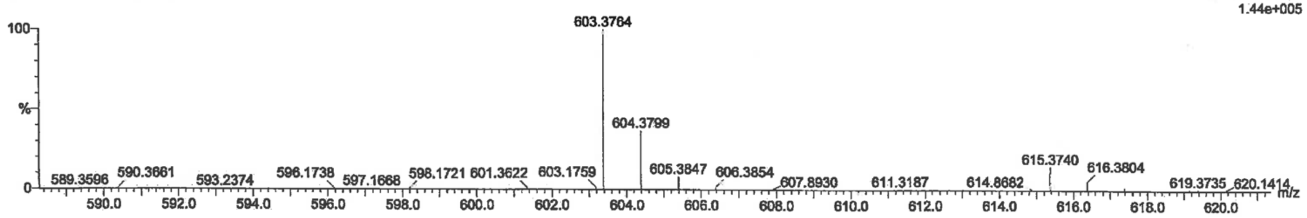

Minimum:				-1.5			
Maximum:		10.0	3.0	50.0			
Mass	Calc. Mass	mDa	PPM	DBE	i-FIT	i-FIT (Norm)	Formula
603.3764	603.3758	0.6	1.0	9.5	126.9	0.1	C32 H51 N4 O7
	603.3771	-0.7	-1.2	14.5	130.1	3.3	C33 H47 N8 O3
	603.3774	-1.0	-1.7	10.5	130.5	3.8	C35 H52 N2 O5 23Na
	603.3747	1.7	2.8	11.5	129.4	2.6	C31 H48 N8 O3 23Na

S56. **Table S7**. NMR Data (500/125 MHz) of Microginin KR638 (**6**) in DMSO-d_6

Position	δ_C	δ_H Multiplicity, J (Hz)	HMBC correlations	COSY correlations	NOESY correlations
Ahda 1	170.1 C		Ahda-2, [1]Tyr-NH		
2	68.4 CH	4.29 brs		Ahda-2-OH,3	Ahda-3,3-NCH$_3$, [1]Tyr-NH
2-OH		6.41 brs		Ahda-2	
3	60.2 CH	3.24 m	Ahda-2,3-NCH$_3$	Ahda-2,4,4'	Ahda-2,5,5',3-NCH$_3$, [1]Tyr-NH
3-NH		7.24 brs			
3-NCH$_3$	30.8 CH$_3$	2.55 brs			Ahda-2,3
4	26.1 CH$_2$	1.41 m	Ahda-2	Ahda-3,4',5,5'	
		1.33 m		Ahda-3,4,5,5'	
5	25.2 CH$_2$	1.38 m	Ahda-4,4',6,7	Ahda-4,4',5',6	Adha-3
		1.14 m		Ahda-4,4',5,6	Adha-3
6	29.1 CH$_2$	1.15 m	Ahda-7	Ahda-6	
7	28.2 CH$_2$	1.22 m	Ahda-6,8,9		
8	26.4 CH$_2$	1.34 tt. 7.7,7.4	Ahda-9,10	Ahda-7',9	Ahda-10
9	32.2 CH$_2$	1.68 tt, 7.4,6.8	Ahda-8,10	Ahda-8,10	Ahda-10
10	45.5 CH$_2$	3.60 t, 6.8	Ahda-8,9	Ahda-9	Ahda-8,9
[1]Tyr 1	171.0 C		[1]Tyr-2,3,3', NMeLeu-2, NCH$_3$		
2	50.5 CH	4.86 ddd, 8.2,7.7,6.3	[1]Tyr-3,3',NH	[1]Tyr-2-NH,3,3'	[1]Tyr-2-NH,3,3',5,5', NMeLeu-NMe
2-NH		8.09 d, 8.2		[1]Tyr-2	[1]Tyr-2,3,3', Ahda-2,3
3	36.5 CH$_2$	2.87 dd, 14.3,6.3	[1]Tyr-2,5,5'	[1]Tyr-2,3'	[1]Tyr-2,3',5,5'
		2.75 dd, 14.3,7.7		[1]Tyr-2,3	[1]Tyr-2,3,5,5'
4	126.9 C		[1]Tyr-3,3',6,6'		
5,5'	130.3 CH	6.99 d, 8.2	[1]Tyr-3,3',5',5	[1]Tyr-6,6'	[1]Tyr-2,3,3',6,6, NMeLeu-NMe
6,6'	115.0 CH	6.62 d, 8.2	[1]Tyr-5,5',6',6,7-OH	[1]Tyr-5,5'	[1]Tyr-5,5',7-OH, NMeLeu-NMe
7	156.0 C		[1]Tyr-5,5',6,6',7-OH		
7-OH		9.25 s			[1]Tyr-6,6'
NMeLeu 1	168.3 C		NMeLeu-2,3,3'		

2	51.7 CH	5.24 dd, 7.6,6.8	*N*MeLeu-3,3',*N*Me	*N*MeLeu-3,3'	*N*MeLeu-3,3',5,6,*N*Me, Pro-5,5'
2-NCH_3	30.2 CH_3	2.87 s	*N*MeLeu-2		*N*MeLeu-2,3,3'
3	37.2 CH_2	1.53 m	*N*MeLeu-2,4,5,6	*N*MeLeu-2,3',4	*N*MeLeu-2,5,6,*N*Me
		1.38 m		*N*MeLeu-2,3,4	*N*MeLeu-2,5,6,*N*Me
4	24.2 CH	1.39 m	*N*MeLeu-2,3,3',5,6	*N*MeLeu-3,3',5,6	*N*MeLeu-*N*Me
5	22.5 CH_3	0.82 d, 6.2	*N*MeLeu-3,3',4,6	*N*MeLeu-4	*N*MeLeu-2,3,3'
6	22.9 CH_3	0.86 d, 6.2	*N*MeLeu-3,3',4,5	*N*MeLeu-4	*N*MeLeu-2,3,3'
Pro 1	173.2 C		Pro-2,3,3'		
2	58.8 CH	4.18 dd, 8.9,3.8	Pro-3,4,4',5'	Pro-3,3'	Pro-3,4'
3	28.8 CH_2	2.11 m	Pro-2,4,4',5,5'	Pro-2,3',4,4'	Pro-2,3',4'
		1.82 m		Pro-2,3,4,4'	Pro-3
4	24.6 CH_2	1.86 m	Pro-2,3,3',5,5'	Pro-3,3',5,5'	Pro-2
		1.81 m		Pro-3,3',5,5'	Pro-3
5	46.5 CH_2	3.41 m	Pro-2,3'	Pro-4,4',5'	Pro-4,4',5'
		3.26 m		Pro-4,4',5	Pro-2,3,4,4',5

S58. ^{1}H NMR Spectrum of Microginin KR638 (**6**) in DMSO-d_6

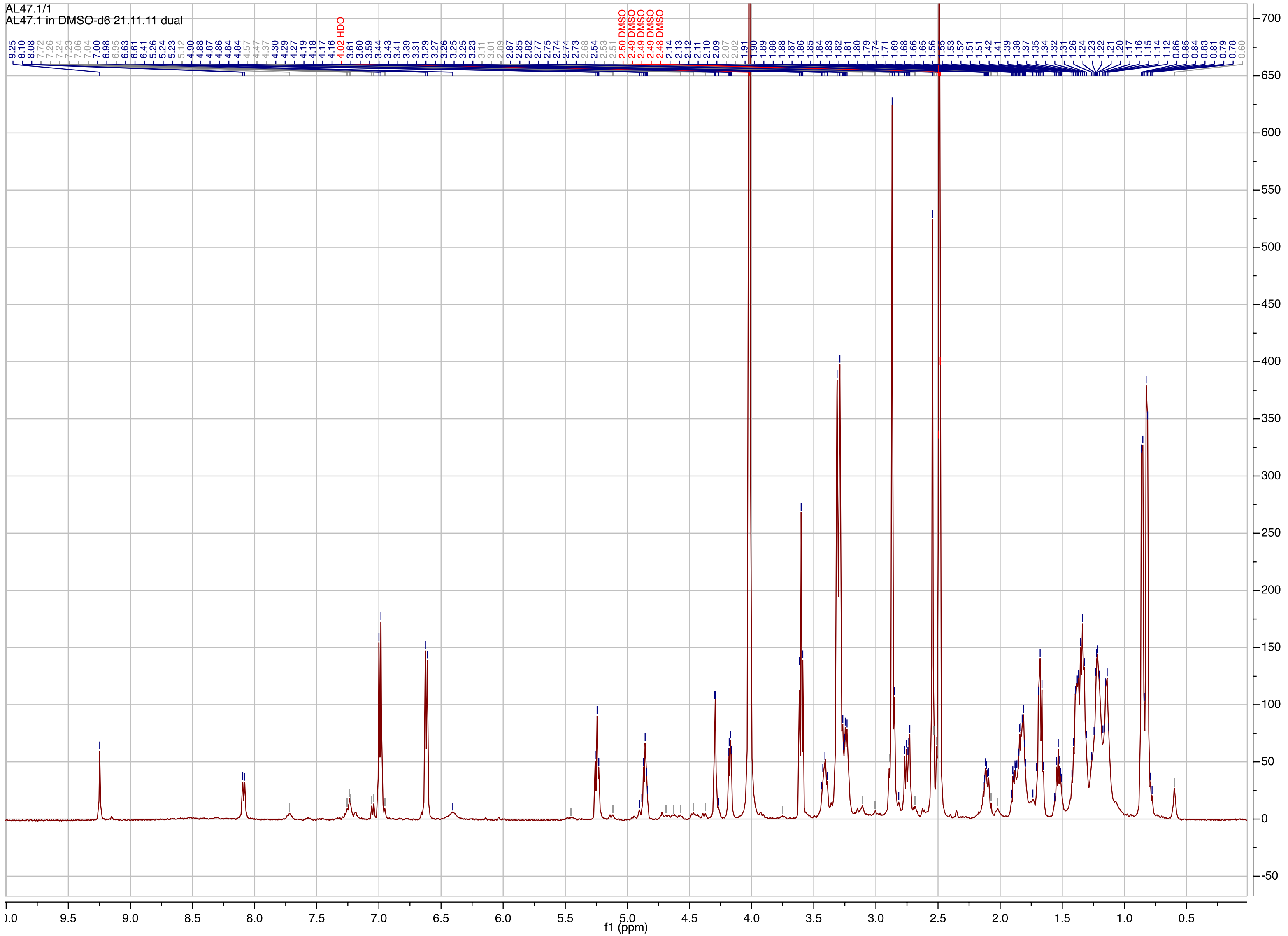

S59. ^{13}C NMR Spectrum of Microginin KR638 (**6**) in DMSO-d_6

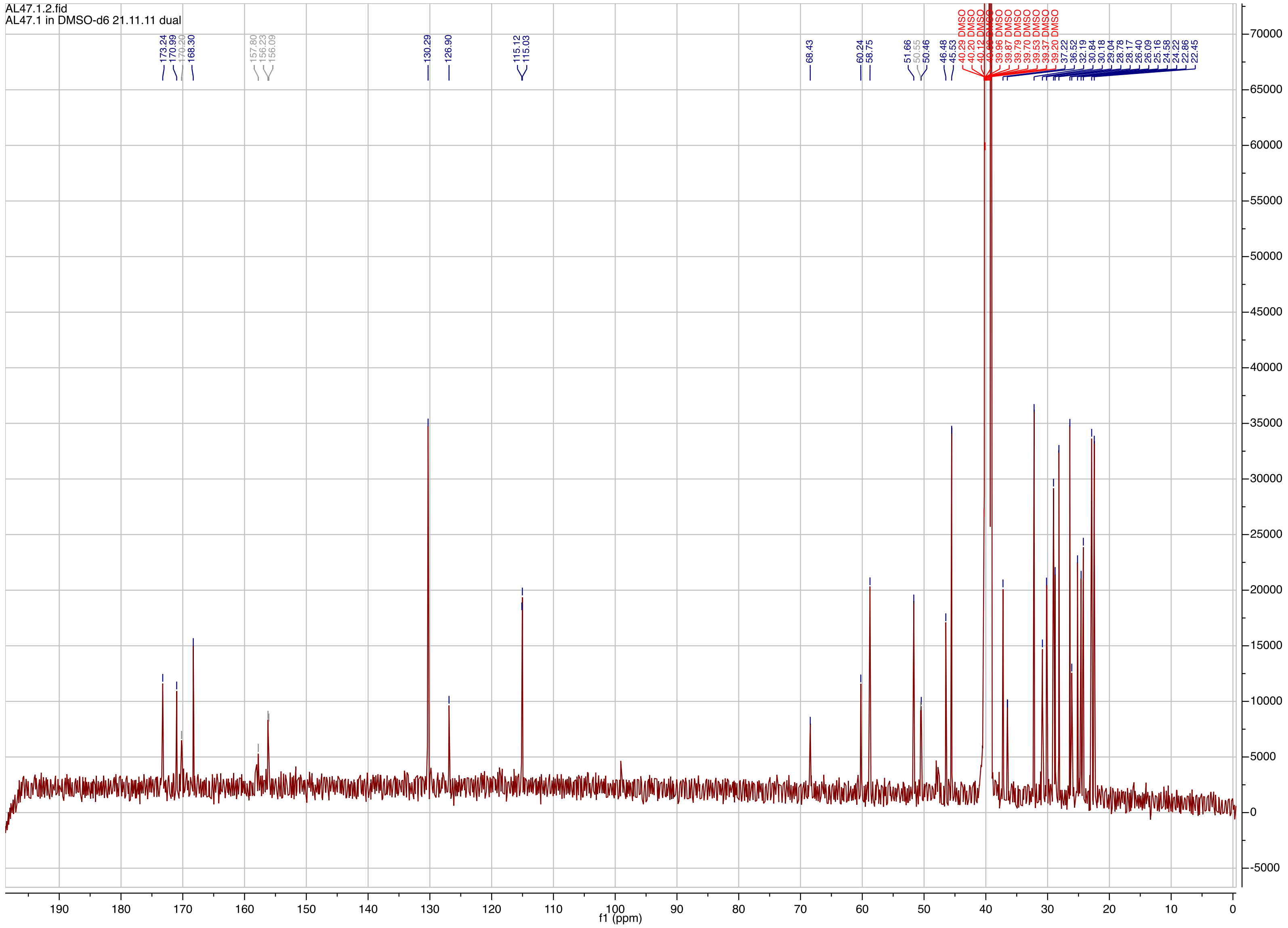

S60. HSQC Spectrum Microginin KR638 (**6**) in DMSO-d_6

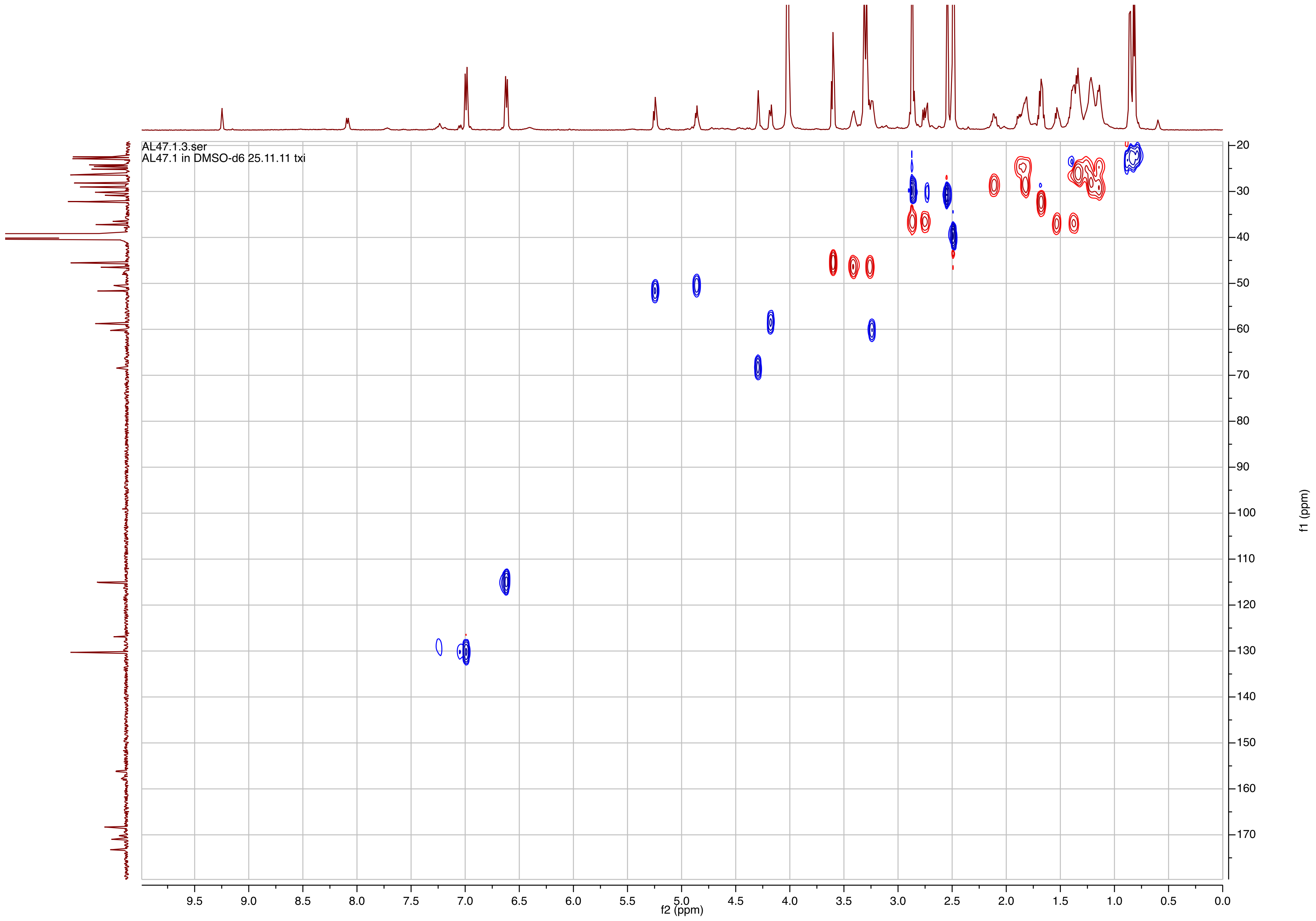

S61. HMBC Spectrum of Microginin KR638 (**6**) in DMSO-d_6

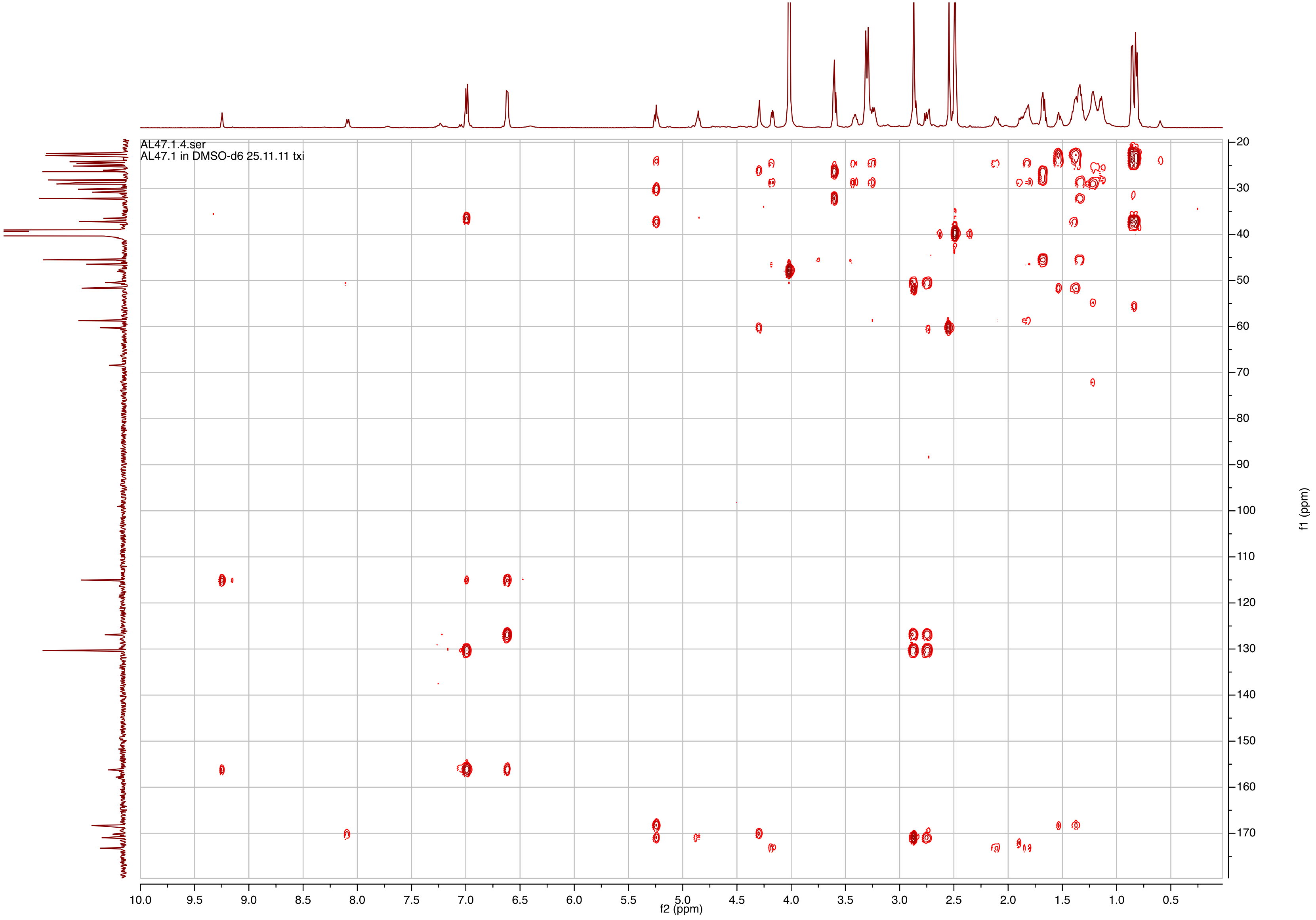

S62. COSY Spectrum of Microginin KR638 (**6**) in DMSO-d_6

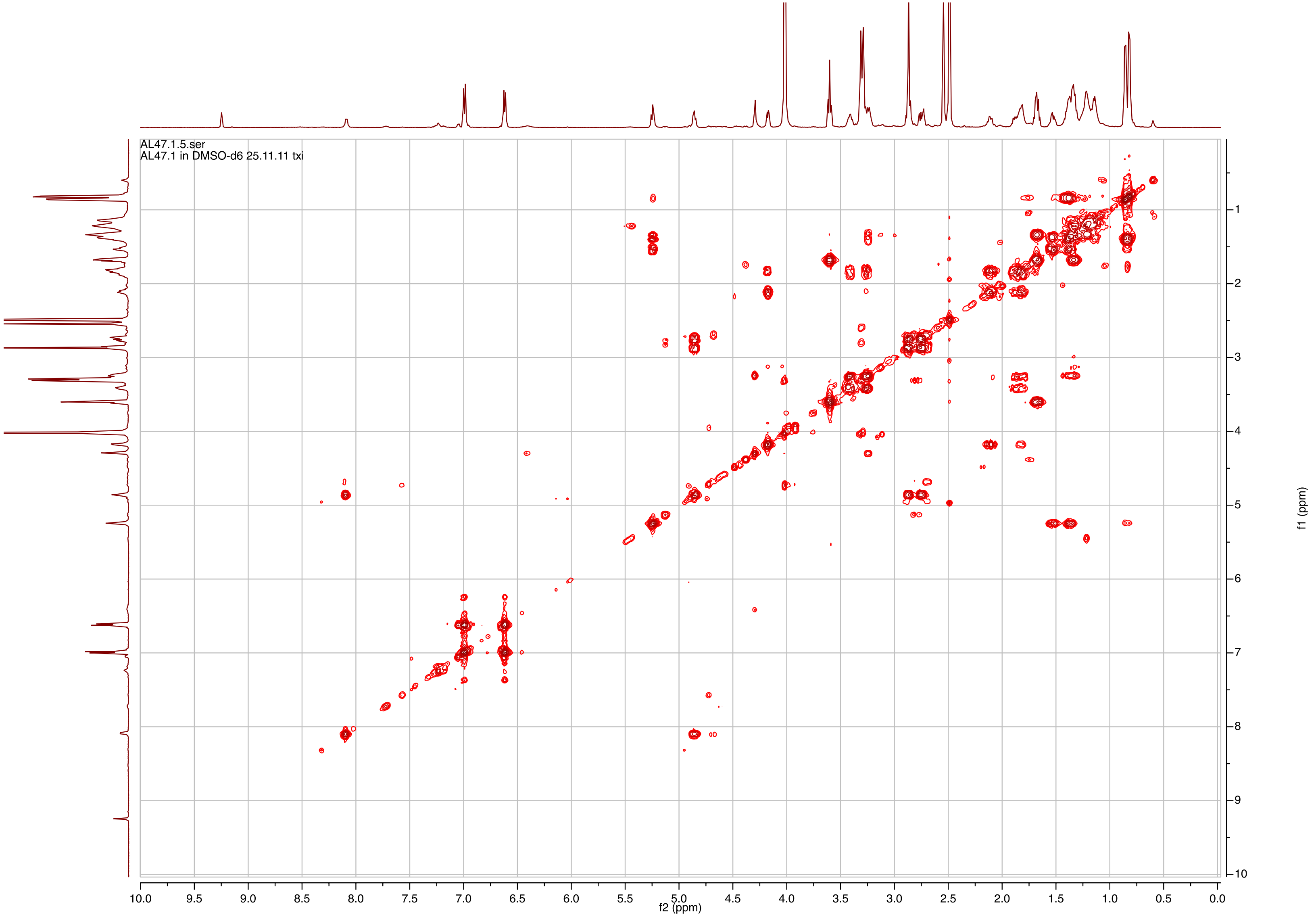

S63. TOCSY Spectrum of Microginin KR638 (**6**) in DMSO-d_6

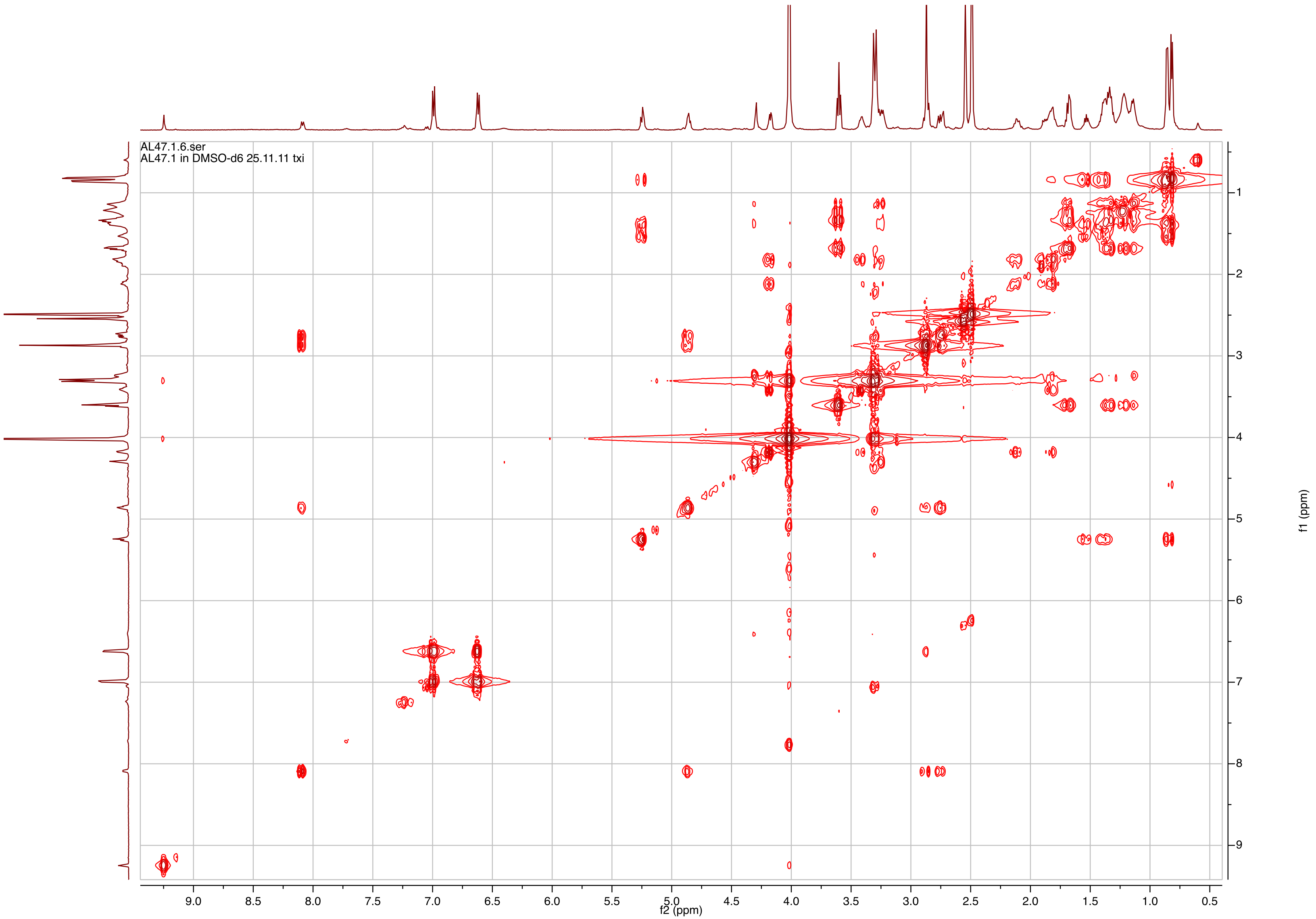

S64. ROESY Spectrum of Microginin KR638 (**6**) in DMSO-d_6

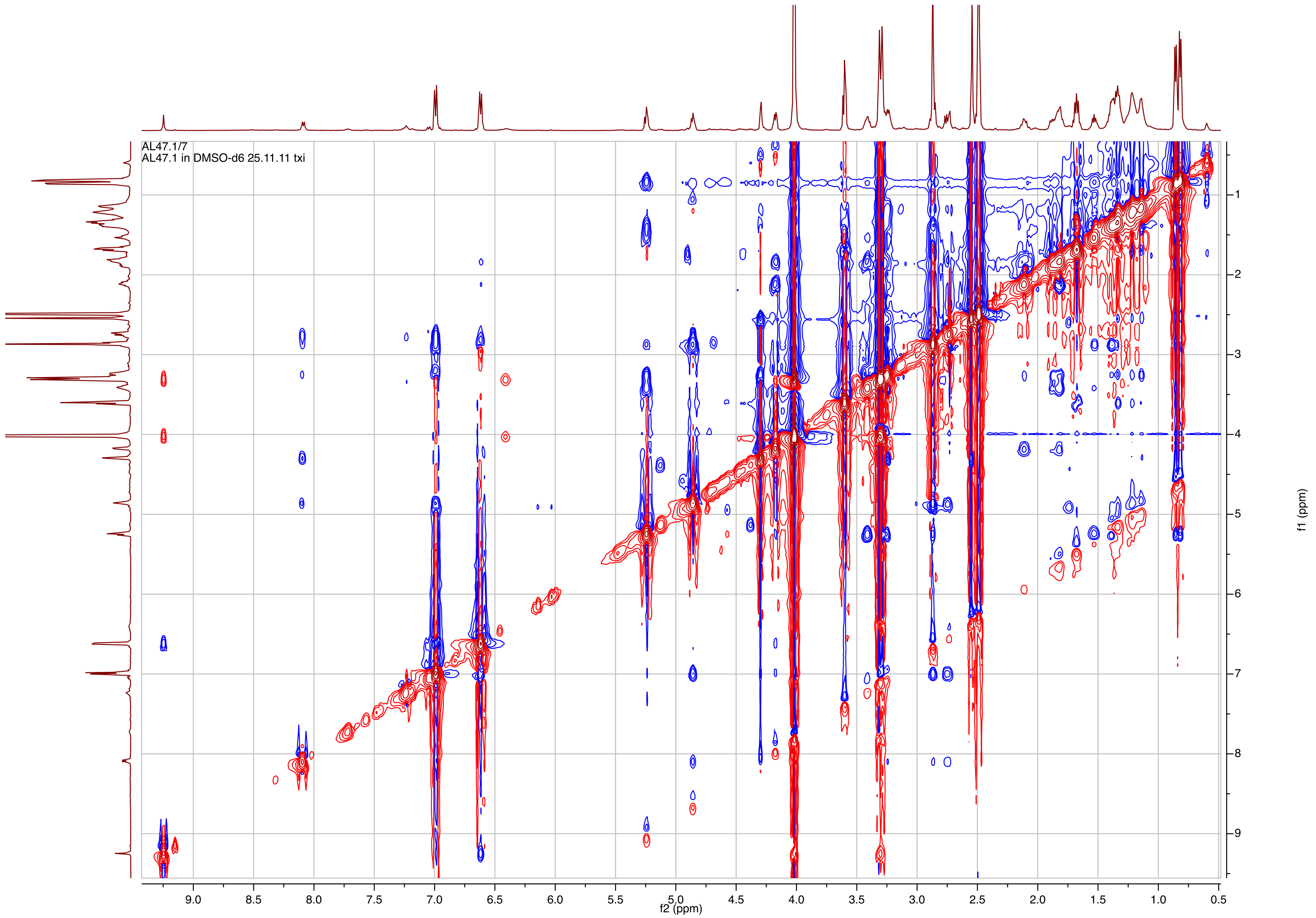

S65. HR ESI MS data of Microginin KR638 (**6**)

Elemental Composition Report

Single Mass Analysis
Tolerance = 20.0 PPM / DBE: min = -1.5, max = 50.0
Element prediction: Off
Number of isotope peaks used for i-FIT = 3

Monoisotopic Mass, Even Electron Ions
283 formula(e) evaluated with 31 results within limits (up to 5 closest results for each mass)
Elements Used:
C: 28-35 H: 48-58 N: 0-10 O: 0-10 Na: 0-1 S: 0-1 Cl: 1-1
AI47.1
carmeli404d 166 (7.294) Cm (159:166)

Anat Iodin

1: TOF MS ES+
2.65e+005

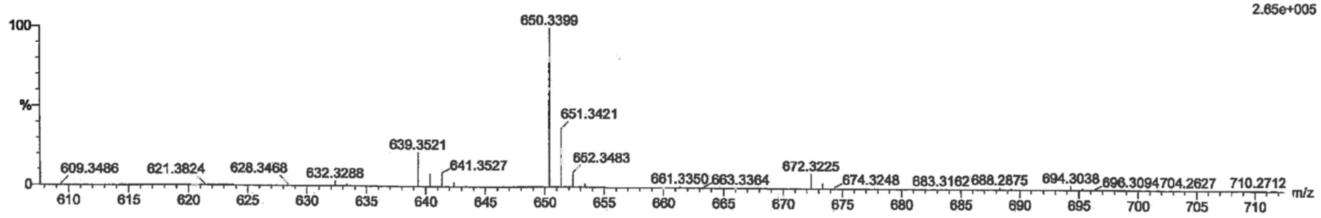

Minimum:				-1.5			
Maximum:		5.0	20.0	50.0			

Mass	Calc. Mass	mDa	PPM	DBE	i-FIT	i-FIT (Norm)	Formula
639.3521	639.3525	-0.4	-0.6	8.5	276.9	3.9	C32 H52 N4 O7 Cl
	639.3514	0.7	1.1	10.5	277.7	4.7	C31 H49 N8 O3 Na Cl
	639.3538	-1.7	-2.7	13.5	280.5	7.5	C33 H48 N8 O3 Cl
	639.3541	-2.0	-3.1	9.5	280.1	7.1	C35 H53 N2 O5 Na Cl
	639.3500	2.1	3.3	5.5	273.0	0.0	C30 H53 N4 O7 Na Cl

S66. **Table S8**. NMR Data (500/125 MHz) of Microginin KR781 (**7**) in DMSO-*d*$_6$

Position	δ_C	δ_H Multiplicity, *J* (Hz)	HMBC correlations	COSY correlations	NOESY correlations
Ahda 1	170.2 C		Ahda-2, ^{1}Tyr-2,NH		
2	68.5 CH	4.30 brs		Ahda-2-OH	Ahda-2-OH,3,3-*N*CH$_3$, ^{1}Tyr-NH
2-OH		6.35 brm		Ahda-2	Ahda-2,3
3	60.3 CH	3.22 brm	Ahda-2,3-*N*CH$_3$	Ahda-4,	Ahda-2,2-OH
3-NH		8.43 brm		3-*N*CH$_3$(TOCSY)	Ahda-3-*N*CH$_3$
3-*N*CH$_3$	30.8 CH$_3$	2.53 brs		Ahda-3-NH(TOCSY)	Ahda-2,2-OH,3,3-NH
4	26.1 CH$_2$	1.36 m	Ahda-2	Ahda-3,4’,5,5’	
		1.32 m		Ahda-4,5,5’	
5	25.3 CH$_2$	1.24 m		Ahda-4,4’,5’,6	
		1.13 m		Ahda-4,4’,5,6	
6	29.2 CH$_2$	1.17 m	Ahda-5,5’,7,7’	Ahda-6	
		1.13 m			
7	28.6 CH$_2$	1.20 m	Ahda-6,6’,8		
8	31.4 CH$_2$	1.20 m	Ahda-9,10	Ahda-9	
9	22.3 CH$_2$	1.25 m	Ahda-8,10	Ahda-8,10	
10	14.1 CH$_3$	0.84 t, 7.4	Ahda-9	Ahda-9	
^{1}Tyr 1	171.0 C		^{1}Tyr-2,3,3’, *N*MeLeu-2, *N*CH$_3$		
2	50.6 CH	4.86 ddd, 7.7,7.4,6.5	^{1}Tyr-3,3’	^{1}Tyr-2-NH,3,3’	^{1}Tyr-2-NH,3,3’,5,5’, *N*MeLeu-*N*Me
2-NH		8.05 d, 7.4		^{1}Tyr-2	^{1}Tyr-2,3,3’, Ahda-2
3	36.4 CH$_2$	2.87 m	^{1}Tyr-2,5,5’	^{1}Tyr-2,3’	^{1}Tyr-2,2-NH,3’,5,5’
		2.74 dd, 13.8,8.0		^{1}Tyr-2,3	^{1}Tyr-2,2-NH,3,5,5’
4	126.9 C		^{1}Tyr-2,3,3’,6,6’		
5,5’	130.3 CH	6.99 d, 8.3	^{1}Tyr-3,3’,5’,5	^{1}Tyr-6,6’	^{1}Tyr-2,3,3’,6,6’
6,6’	115.2 CH	6.62 d, 8.3	^{1}Tyr-5,5’,6’,6,7-OH	^{1}Tyr-5,5’	^{1}Tyr-5,5’,7-OH
7	156.2 C		^{1}Tyr-5,5’,6,6’,7-OH		
7-OH		9.25 s			^{1}Tyr-6,6’

*N*MeLeu 1	168.4 C		*N*MeLeu-2,3,3’		
2	51.9 CH	5.23 dd, 8.0,6.5	*N*MeLeu-3,3’,*N*Me	*N*MeLeu-3,3’	*N*MeLeu-3,3’,5,6,*N*Me, Pro-5,5’
2-*N*CH_3	30.2 CH_3	2.88 s	*N*MeLeu-2		*N*MeLeu-2, [1]Tyr-2
3	37.2 CH_2	1.47 m	*N*MeLeu-2,4,5,6	*N*MeLeu-2,3’,4	*N*MeLeu-2,5,6
		1.42 m		*N*MeLeu-2,3,4	*N*MeLeu-2,5,6
4	24.3 CH	1.38 m	*N*MeLeu-2,3,3’,5,6	*N*MeLeu-3,3’,5,6	*N*MeLeu-2
5	22.4 CH_3	0.81 d, 6.6	*N*MeLeu-3,3’,4,6	*N*MeLeu-4	*N*MeLeu-2,3,3’
6	23.0 CH_3	0.85 d, 6.6	*N*MeLeu-3,3’,4,5	*N*MeLeu-4	*N*MeLeu-2,3,3’
Pro 1	171.7 C		Pro-2, [2]Tyr-NH		
2	59.3 CH	4.29 m		Pro-3,3’	Pro-3,3’,5’
3	29.2 CH_2	1.95 m	Pro-4,4’	Pro-2,3’,4,4’	Pro-2
		1.73 m		Pro-2,3,4,4’	Pro-2
4	24.2 CH_2	1.80 m	Pro-3,5’	Pro-3,3’,5,5’	
		1.75 m		Pro-3,3’,5,5’	
5	46.7 CH_2	3.39 m		Pro-4,4’,5’	Pro-5’, *N*MeLeu-2
		3.17 m		Pro-4,4’,5	Pro-2,5, *N*MeLeu-2
[2]Tyr 1	172.2 C		[2]Tyr-2,3,3’, OCH_3		
2	54.3 CH	4.31 m	[2]Tyr-3,3’	[2]Tyr-3,3’,NH	[2]Tyr-3,3’,5,5’,NH
2-NH		8.15 d, 7.3		[2]Tyr-2	[2]Tyr-2,3,3’
3	36.1 CH_2	2.86 m	[2]Tyr-2,5,5’	[2]Tyr-2,3’	[2]Tyr-2,3’,5,5’,NH
		2.82 m		[2]Tyr-2,3	[2]Tyr-2,3,5,5’,NH
4	127.2 C		[2]Tyr-2,3,3’,6,6’		
5,5’	130.2 CH	7.00 d, 8.4	[2]Tyr-3,3’,5’,5	[2]Tyr-6,6’	[2]Tyr-2,3,3’,6,6’
6,6’	115.2 CH	6.64 d, 8.4	[2]Tyr-5,5’,6’,6,7-OH	[2]Tyr-5,5’	[2]Tyr-5,5’,7-OH
7	156.2 C		[2]Tyr-5,5’,6,6’,7-OH		
7-OH		9.23 s			[2]Tyr-6,6’
OCH_3	51.9 CH_3	3.53 s			

S68. ^{1}H NMR Spectrum of Microginin KR781 (**7**) in DMSO-d_6

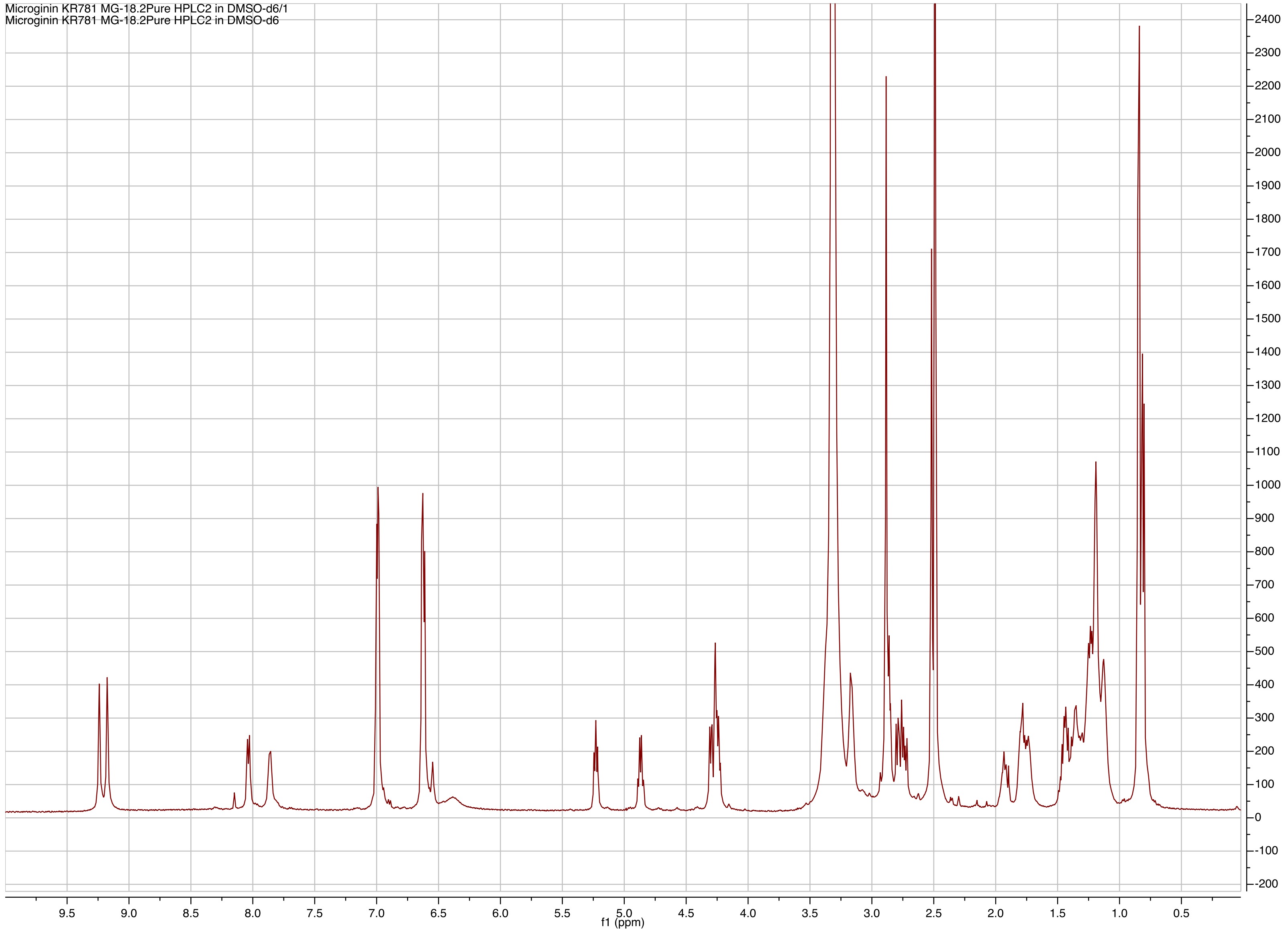

S69. ^{13}C NMR Spectrum of Microginin KR781 (**7**) in DMSO-*d*$_6$

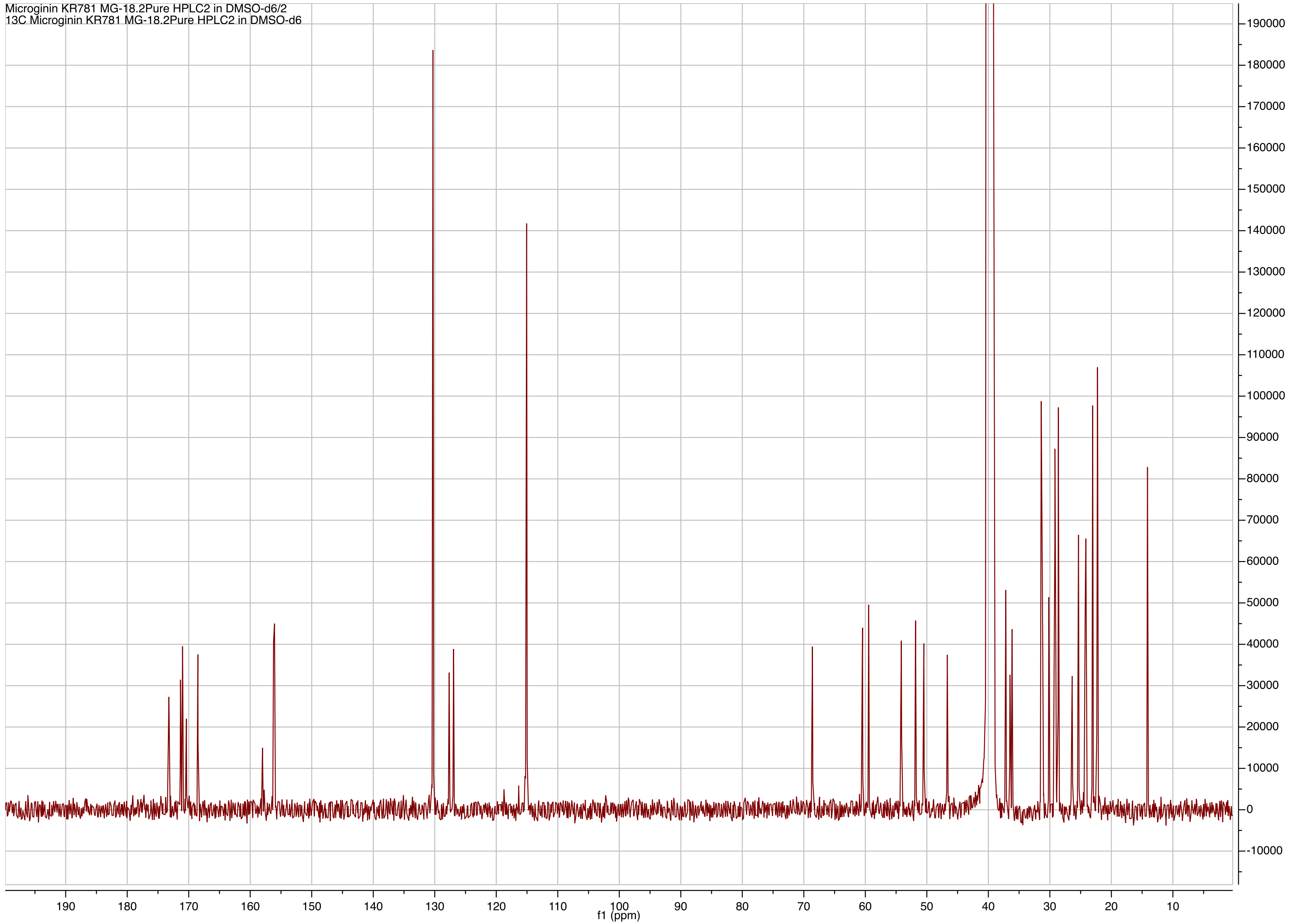

S70. HSQC Spectrum Microginin KR781 (**7**) in DMSO-d_6

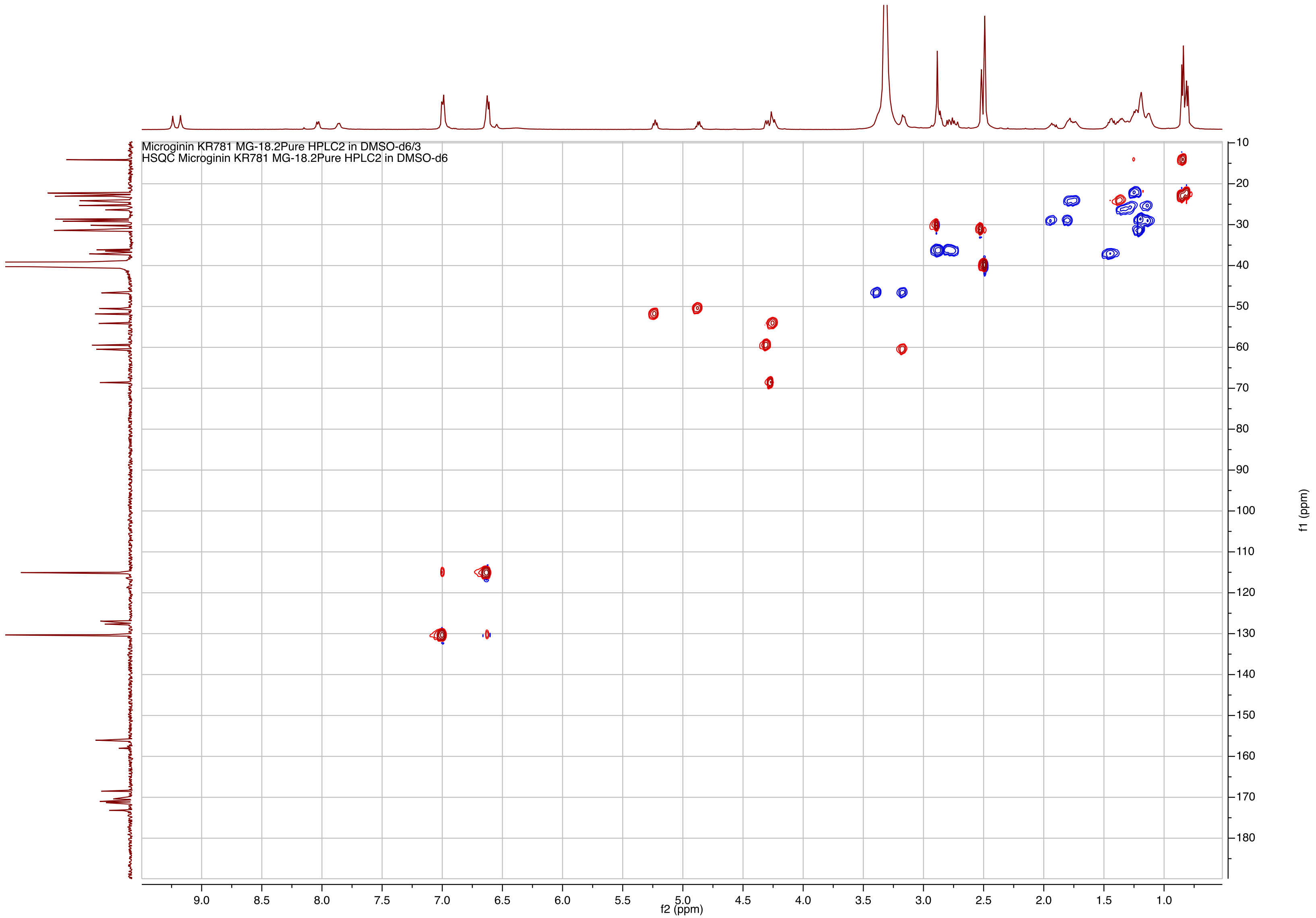

S71. HMBC Spectrum of Microginin KR781 (**7**) in DMSO-d_6

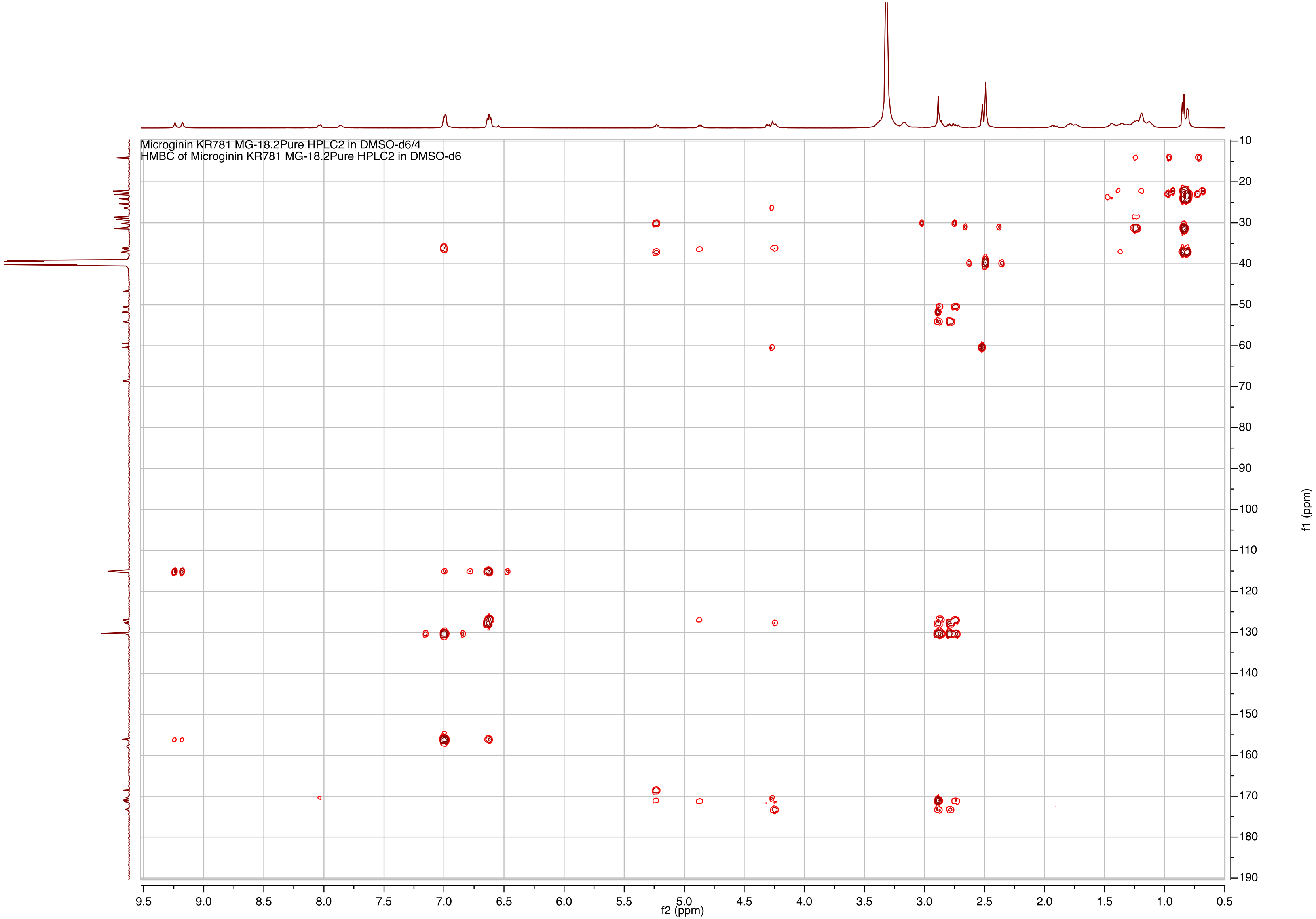

S72. COSY Spectrum of Microginin KR781 (**7**) in DMSO-d_6

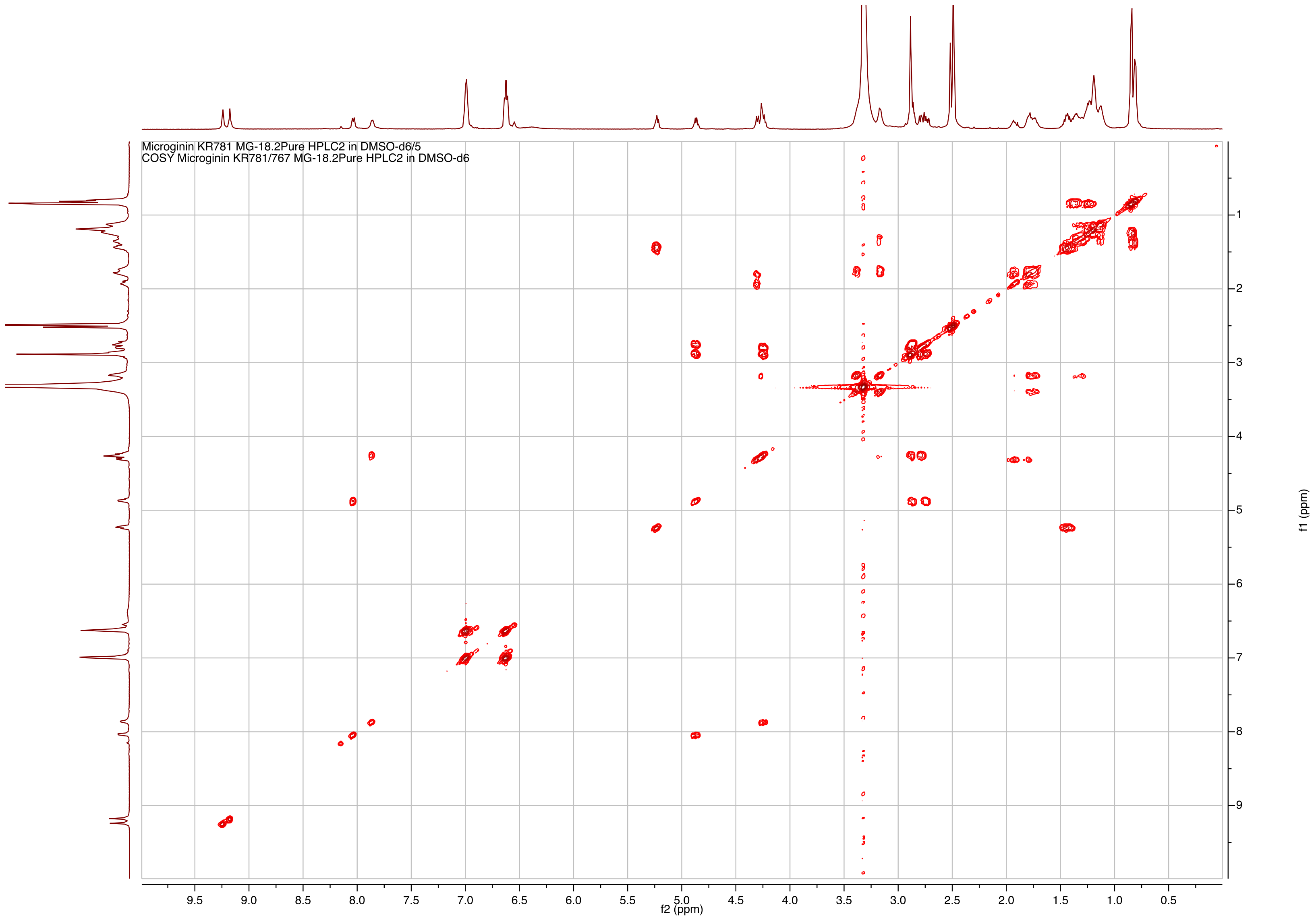

S73. TOCSY Spectrum of Microginin KR781 (**7**) in DMSO-d_6

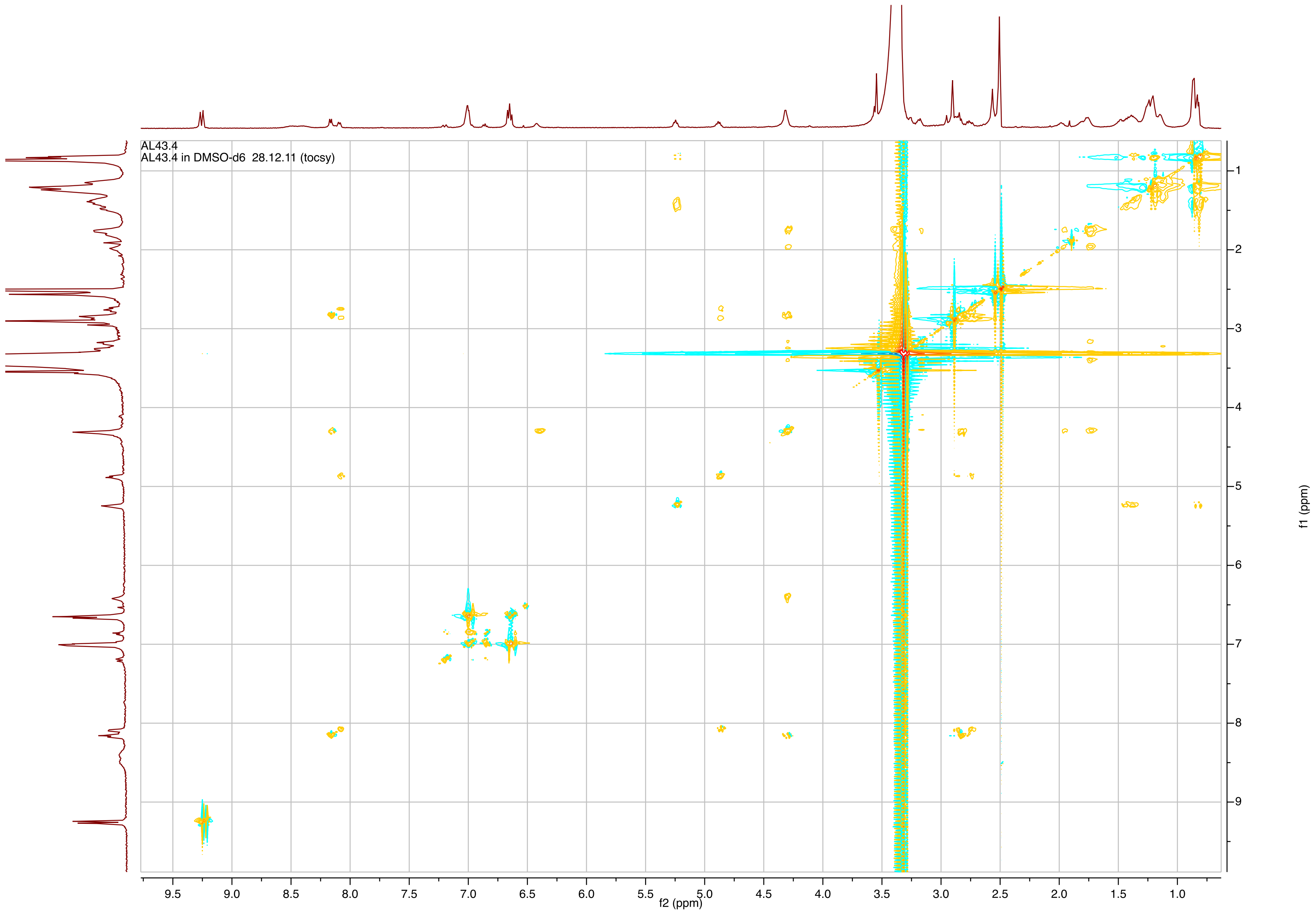

S74. ROESY Spectrum of Microginin KR781 (**7**) in DMSO-d_6

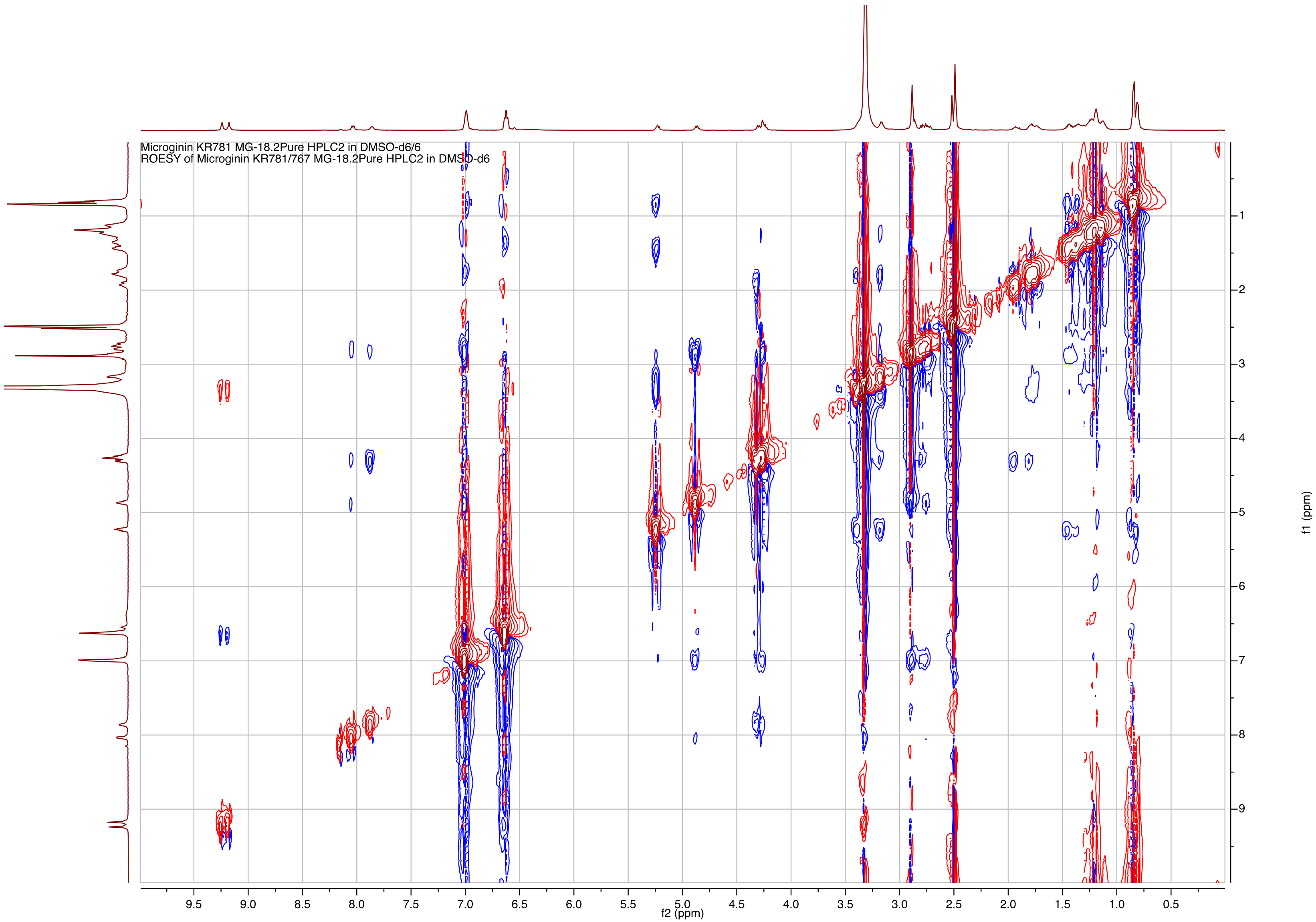

S75. HR ESI MS data of Microginin KR781 (**7**)

Elemental Composition Report Page 1

Single Mass Analysis
Tolerance = 2.0 PPM / DBE: min = -1.5, max = 50.0
Element prediction: Off
Number of isotope peaks used for i-FIT = 3

Monoisotopic Mass, Even Electron Ions
65 formula(e) evaluated with 1 results within limits (all results (up to 1000) for each mass)
Elements Used:
C: 40-45 H: 60-65 N: 0-10 O: 0-15
AL43.4
Carmeli411b 62 (2.380) Cm (62)

Anat Lodin

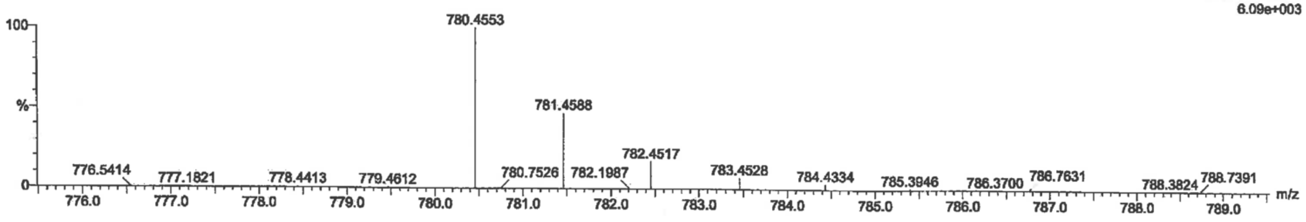

		mDa	PPM	DBE			
Minimum:				-1.5			
Maximum:		5.0	2.0	50.0			
Mass	Calc. Mass	mDa	PPM	DBE	i-FIT	i-FIT (Norm)	Formula
780.4553	780.4548	0.5	0.6	14.5	59.8	0.0	C42 H62 N5 O9

S76. **Table S9**. NMR Data (500/125 MHz) of Microginin KR815 (**8**) in DMSO-d_6

Position	δ_C	δ_H Multiplicity, *J* (Hz)	HMBC correlations	COSY correlations	NOESY correlations
Ahda 1	170.1 C		Ahda-2, ^{1}Tyr-2,NH		
2	68.4 CH	4.30 brs		Ahda-2-OH,3	Ahda-2-OH,3,3-*N*CH_3, ^{1}Tyr-NH
2-OH		6.40 brm		Ahda-2	Ahda-2,3
3	60.3 CH	3.24 brm	Ahda-2,3-*N*CH_3	Ahda-2,4,4’	Ahda-2,2-OH
3-NH		8.42 brm		3-*N*CH_3	Ahda-3-*N*CH_3
3-*N*CH_3	30.8 CH_3	2.55 brs		Ahda-3-NH	Ahda-2,2-OH,3,3-NH
4	26.0 CH_2	1.39 m	Ahda-2,5,5’	Ahda-3,4’,5,5’	
		1.31 m		Ahda-3,4,5,5’	
5	25.1 CH_2	1.24 m	Ahda-4,4’,6	Ahda-4,4’,5’,6	
		1.13 m		Ahda-4,4’,5,6	
6	29.0 CH_2	1.13 m	Ahda-4,5,7,8	Ahda-7	
7	28.2 CH_2	1.22 m	Ahda-5,5’6,8	Ahda-8	
8	26.4 CH_2	1.33 tt, 7.6,7.1	Ahda-6’7,9,10	Ahda-7,9	
9	32.2 CH_2	1.67 tt, 7.6,6.6	Ahda-8,10	Ahda-8,10	
10	45.5 CH_2	3.60 t, 6.6	Ahda-9,8	Ahda-9	
^{1}Tyr 1	171.0 C		^{1}Tyr-2,3,3’, *N*MeLeu-2, *N*CH_3		
2	50.6 CH	4.86 ddd, 8.0,6.6,6.0	^{1}Tyr-3,3’	^{1}Tyr-2-NH,3,3’	^{1}Tyr-2-NH,3,3’,5,5’, *N*MeLeu-*N*Me
2-NH		8.09 d, 8.0		^{1}Tyr-2	^{1}Tyr-2,3,3’, Ahda-2
3	36.4 CH_2	2.85 m	^{1}Tyr-2,5,5’	^{1}Tyr-2,3’	^{1}Tyr-2,2-NH,3’,5,5’
		2.74 dd, 13.9,8.0		^{1}Tyr-2,3	^{1}Tyr-2,2-NH,3,5,5’
4	126.9 C		^{1}Tyr-2,3,3’,6,6’		
5,5’	130.3 CH	6.99 d, 8.4	^{1}Tyr-3,3’,5’,5	^{1}Tyr-6,6’	^{1}Tyr-2,3,3’,6,6’
6,6’	115.2 CH	6.62 d, 8.4	^{1}Tyr-5,5’,6’,6,7-OH	^{1}Tyr-5,5’	^{1}Tyr-5,5’,7-OH
7	156.2 C		^{1}Tyr-5,5’,6,6’,7-OH		
7-OH		9.25 s			^{1}Tyr-6,6’
*N*MeLeu 1	168.4 C		*N*MeLeu-2,3,3’		

2	51.9 CH	5.23 dd, 8.0,6.5	*N*MeLeu-3,3’,*N*Me	*N*MeLeu-3,3’	*N*MeLeu-3,3’,5,6,*N*Me, Pro-5,5’
2-*N*CH_3	30.2 CH_3	2.89 s	*N*MeLeu-2		*N*MeLeu-2, [1]Tyr-2
3	37.1 CH_2	1.47 m	*N*MeLeu-2,4,5,6	*N*MeLeu-2,3’,4	*N*MeLeu-2,5,6
		1.41 m		*N*MeLeu-2,3,4	*N*MeLeu-2,5,6
4	24.2 CH	1.38 m	*N*MeLeu-2,3,3’,5,6	*N*MeLeu-3,3’,5,6	*N*MeLeu-2
5	22.4 CH_3	0.81 d, 6.5	*N*MeLeu-3,3’,4,6	*N*MeLeu-4	*N*MeLeu-2,3,3’
6	23.0 CH_3	0.85 d, 6.5	*N*MeLeu-3,3’,4,5	*N*MeLeu-4	*N*MeLeu-2,3,3’
Pro 1	171.7 C		Pro-2,3, [2]Tyr-NH		
2	59.3 CH	4.29 m		Pro-3,3’	Pro-3,3’,5’
3	29.1 CH_2	1.97 m	Pro-2,4,4’,5,5’	Pro-2,3’,4,4’	Pro-2
		1.74 m		Pro-2,3,4,4’,5’	Pro-2
4	24.3 CH_2	1.78 m	Pro-2,3,3’5,5’	Pro-3,3’,5,5’	
		1.73 m		Pro-3,3’,5,5’	
5	46.7 CH_2	3.40 m	Pro-2	Pro-4,4’,5’	Pro-5’, *N*MeLeu-2
		3.18 m		Pro-3,4,4’,5	Pro-2,5, *N*MeLeu-2
[2]Tyr 1	172.2 C		[2]Tyr-2,3,3’,OCH_3		
2	54.2 CH	4.30 m	[2]Tyr-2-NH,3,3’	[2]Tyr-2-NH,3,3’	[2]Tyr-3,3’,5,5’,NH
2-NH		8.15 d, 7.6		[2]Tyr-2	[2]Tyr-2,3,3’
3	36.1 CH_2	2.86 m	[2]Tyr-2,2-NH,5,5’	[2]Tyr-2,3’	[2]Tyr-2,3’,5,5’,NH
		2.82 m		[2]Tyr-2,3	[2]Tyr-2,3,5,5’,NH
4	127.2 C		[2]Tyr-2,3,3’,6,6’		
5,5’	130.2 CH	7.00 d, 8.4	[2]Tyr-3,3’,5’,5	[2]Tyr-6,6’	[2]Tyr-2,3,3’,6,6’
6,6’	115.1 CH	6.64 d, 8.4	[2]Tyr-5,5’,6’,6,7-OH	[2]Tyr-5,5’	[2]Tyr-5,5’,7-OH
7	156.2 C		[2]Tyr-5,5’,6,6’,7-OH		
7-OH		9.23 s			[2]Tyr-6,6’
OCH_3	51.8 CH_3	3.53 s			

S78. ^{1}H NMR Spectrum of Microginin KR815 (**8**) in DMSO-d_6

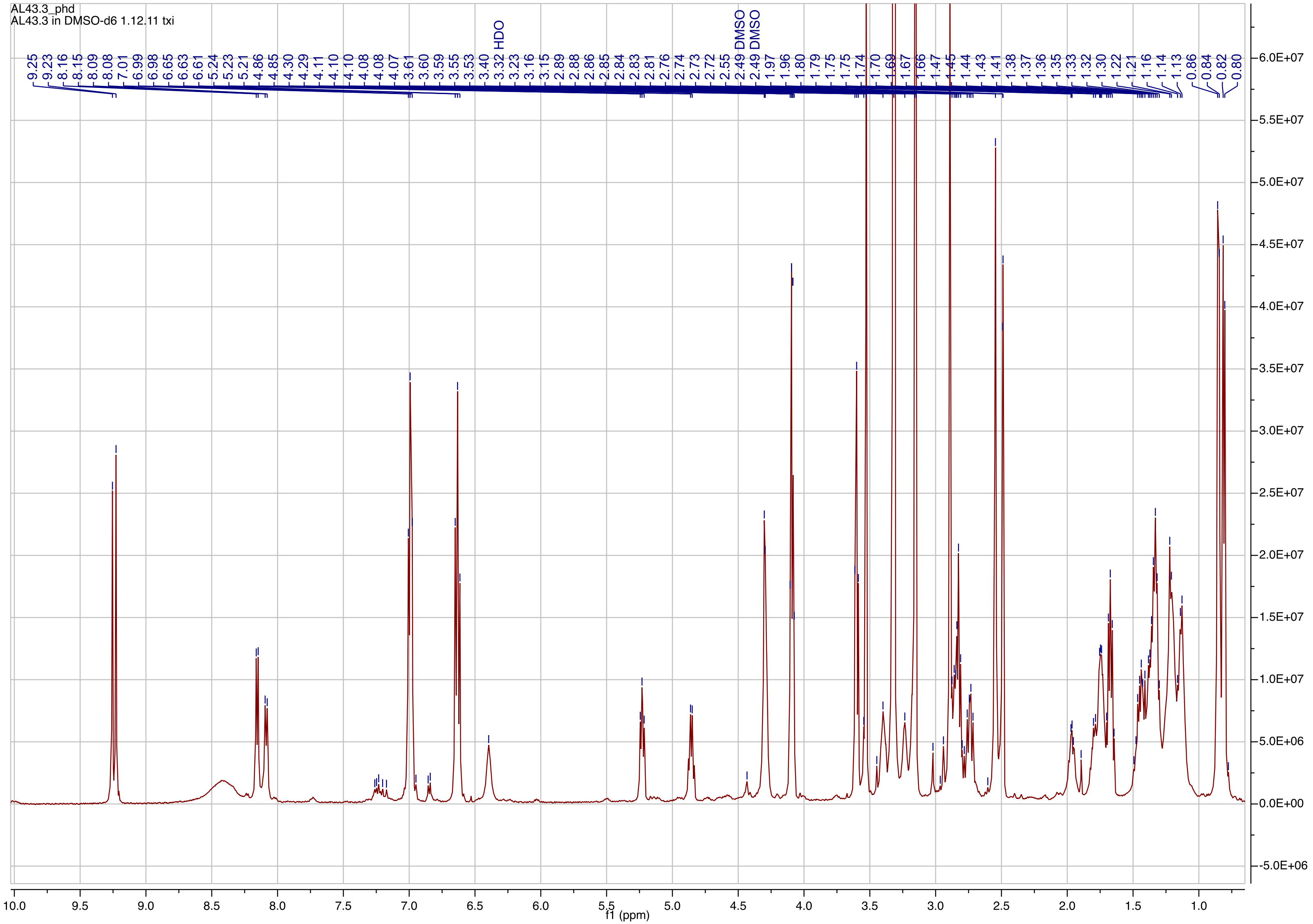

S79. ^{13}C NMR Spectrum of Microginin KR815 (**8**) in DMSO-d_6

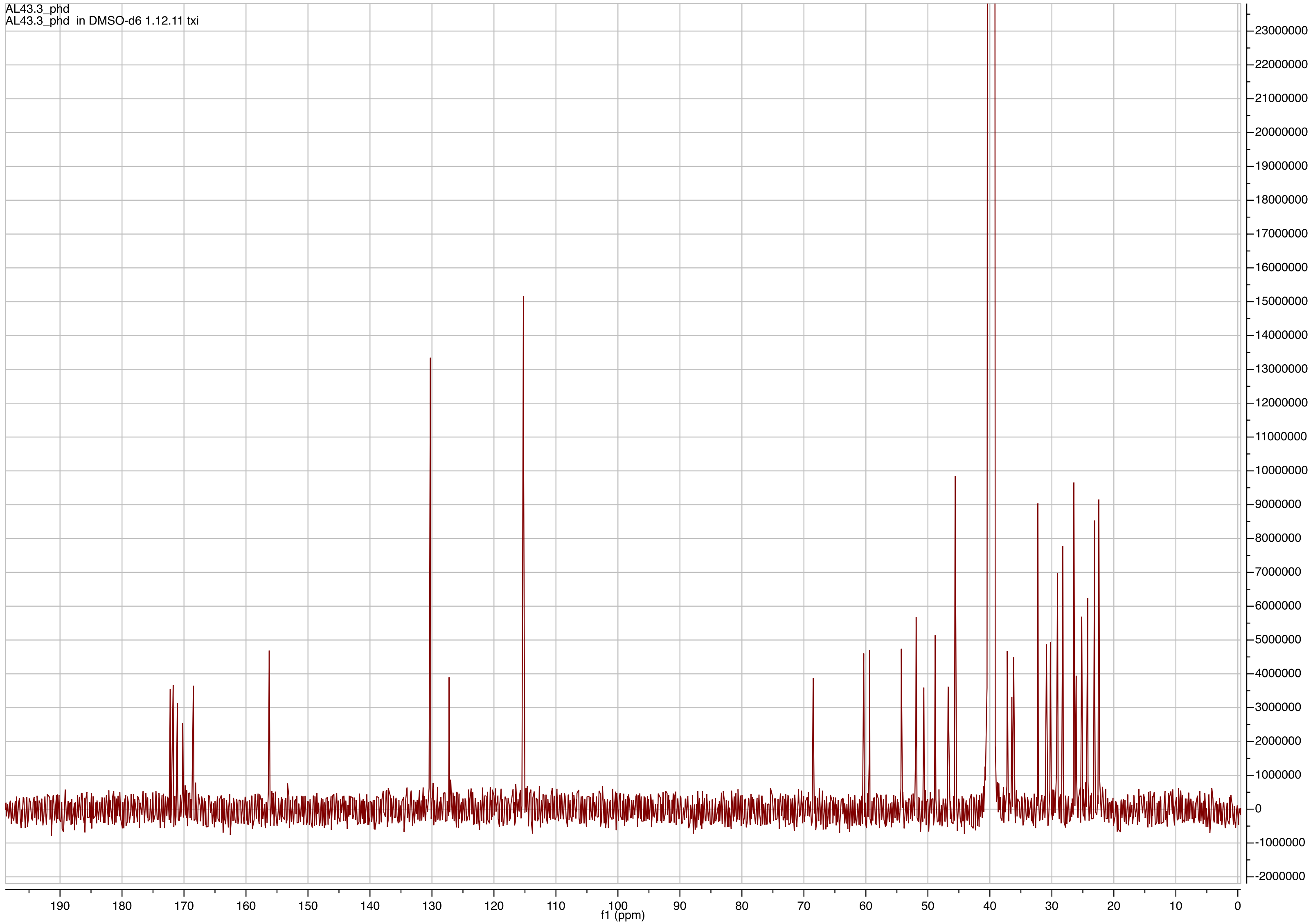

S80. HSQC Spectrum Microginin KR815 (**8**) in DMSO-d_6

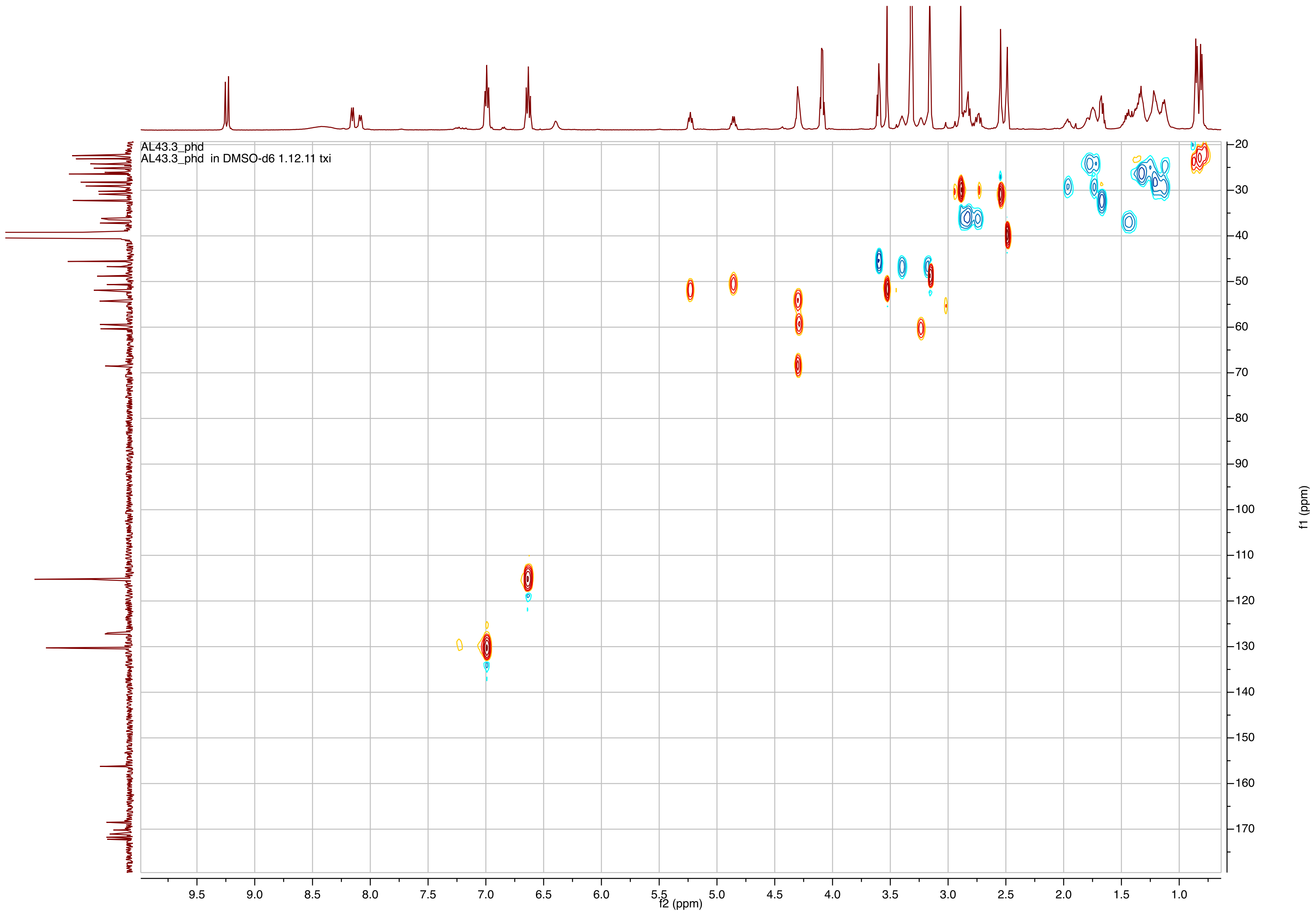

S81. HMBC Spectrum of Microginin KR815 (**8**) in DMSO-d_6

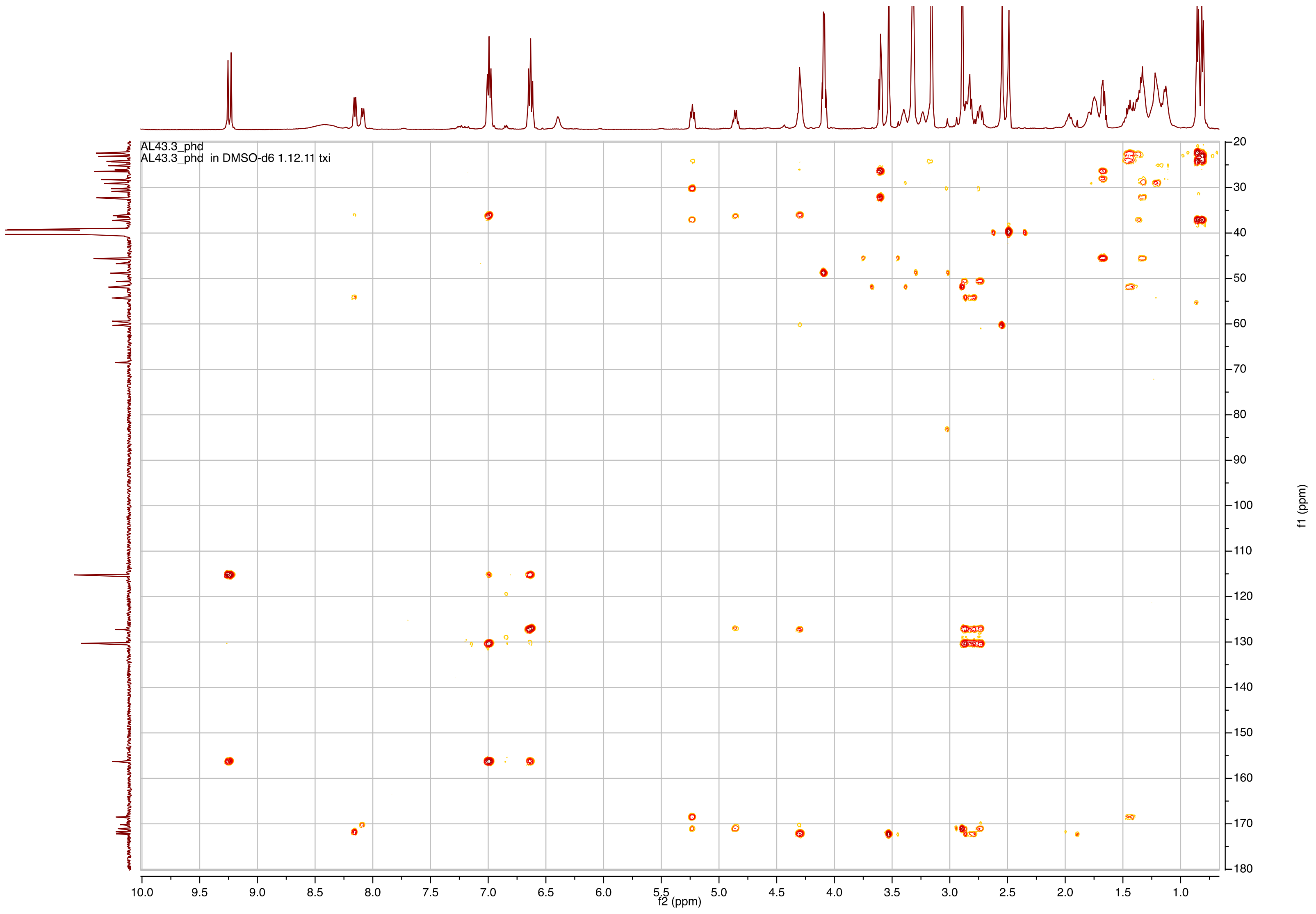

S82. COSY Spectrum of Microginin KR815 (**8**) in DMSO-d_6

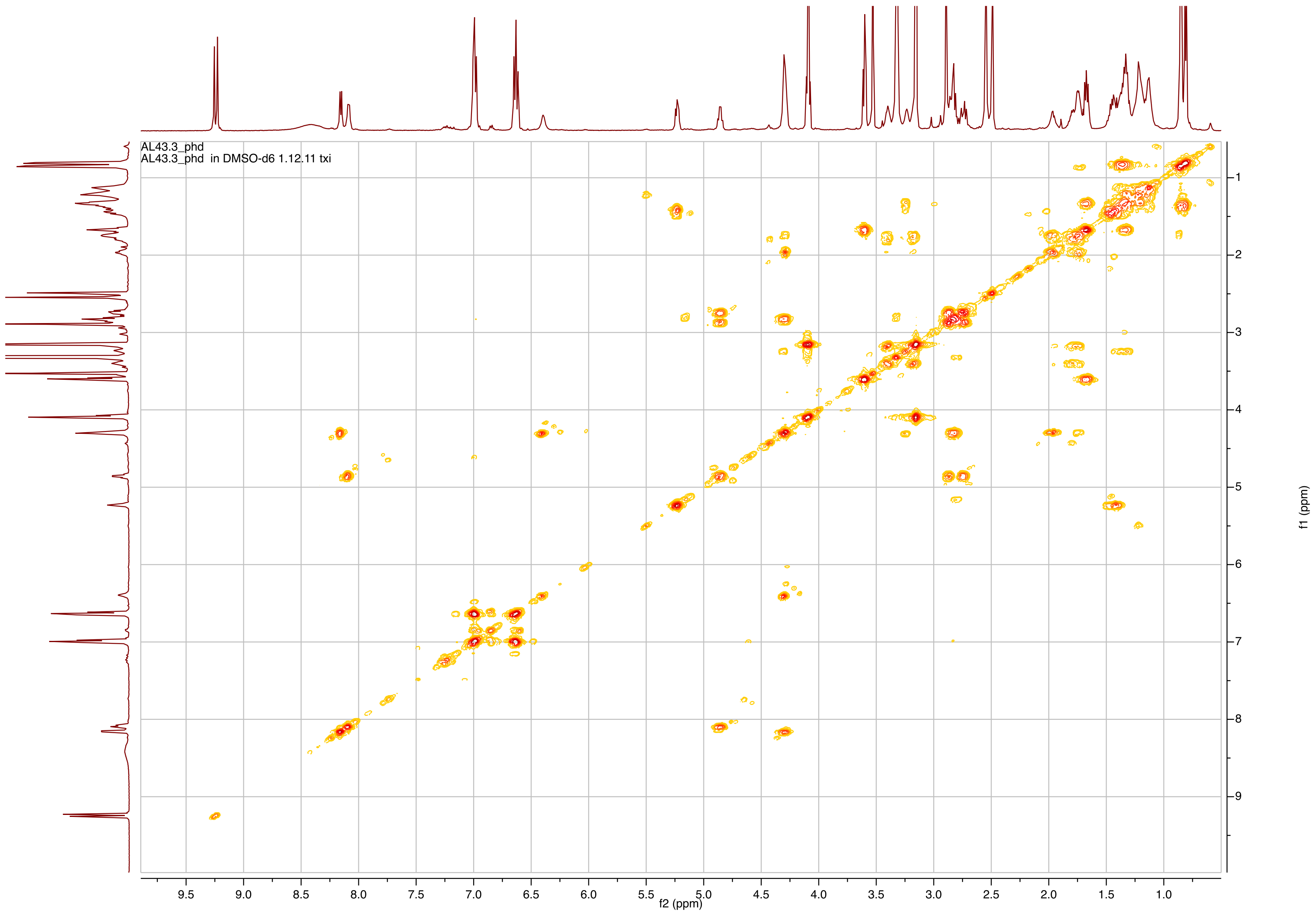

S83. TOCSY Spectrum of Microginin KR815 (**8**) in DMSO-d_6

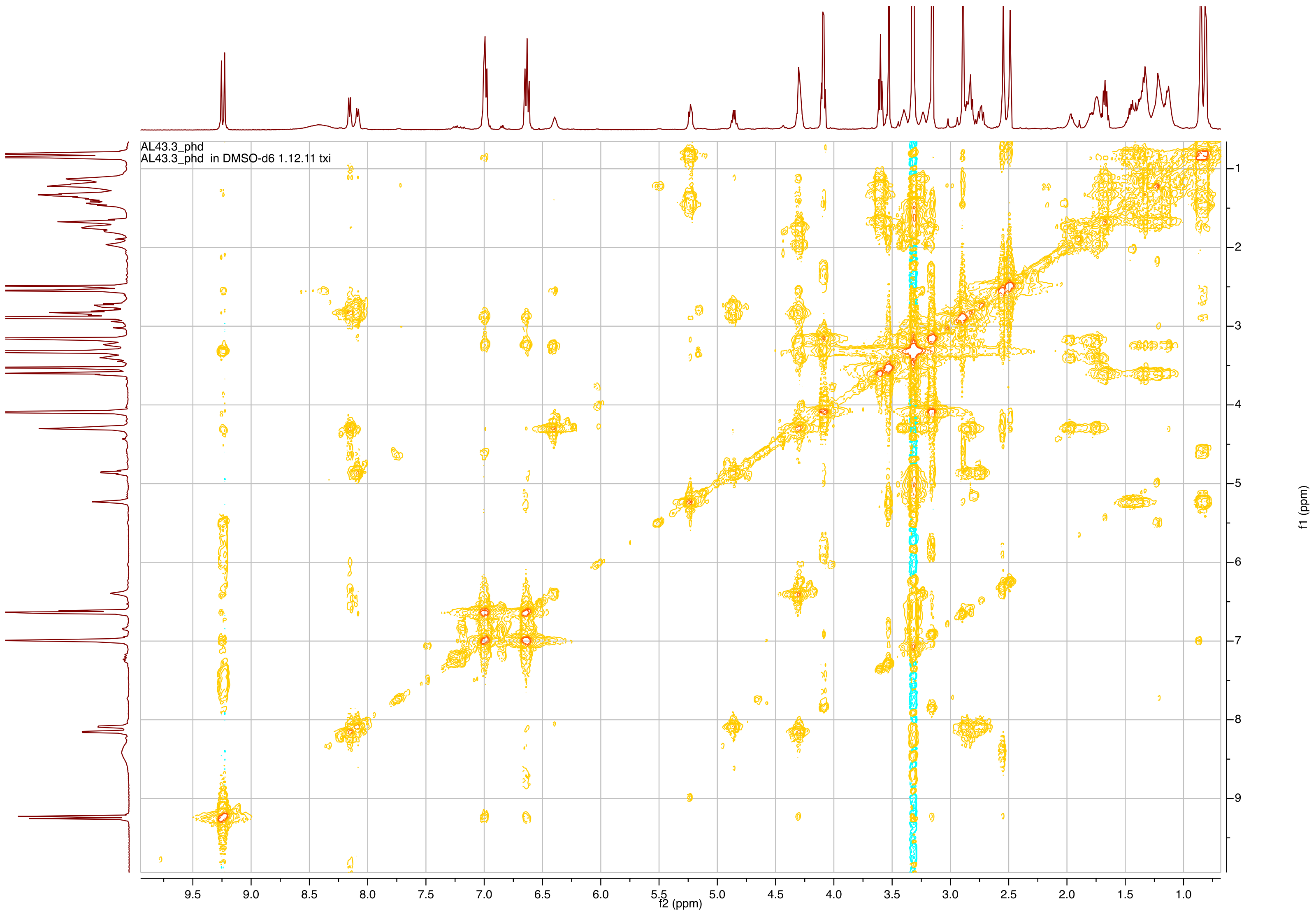

S84. ROESY Spectrum of Microginin KR815 (**8**) in DMSO-d_6

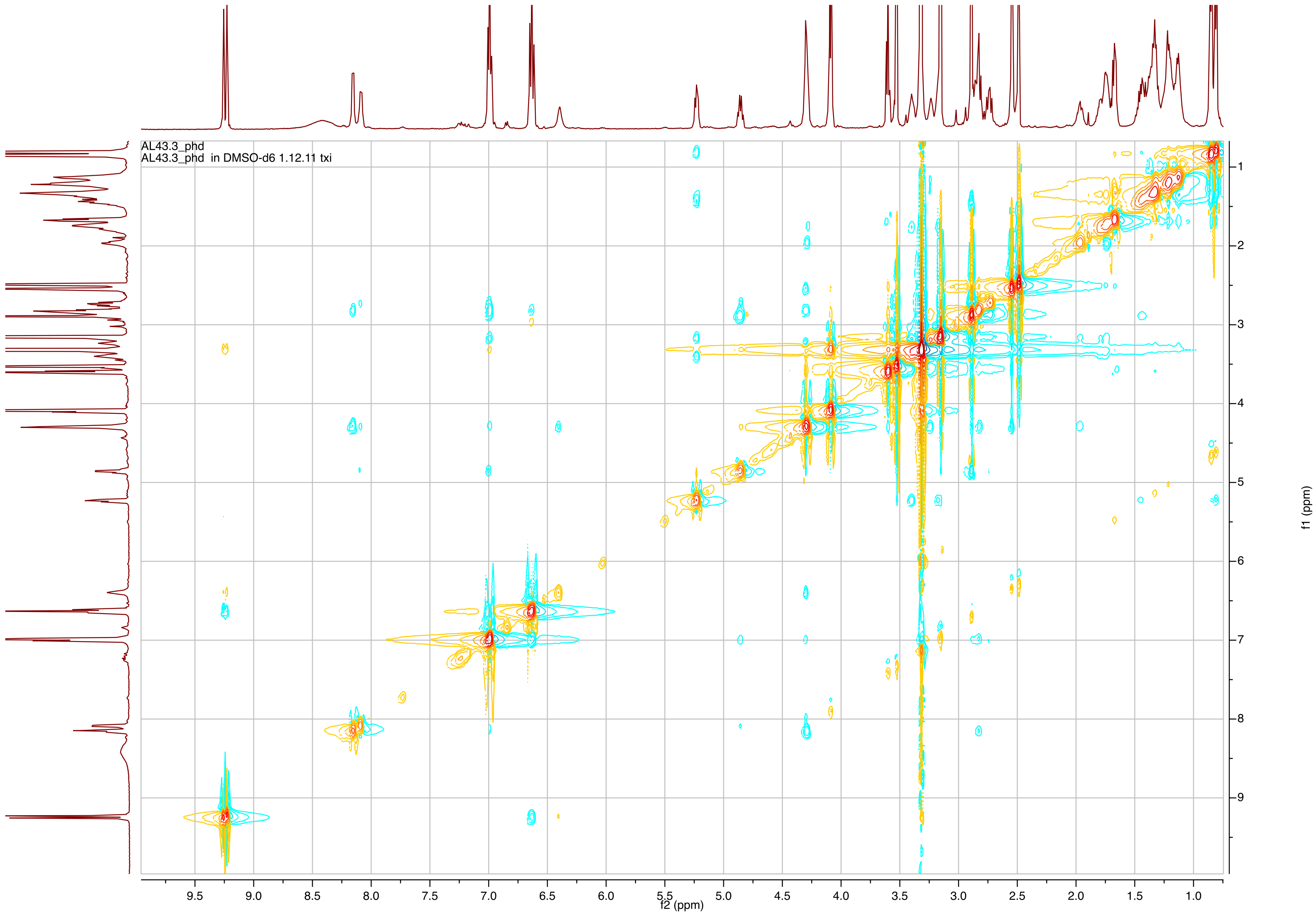

S85. HR ESI MS data of Microginin KR815 (**8**)

Elemental Composition Report Page 1

Single Mass Analysis
Tolerance = 1.0 PPM / DBE: min = -1.5, max = 50.0
Element prediction: Off
Number of isotope peaks used for i-FIT = 3

Monoisotopic Mass, Even Electron Ions
447 formula(e) evaluated with 2 results within limits (all results (up to 1000) for each mass)
Elements Used:
C: 38-50 H: 55-70 N: 0-10 O: 0-15 Cl: 0-2
AL43.3 Anat Lodin
carmeli407 132 (5.803) Cm (132) 1: TOF MS ES+ 6.77e+003

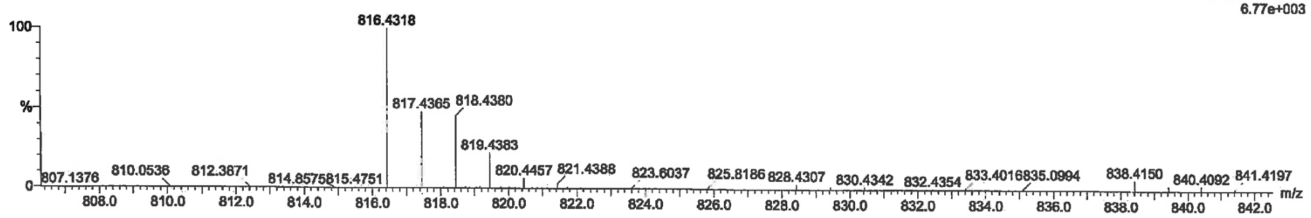

Minimum:				-1.5			
Maximum:		5.0	1.0	50.0			
Mass	Calc. Mass	mDa	PPM	DBE	i-FIT	i-FIT (Norm)	Formula
816.4318	816.4314	0.4	0.5	13.5	76.4	0.0	C42 H63 N5 O9 Cl
	816.4323	-0.5	-0.6	17.5	82.9	6.5	C47 H62 N O11

S86. **Table S10**. NMR Data (500/125 MHz) of Microginin FR3 (**9**) in DMSO-d_6

Position	δ_C	δ_H Multiplicity, J (Hz)	HMBC correlations	COSY correlations	NOESY correlations
Ahda 1	170.8 C		Ahda-2,2-OH, Thr-2,NH		
2	69.6 CH	4.10 t, 4.6	Ahda-2-OH	Ahda-2-OH,3	Ahda-2-OH,3,3-NH_3,4,4',5',5', Thr-2-NH
2-OH		6.61 m		Ahda-2	Ahda-2,3,4
3	53.1 CH	3.22 m	Ahda-2-OH	Ahda-3-NH_3,4,4'	Ahda-2,2-OH,3-NH_3,4,4'5,5', Thr-2-NH
3-NH_3		7.71 m		Ahda-3	Ahda-2,3, ,4,4'5,5'
4	28.8 CH_2	1.59 m	Ahda-2,5,5',6	Ahda-3,4',5,5'	Ahda-2,2-OH,3,3-NH_3,4',5',5'
		1.44 m		Ahda-3,4,5,5'	Ahda-2,3,3-NH_3,4,5,5'
5	24.8 CH_2	1.34 m	Ahda-6,7	Ahda-4,4',5',6	Ahda-2,3,3-NH_3,4,4',5'
		1.29 m		Ahda-4,4',5,6	Ahda-2,3,3-NH_3,4,4',5
6	28.5 CH_2	1.23 m	Ahda-5,5',7,8	Ahda-5,5'	
7	28.9 CH_2	1.23 m			
8	31.3 CH_2	1.23 m	Ahda-7,9,10		
9	22.2 CH_2	1.24 m	Ahda-8,10	Ahda-10	Ahda-10
10	14.1 CH_3	0.85 t, 6.8	Ahda-8,9	Ahda-9	Ahda-9
Thr 1	168.6 C		Thr-2		
2	56.0 CH	4.48 dd, 7.2,6.6	Thr-2-OH,4	Thr-2-NH,3	Thr-2-NH,3,4, Pro-5
2-NH		7.86 d, 7.2		Thr-2	Thr-2,3,4, Ahda-2,3
3	67.0 CH	3.94 brdq, 6.6,6.6	Thr-2,3-OH,4	Thr-2,3-OH,4	Thr-2,2-NH,3-OH,4
3-OH		5.13 d, 3.6		Thr-3	Thr-3,4
4	19.3 CH_3	1.14 d. 6.6	Thr-2	Thr-3	Thr-2,2-NH,3,3-OH, ^{2}Tyr-6,6'
Pro 1	171.0 C		Pro-2,3, ^{1}Tyr-NH		
2	59.5 CH	4.32 dd, 7.8,3.2		Pro-3,3'	Pro-3,3'
3	29.0 CH_2	1.90 m	Pro-4,4',5	Pro-2,3',4,4'	Pro-2,3',5
		1.69 m		Pro-2,3,4,4'	Pro-2,3, ^{1}Tyr-2-NH,5,5'
4	24.2 CH_2	1.74 m	Pro-3,5	Pro-3,3',4',5	Pro-5
		1.62 m		Pro-3,3',4,5	Pro-5, ^{1}Tyr-5,5'

5	47.4 CH_2	3.63 m		Pro-4,4'	Pro-3,4,4', ^{1}Tyr-5,5', Thr-2
^{1}Tyr 1	171.0 C		^{1}Tyr-2,3,3', ^{2}Tyr-NH		
2	54.1 CH	4.38 ddd, 9.5,8.2,4.8	^{1}Tyr-2-NH,3,3'	^{1}Tyr-2-NH,3,3'	^{1}Tyr-2-NH,3,3'5,5' ^{2}Tyr-2-NH
2-NH		7.64 d, 8.2		^{1}Tyr-2	^{1}Tyr-2,3,3', ^{2}Tyr-2, Pro-3',5
3	36.6 CH_2	2.87 dd, 13.8,4.8	^{1}Tyr-2,2-NH,5,5'	^{1}Tyr-2,3'	^{1}Tyr-2,2-NH,5,5'
		2.63 dd, 13.8,9.5		^{1}Tyr-2,3	^{1}Tyr-2,2-NH,5,5'
4	127.8 C		^{1}Tyr-3,3',5,5'		
5,5'	130.2 CH	7.00 d, 8.5	^{1}Tyr-3,3',5',5	^{1}Tyr-6,6'	^{1}Tyr-2,3,3',6,6', Pro-3',4',5, Thr-3,4
6,6'	114.9 CH	6.61 d, 8.5	^{1}Tyr-5,5',6',6,7-OH	^{1}Tyr-5,5'	^{1}Tyr-5,5',7-OH
7	155.9 C		^{1}Tyr-5,5',6,6',7-OH		
7-OH		9.13 s			^{1}Tyr-6,6'
^{2}Tyr 1	172.9 C		^{2}Tyr-2,3,3'		
2	53.9 CH	4.31 m	^{2}Tyr-2-NH,3,3'	^{2}Tyr-2-NH,3,3'	^{2}Tyr-2-NH,3,3'
2-NH		8.03 d, 7.6		^{2}Tyr-2	^{2}Tyr-2,3,3', ^{1}Tyr-2
3	36.2 CH_2	2.89 dd, 14.1,5.9	^{2}Tyr-2,5,5'	^{2}Tyr-2,3'	^{2}Tyr-2,2-NH,
		2.79 dd, 14.1,7.8		^{2}Tyr-2,3	^{2}Tyr-2,2-NH,
4	127.4 C		^{2}Tyr-2,3,3',5,5'		
5,5'	130.2 CH	7.00 d, 8.1	^{2}Tyr-3,3',5',5	^{2}Tyr-6,6'	^{2}Tyr-2,3,3',6,6'
6,6'	115.1 CH	6.63 d, 8.1	^{2}Tyr-5,5',6',6,7-OH	^{2}Tyr-5,5'	^{2}Tyr-5,5',7-OH, Thr-4
7	156.1 C		^{2}Tyr-5,5',6,6'7-OH		
7-OH		9.18 s			^{2}Tyr-6,6'

S88. ^{1}H NMR Spectrum of Microginin FR3 (**9**) in DMSO-d_6

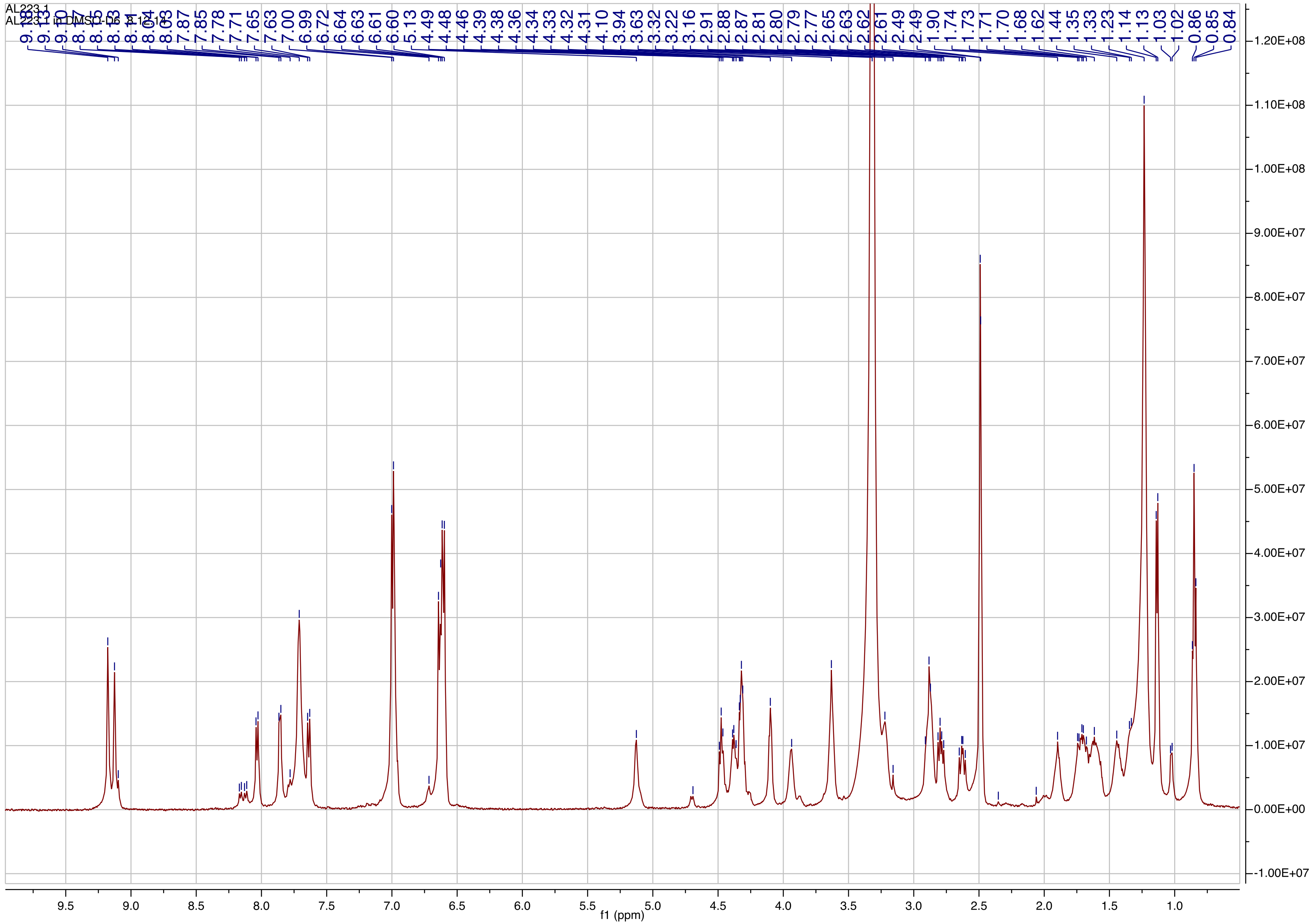

S89. ^{13}C NMR Spectrum of Microginin FR3 (**9**) in DMSO-d_6

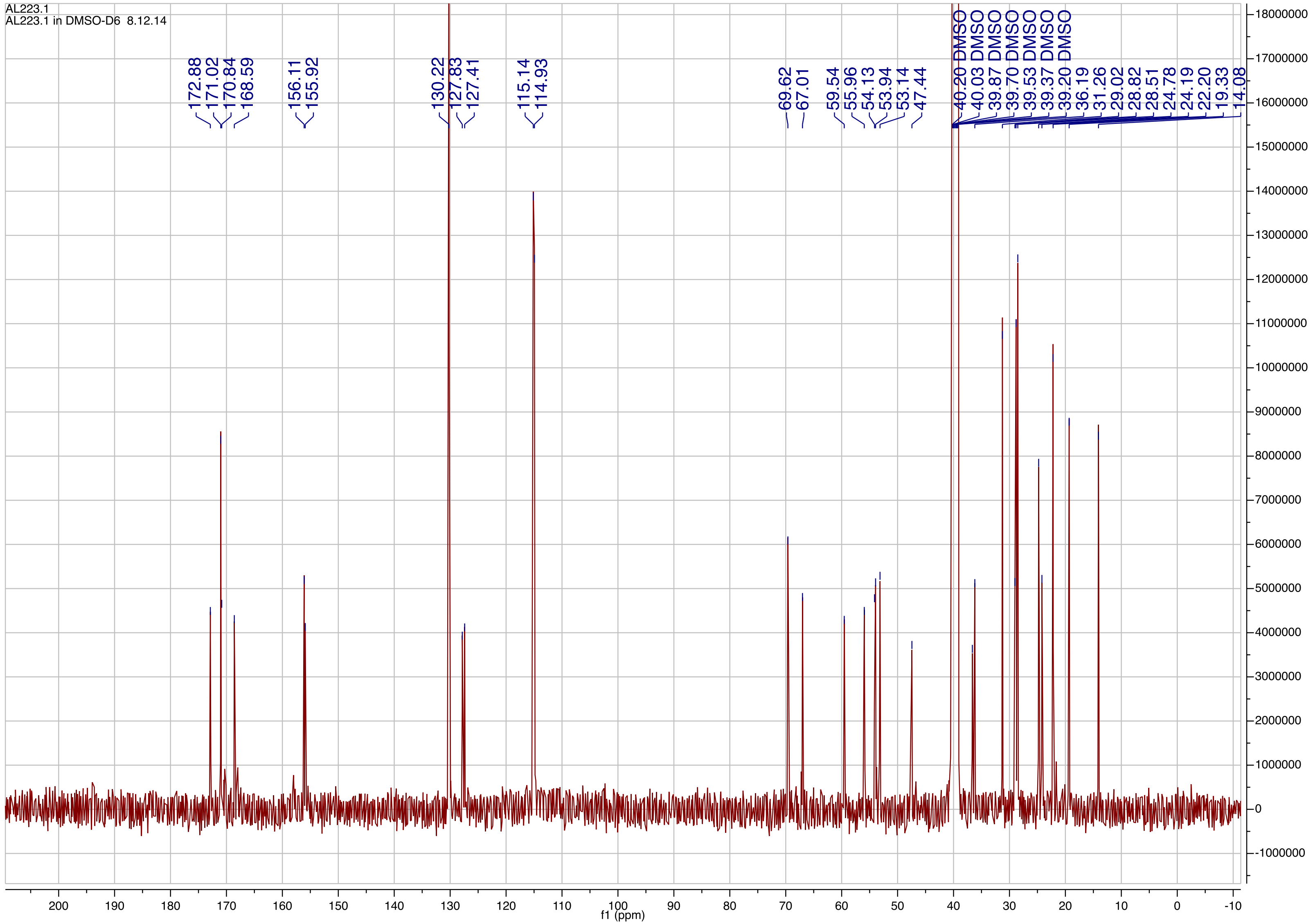

S90. HSQC Spectrum Microginin FR3 (**9**) in DMSO-d_6

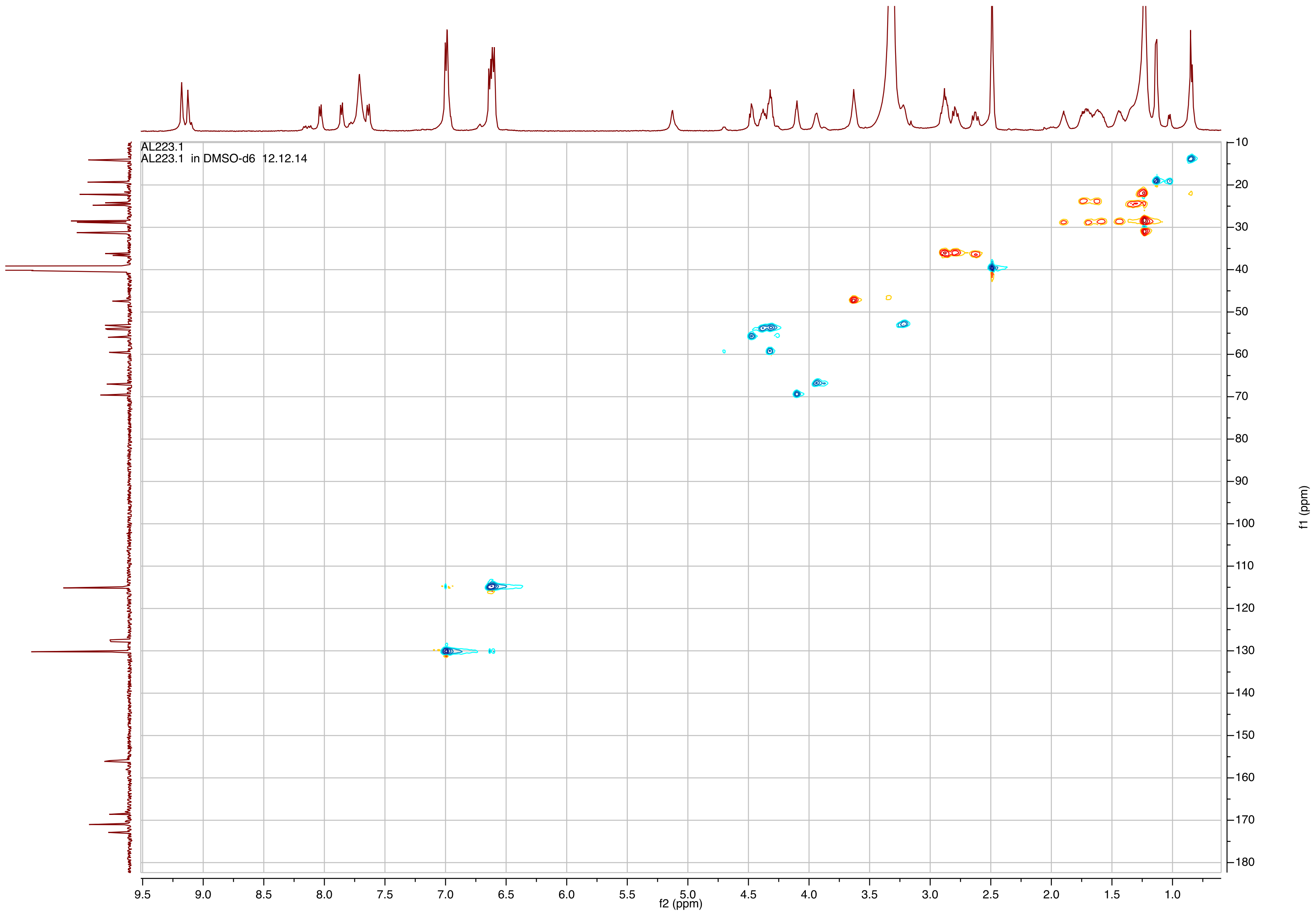

S91. HMBC Spectrum of Microginin FR3 (**9**) in DMSO-d_6

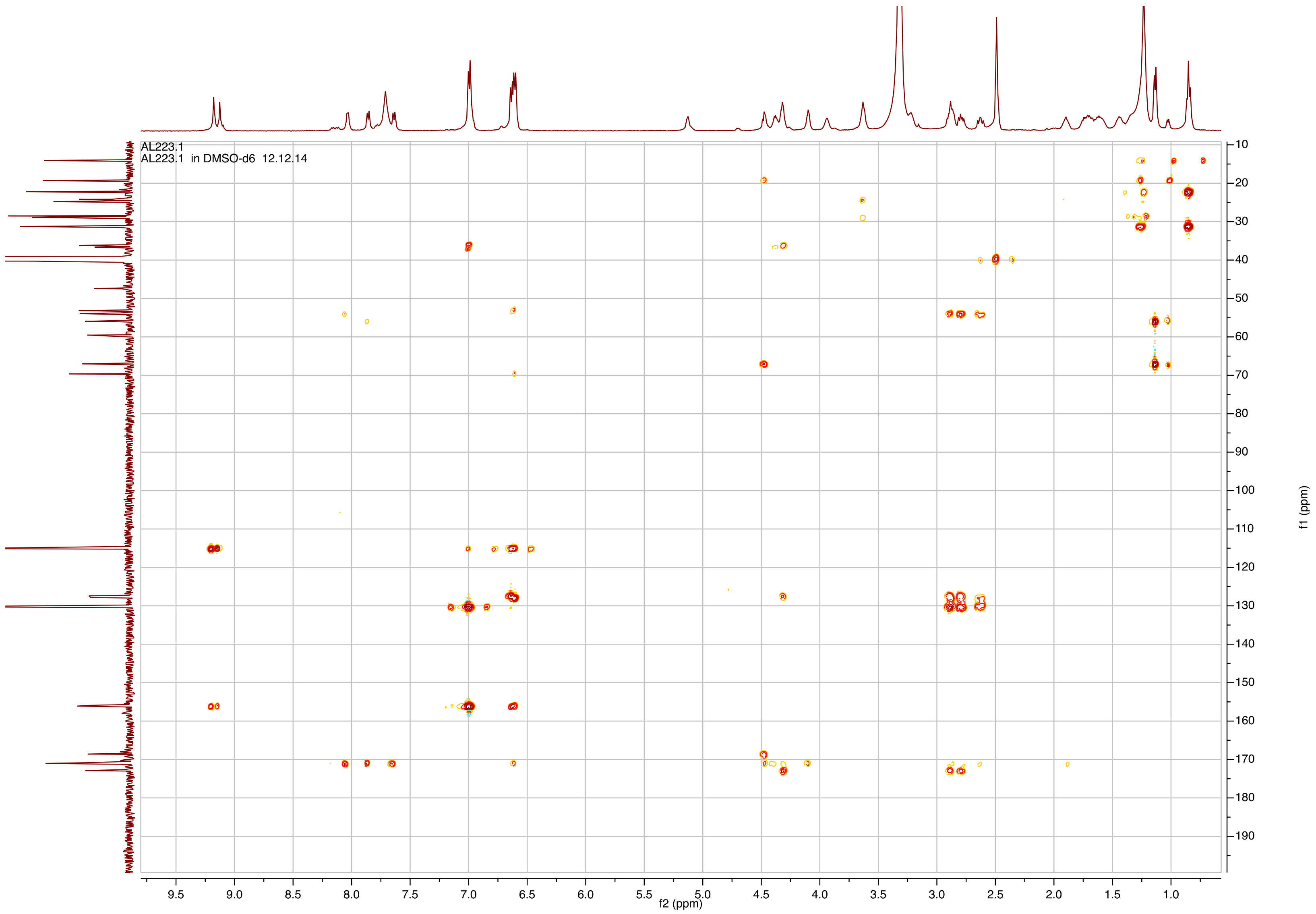

S92. COSY Spectrum of Microginin FR3 (**9**) in DMSO-d_6

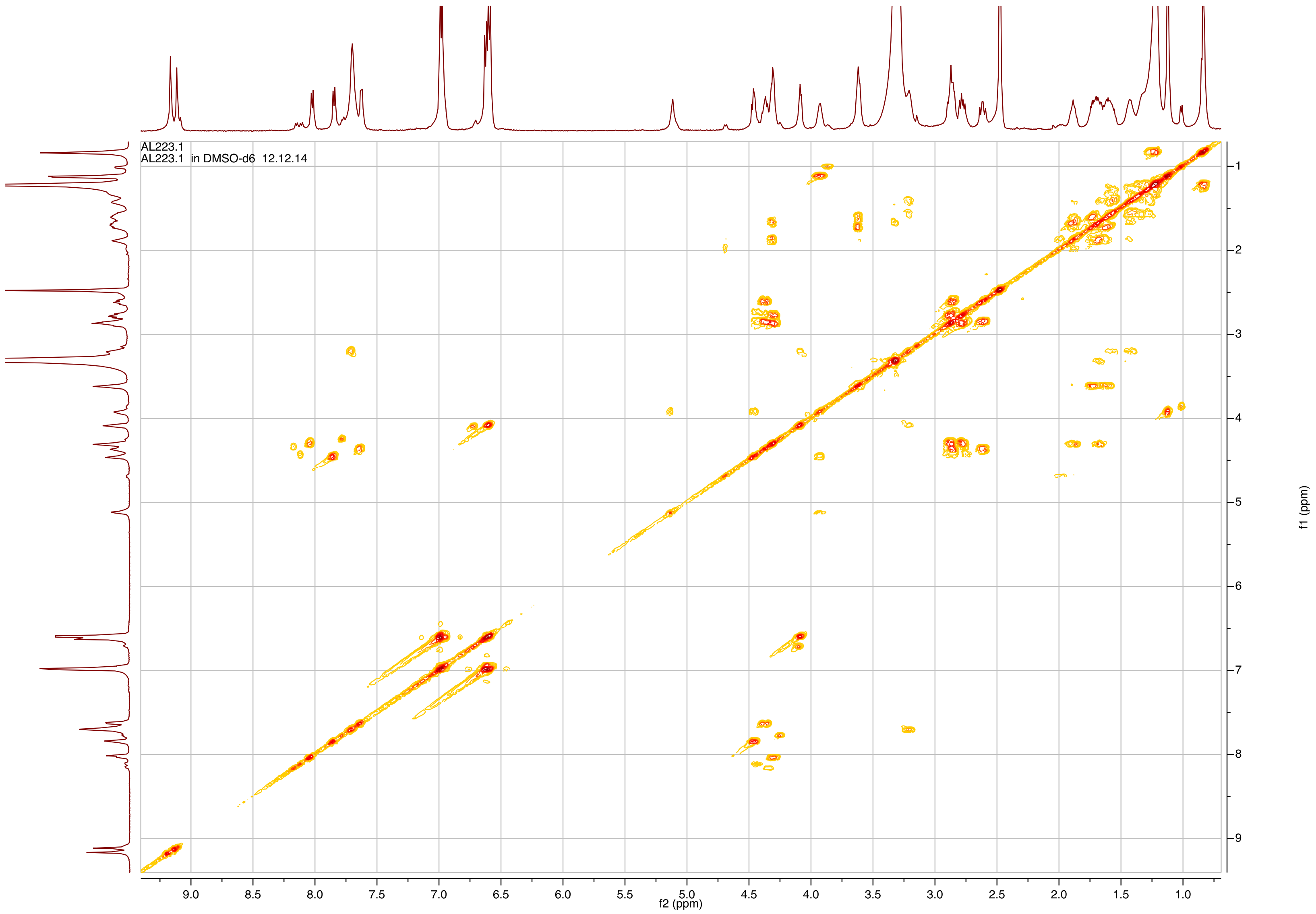

S93. TOCSY Spectrum of Microginin FR3 (**9**) in DMSO-d_6

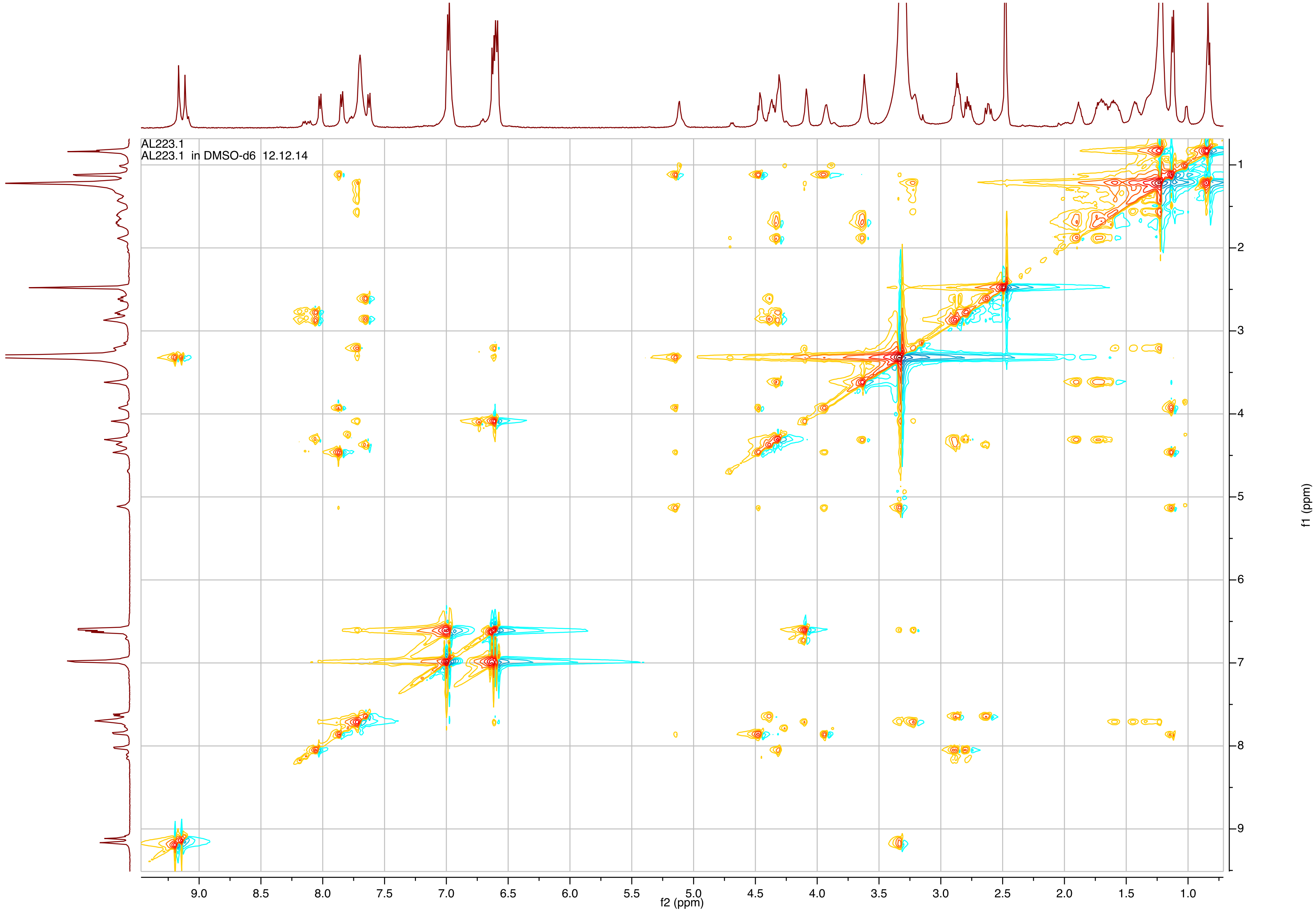

S94. ROESY Spectrum of Microginin FR3 (**9**) in DMSO-d_6

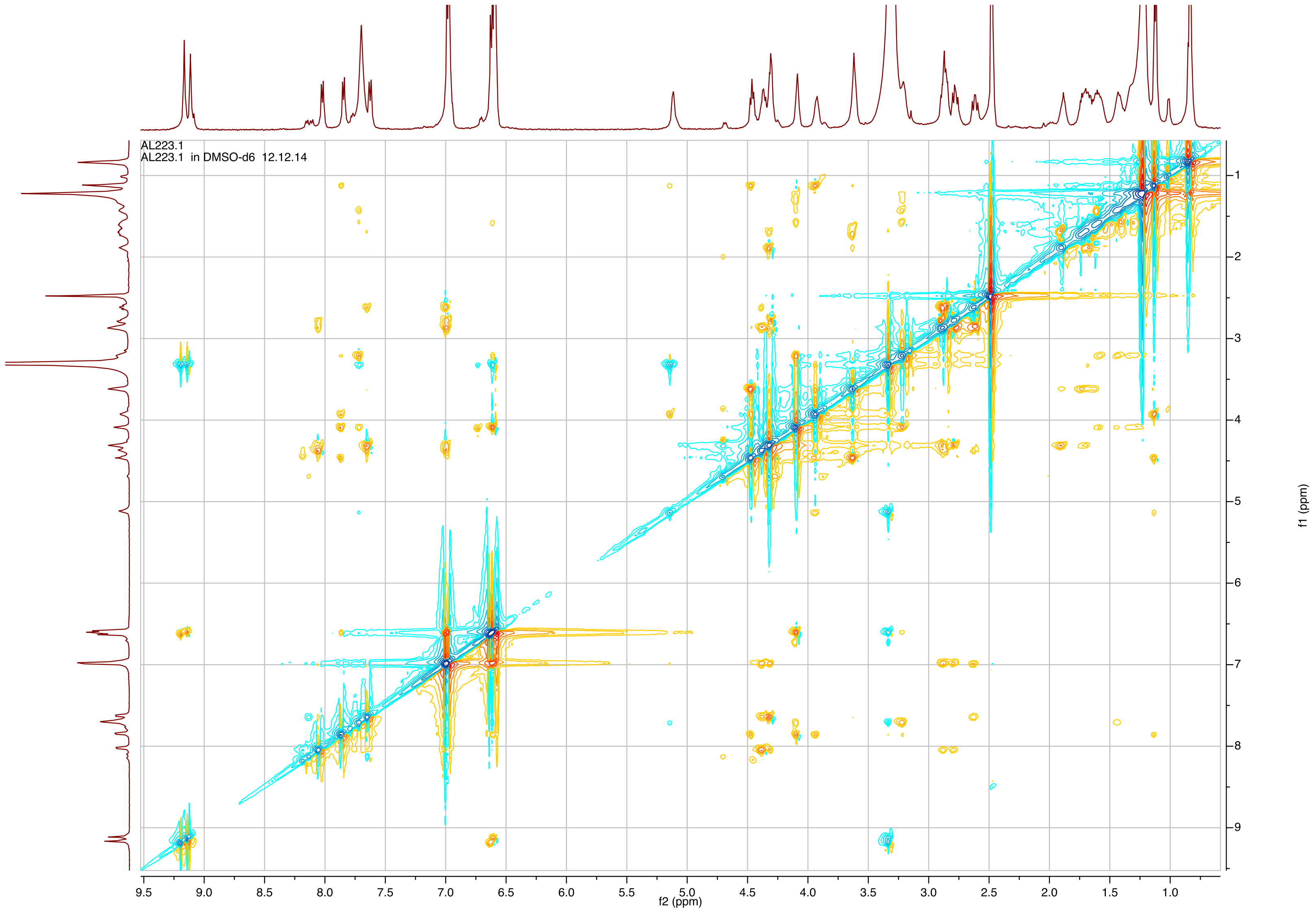

S95. HR ESI MS data of Microginin FR3 (**9**)

Elemental Composition Report

Page 1

Single Mass Analysis
Tolerance = 2.0 PPM / DBE: min = -1.5, max = 50.0
Element prediction: Off
Number of isotope peaks used for i-FIT = 3

Monoisotopic Mass, Even Electron Ions
140 formula(e) evaluated with 2 results within limits (all results (up to 1000) for each mass)
Elements Used:
C: 35-45 H: 50-65 N: 0-10 O: 0-15
AL223.1
carmeli968 46 (2.033) Cm (46:47)

Anat Lodin

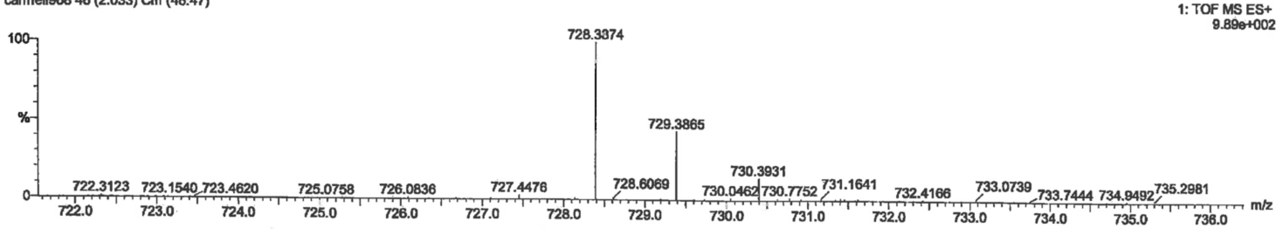

Mass	Calc. Mass	mDa	PPM	DBE	i-FIT	i-FIT (Norm)	Formula
Minimum:				-1.5			
Maximum:		5.0	2.0	50.0			
728.3874	728.3871	0.3	0.4	13.5	63.0	0.3	C37 H54 N5 O10 ←
	728.3884	-1.0	-1.4	18.5	64.0	1.3	C38 H50 N9 O6

S96. **Table S11**. NMR Data (500/125 MHz) of Microginin FR4 (**10**) in DMSO-d_6

Position	δ_C	δ_H Multiplicity, *J* (Hz)	HMBC correlations	COSY correlations	NOESY correlations
Ahda 1	171.2 C		Ahda-2-OH, Thr-2,NH		
2	68.2 CH	4.18 t, 4.4	Ahda-4'	Ahda-2-OH,3	Ahda-2-OH,3,3-NH_2,4,4',5',5', Thr-2-NH
2-OH		6.82 d, 4.4		Ahda-2	Ahda-2,3,4,5, Thr-2-NH
3	60.1 CH	3.30 m	Ahda-3-NCH_3,4,4'	Ahda-2,3-NH,4,4'	Ahda-2,2-OH,3-NH_2,4, Thr-2-NH
3-NH_2		8.36 brs		Ahda-3,NH',NCH_3	Ahda-2,3,NH',4,4'5,5', Thr-2
		8.17 brs		Ahda-3,NH,NCH_3	Ahda-3-NH,
3-NCH_3	31.3 CH_3	2.50 brt, 5.3			Thr-2-NH
4	27.9 CH_2	1.58 m	Ahda-5,5'	Ahda-3,4',5,5'	Ahda-2,2-OH,3,3-NH_2,
		1.55 m		Ahda-3,4,5,5'	Ahda-2,3,3-NH_2,
5	25.1 CH_2	1.33 m	Ahda-4,4'	Ahda-4,4',5',6	Ahda-2,3,3-NH_2,
		1.30 m		Ahda-4,4',5,6	Ahda-2
6	28.6 CH_2	1.23 m	Ahda-7,8	Ahda-5,5'	
7	28.9 CH_2	1.23 m	Ahda-5,5',9		
8	31.4 CH_2	1.23 m	Ahda-9,10		
9	22.2 CH_2	1.24 m	Ahda-8,10	Ahda-10	Ahda-10
10	14.1 CH_3	0.85 t, 6.8	Ahda-8,9	Ahda-9	Ahda-9
Thr 1	168.5 C		Thr-2		
2	56.2 CH	4.47 dd, 7.4,6.0	Thr-4	Thr-2-NH,3	Thr-2-NH,3,4, Pro-5, Ahda-3-NH, ^{1}Tyr-5,5'
2-NH		7.91 d, 7.4		Thr-2	Thr-2,3,4, Ahda-2,2-OH,3,3-NCH_3
3	67.0 CH	3.93 brdq, 6.0,6.0	Thr-2,4	Thr-2,4	Thr-2,2-NH,4, ^{1}Tyr-2-NH,5,5'
3-OH		5.10 brs			
4	19.5 CH_3	1.14 d, 6.0	Thr-2	Thr-3	Thr-2,2-NH,3, ^{1}Tyr-2-NH,5,5'
Pro 1	171.0 C		Pro-2, ^{1}Tyr-NH		
2	59.5 CH	4.32 m		Pro-3,3'	Pro-3,3'
3	29.1 CH_2	1.90 m	Pro-4,4',5	Pro-2,3',4,4',5	Pro-2,3'4,4'
		1.70 m		Pro-2,3,4,4'	Pro-2,3, ^{1}Tyr-2-NH
4	24.2 CH_2	1.74 m	Pro-3,5	Pro-3,3',4',5	Pro-3,5

		1.65 m		Pro-3,3’,4,5	Pro-3,5
5	47.5 CH_2	3.64 m		Pro-3,4,4’	Pro-4,4’, Thr-2
^{1}Tyr 1	171.0 C		^{1}Tyr-2,3, ^{2}Tyr-NH		
2	54.2 CH	4.38 ddd, 9.0,8.2,4.2	^{1}Tyr-3’	^{1}Tyr-2-NH,3,3’	^{1}Tyr-2-NH,3,3’,5,5’ ^{2}Tyr-2-NH
2-NH		7.64 d, 8.2		^{1}Tyr-2	^{1}Tyr-2,3’,6,6’, ^{2}Tyr-2, Pro-3’,5, Thr-3,4
3	36.7 CH_2	2.87 dd, 13.8,4.2	^{1}Tyr-5,5’	^{1}Tyr-2,3’	^{1}Tyr-2,3’,5,5’
		2.63 dd, 13.8,9.0		^{1}Tyr-2,3	^{1}Tyr-2,2-NH,3,5,5’
4	127.9 C		^{1}Tyr-2,3,3’,5,5’		
5,5’	130.2 CH	7.00 d, 8.6	^{1}Tyr-3,3’,5’,5	^{1}Tyr-6,6’	^{1}Tyr-2,3,3’,6,6’, Pro-3’,4’,5, Thr-2,3,4
6,6’	115.0 CH	6.60 d, 8.6	^{1}Tyr-5,5’,6’,6	^{1}Tyr-5,5’	^{1}Tyr-2-NH,5,5’,7-OH
7	155.9 C		^{1}Tyr-5,5’,6,6’		
7-OH		9.12 s			^{1}Tyr-6,6’
^{2}Tyr 1	172.9 C		^{2}Tyr-2,3,3’		
2	54.0 CH	4.31 m	^{2}Tyr-3,3’	^{2}Tyr-2-NH,3,3’	^{2}Tyr-2-NH,3,3’,5,5’
2-NH		8.04 d, 7.6		^{2}Tyr-2	^{2}Tyr-2,3,3’,6,6’,^{1}Tyr-2
3	36.2 CH_2	2.90 dd, 14.0,5.7	^{2}Tyr-2,5,5’	^{2}Tyr-2,3’	^{2}Tyr-2,2-NH,3’,5,5’
		2.79 dd, 14.0,7.9		^{2}Tyr-2,3	^{2}Tyr-2,2-NH,3,5,5’
4	127.4 C		^{2}Tyr-2,3,3’,5,5’		
5,5’	130.3 CH	7.00 d, 8.1	^{2}Tyr-3,3’,5’,5	^{2}Tyr-6,6’	^{2}Tyr-2,3,3’,6,6’
6,6’	115.1 CH	6.63 d, 8.1	^{2}Tyr-5,5’,6’,6	^{2}Tyr-5,5’	^{2}Tyr-2-NH,5,5’,7-OH
7	156.1 C		^{2}Tyr-5,5’,6,6’		
7-OH		9.17 s			^{2}Tyr-6,6’

S98. ^{1}H NMR Spectrum of Microginin FR4 (**10**) in DMSO-d_6

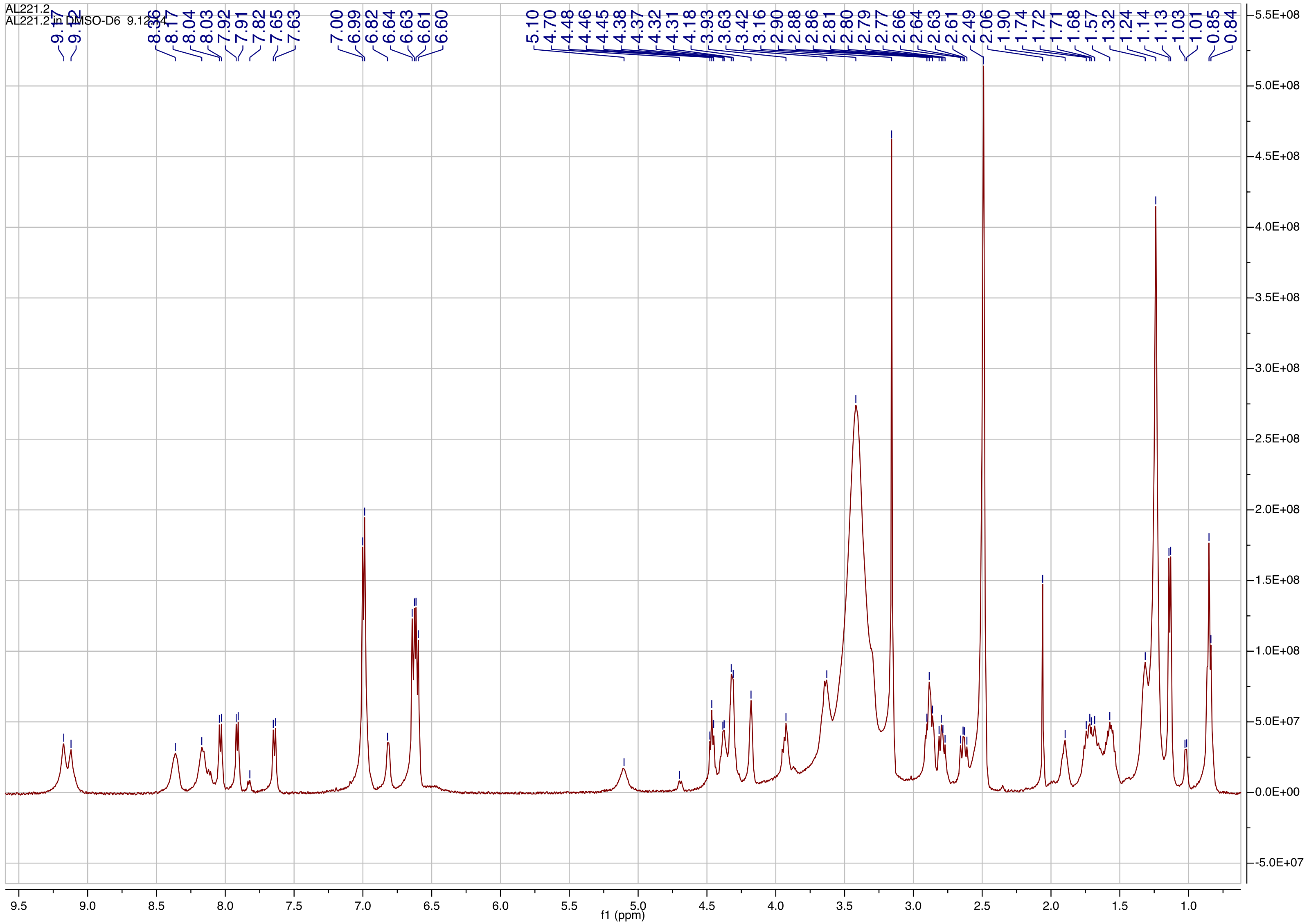

S99. ^{13}C NMR Spectrum of Microginin FR4 (**10**) in DMSO-d_6

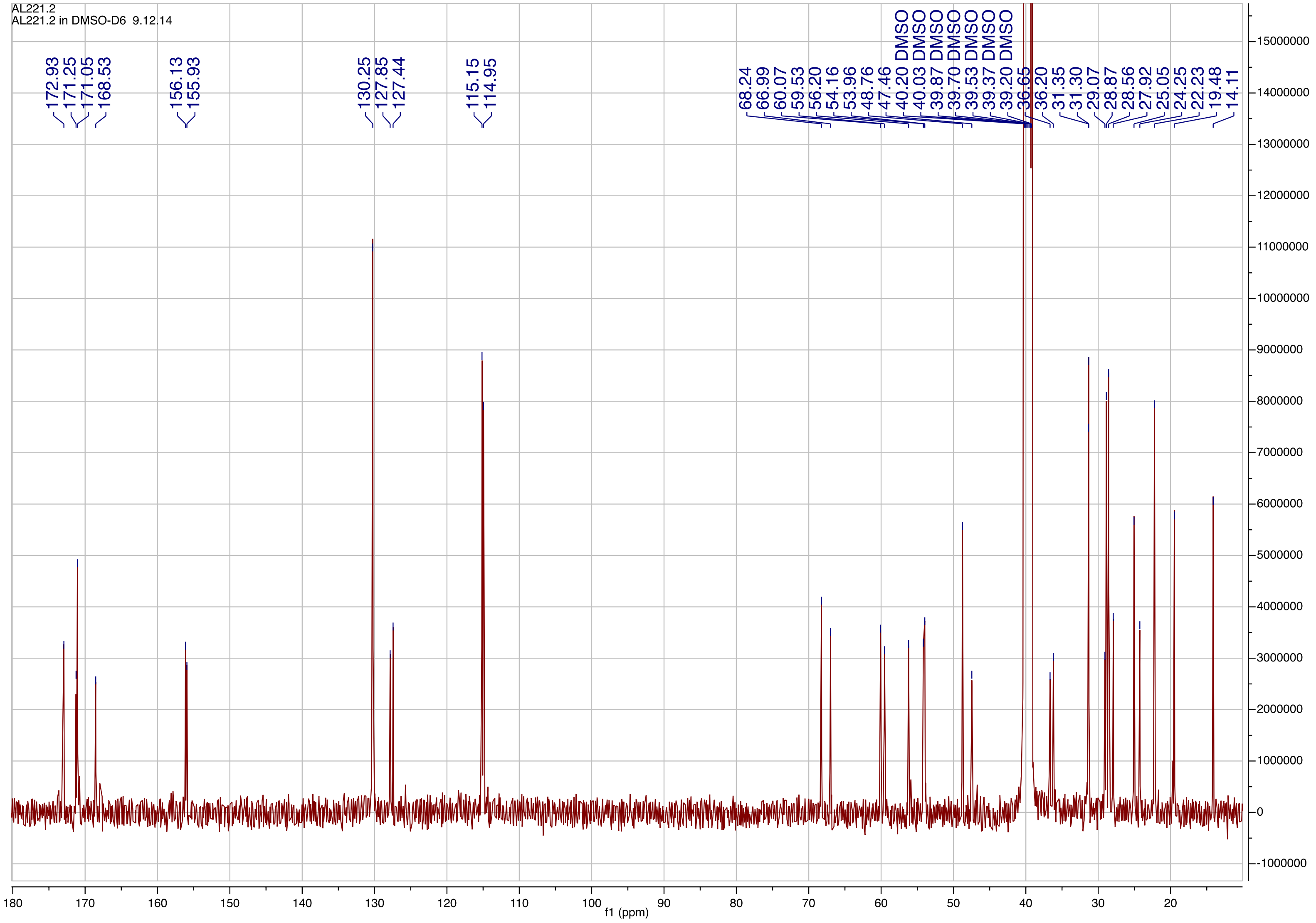

S100. HSQC Spectrum Microginin FR4 (**10**) in DMSO-d_6

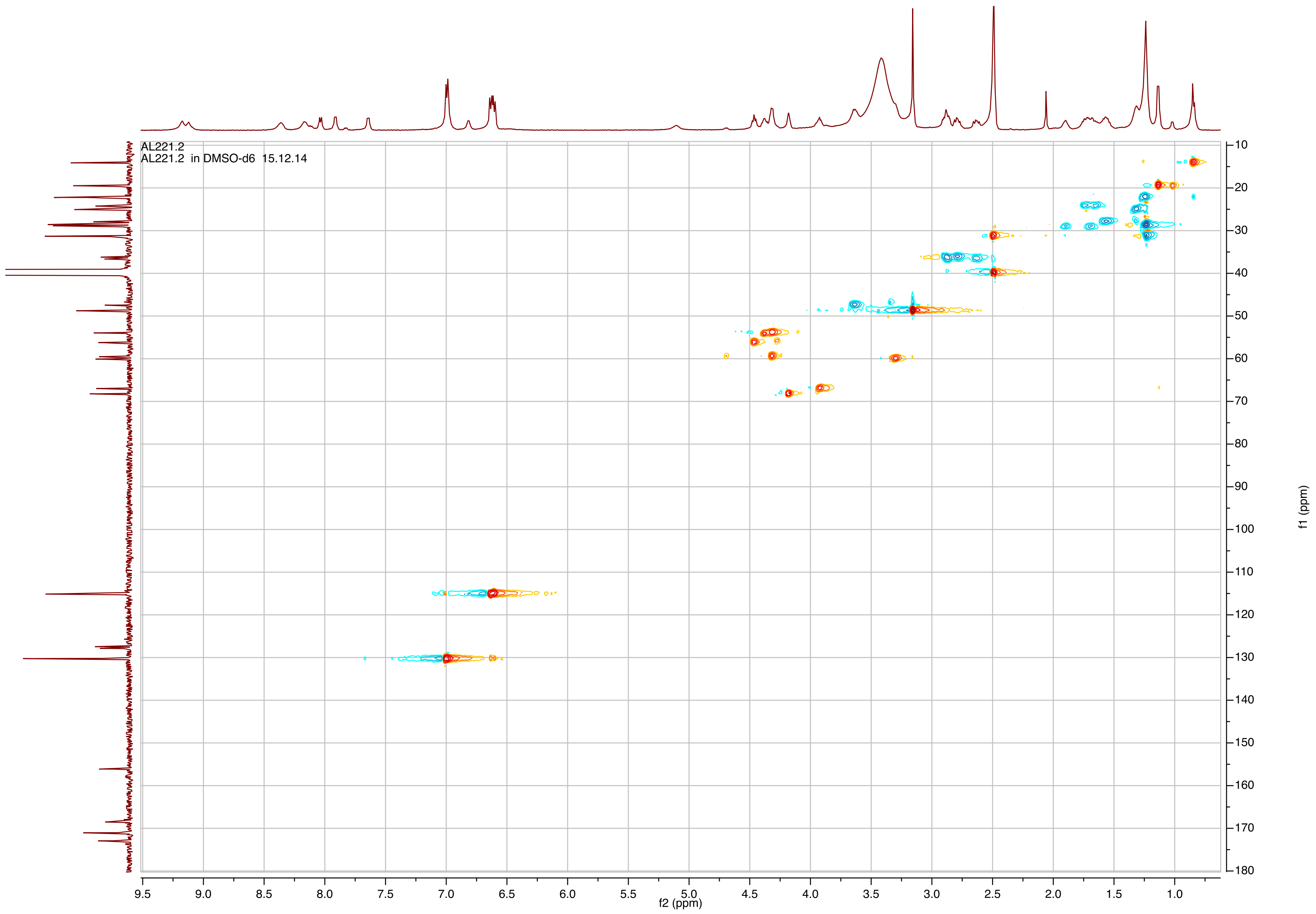

S101. HMBC Spectrum of Microginin FR4 (**10**) in DMSO-d_6

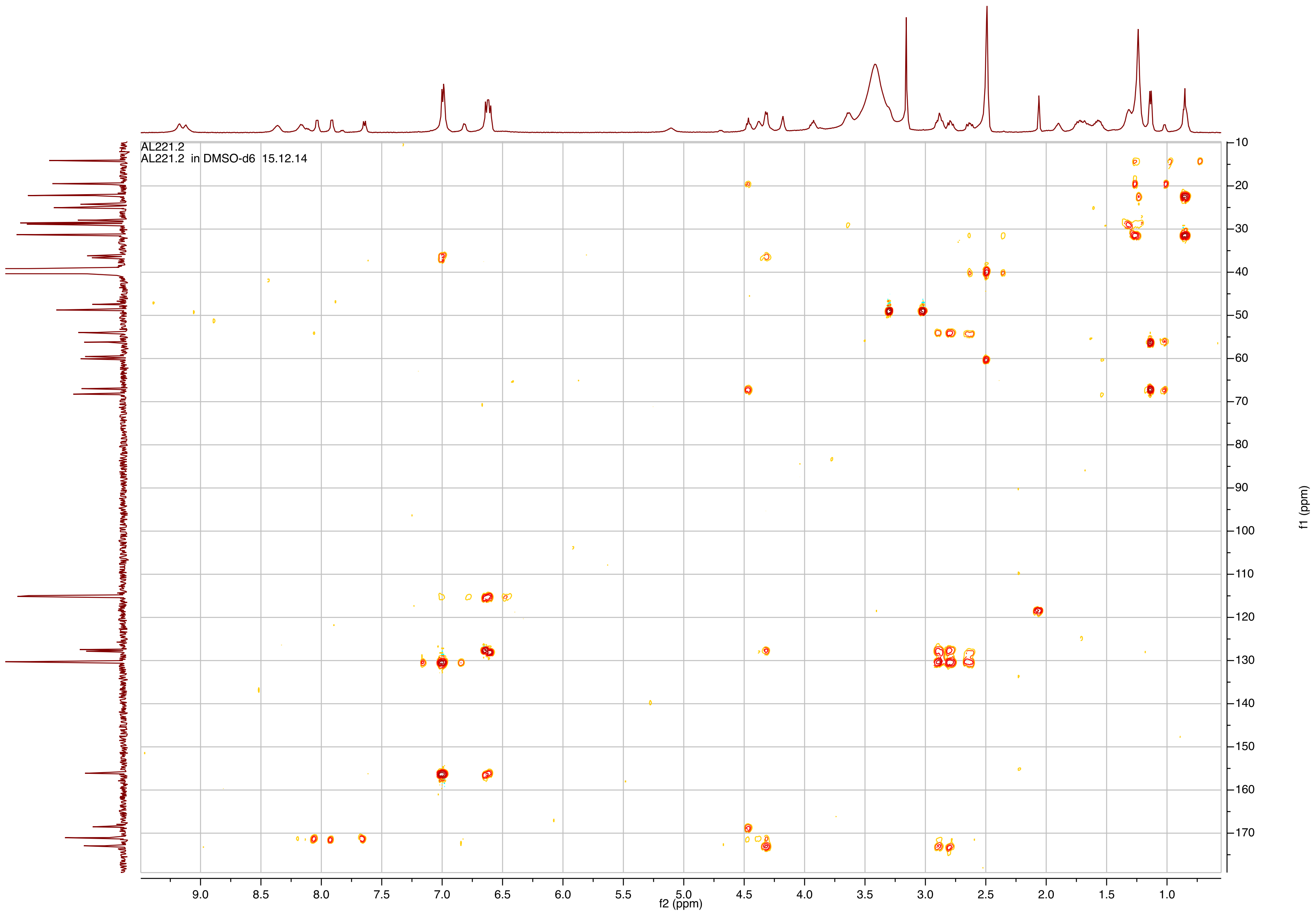

S102. COSY Spectrum of Microginin FR4 (**10**) in DMSO-d_6

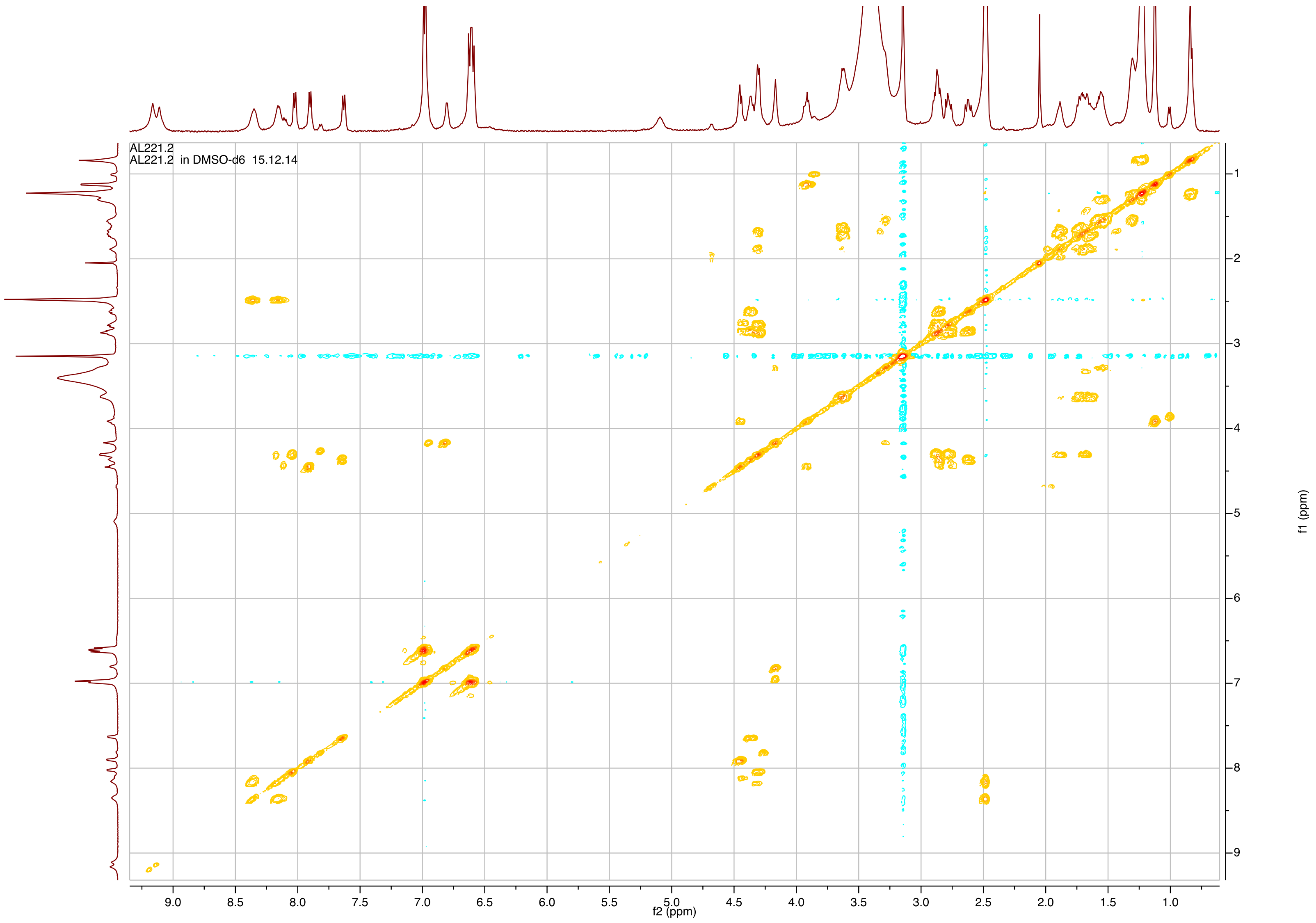

S103. TOCSY Spectrum of Microginin FR4 (**10**) in DMSO-d_6

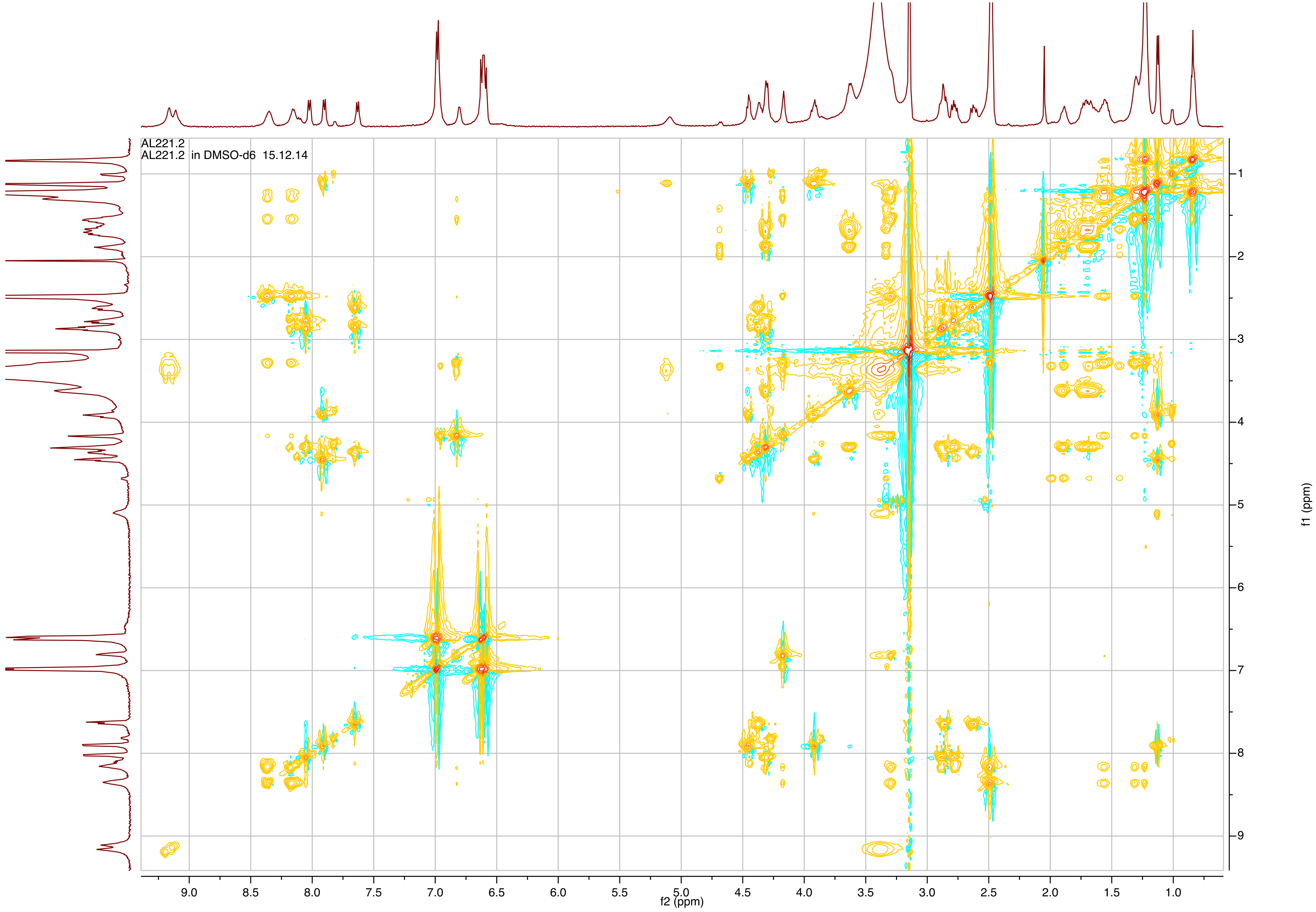

S104. ROESY Spectrum of Microginin FR4 (**10**) in DMSO-d_6

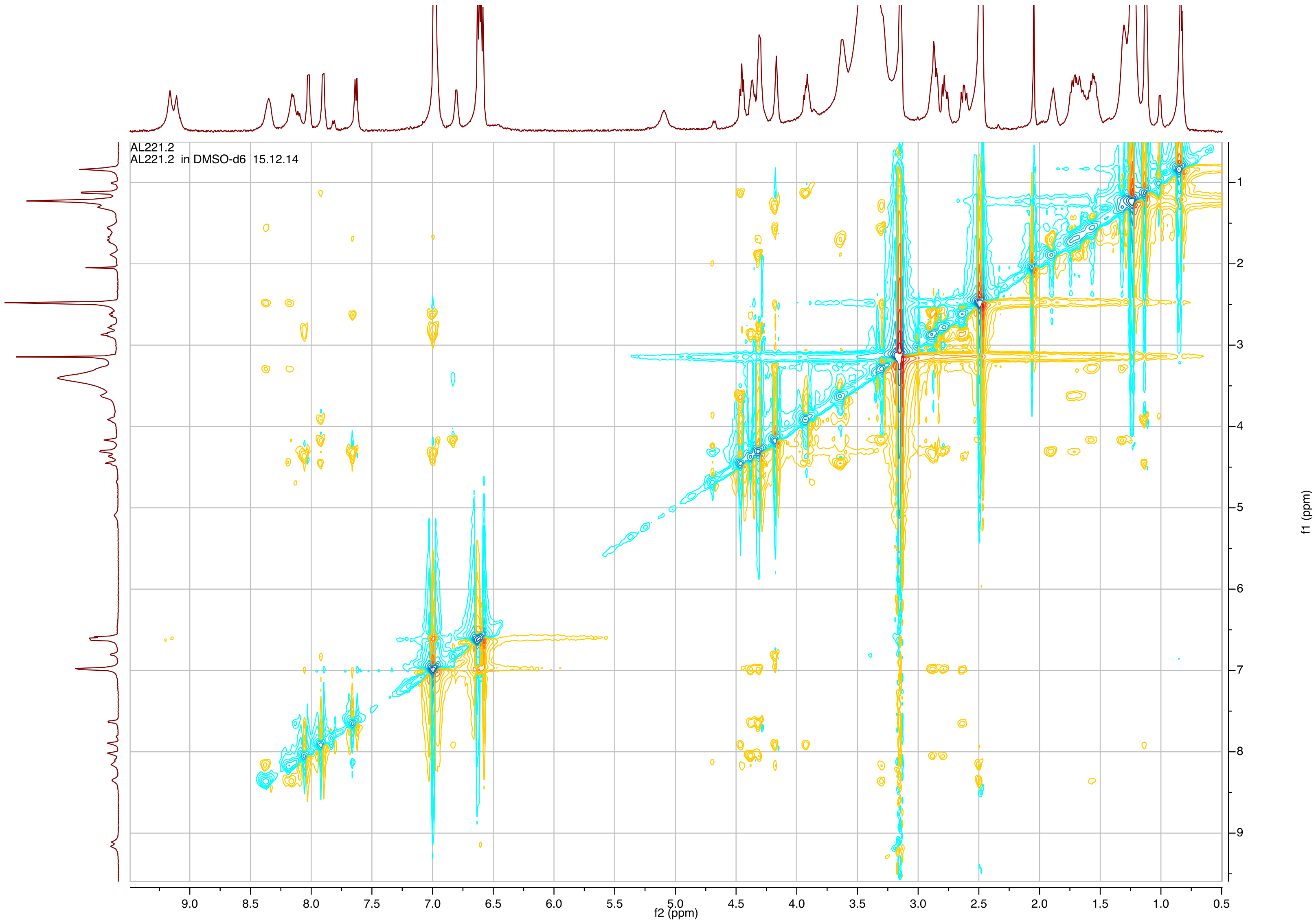

S105. HR ESI MS data of Microginin FR4 (**10**)

Elemental Composition Report **Page 1**

Single Mass Analysis
Tolerance = 2.0 PPM / DBE: min = -1.5, max = 50.0
Element prediction: Off
Number of isotope peaks used for i-FIT = 3

Monoisotopic Mass, Even Electron Ions
138 formula(e) evaluated with 2 results within limits (all results (up to 1000) for each mass)
Elements Used:
C: 35-45 H: 50-65 N: 0-10 O: 0-15
AL221.2
carmeli867 48 (2.121) Cm (48:51)
Anat Lodin

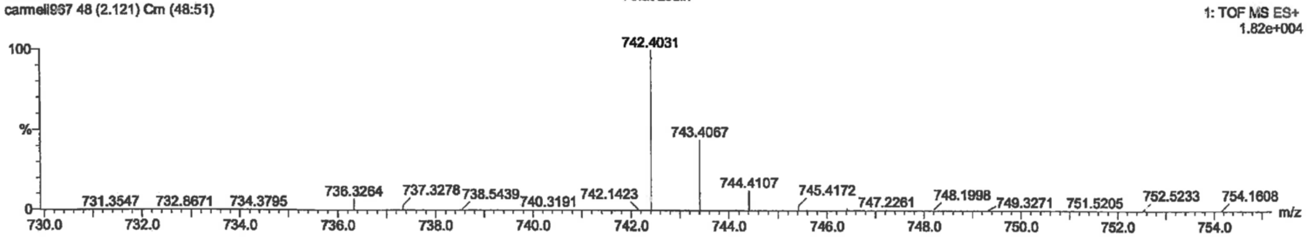

Minimum:				-1.5			
Maximum:		5.0	2.0	50.0			
Mass	Calc. Mass	mDa	PPM	DBE	i-FIT	i-FIT (Norm)	Formula
742.4031	742.4027	0.4	0.5	13.5	167.2	0.0	C38 H56 N5 O10
	742.4041	-1.0	-1.3	18.5	170.4	3.3	C39 H52 N9 O6
